# Extended-Release vs Sublingual Buprenorphine in Pregnancy Through 12 Months Post Partum: A Randomized Clinical Trial

**DOI:** 10.1001/jamainternmed.2026.0057

**Published:** 2026-03-16

**Authors:** T. John Winhusen, Michelle R. Lofwall, Frankie Kropp, Daniel Lewis, Marcela C. Smid, Jessica L. Young, Candace Hodgkins, Elizabeth E. Krans, Zachary Hansen, Elisha M. Wachman, Davida M. Schiff, Constance Guille, Vania Rudolf, Tara Chowdhury, Lawrence Leeman, Mitra Lewis, Abigail G. Matthews, Gerald Cochran, Jacquie King, Christine Wilder, Carmen Rosa

**Affiliations:** 1 Department of Psychiatry and Behavioral Neuroscience, University of Cincinnati College of Medicine, Cincinnati, Ohio; 2 University of Cincinnati/UC Health Addiction Center, Cincinnati, Ohio; 3 Departments of Behavioral Science and Psychiatry, Center on Drug and Alcohol Research, University of Kentucky College of Medicine, Lexington; 4 Department of Obstetrics and Gynecology, University of Utah Health, Salt Lake City; 5 Department of Obstetrics and Gynecology, Vanderbilt University Medical Center, Nashville, Tennessee; 6 Gateway Community Services, Jacksonville, Florida; 7 Department of Obstetrics, Gynecology, and Reproductive Sciences, and Magee-Womens Research Institute, University of Pittsburgh School of Medicine, Pittsburgh, Pennsylvania; 8 Department of Family Medicine, Division of Addiction Science, Marshall University, Huntington, West Virginia; 9 Department of Pediatrics, Boston Medical Center, Boston, Massachusetts; 10 Divisions of General Academic Pediatrics and Newborn Medicine, Mass General for Children, Boston, Massachusetts; 11 Department of Psychiatry and Behavioral Science, Medical University of South Carolina, Charleston; 12 Addiction Recovery Services, Swedish Medical Center, Seattle, Washington; 13 CODA, Inc, Portland, Oregon; 14 Department of Family and Community Medicine, University of New Mexico School of Medicine, Albuquerque; 15 The Emmes Company, LLC, Rockville, Maryland; 16 Department of Internal Medicine, University of Utah, Salt Lake City; 17 Perinatal Addiction Program, University of Cincinnati Health, Cincinnati, Ohio; 18 Now with Hazelden Betty Ford Foundation, Center City, Minnesota; 19 Center for the Clinical Trials Network, National Institute on Drug Abuse, Bethesda, Maryland (now retired)

## Abstract

**Importance:**

Treating opioid use disorder (OUD) in pregnancy with sublingual buprenorphine is an evidence-based practice, but it has disadvantages that could be addressed with an extended-release formulation.

**Objective:**

To evaluate the effectiveness and safety of extended-release buprenorphine vs sublingual buprenorphine for OUD in pregnancy through 12 months post partum.

**Design, Setting, and Participants:**

This 2-group, open-label, noninferiority, randomized clinical trial was conducted between July 2, 2020, and October 30, 2024, among adults with OUD and a singleton pregnancy of 6 to 30 weeks’ gestational age at 13 outpatient cross-disciplinary peripartum OUD treatment sites.

**Interventions:**

Randomization to sublingual or extended-release buprenorphine (weekly formulation during pregnancy, monthly formulation optional post partum if not breastfeeding).

**Main Outcomes and Measures:**

The primary and key secondary outcomes were illicit opioid abstinence during pregnancy and the postpartum period, respectively, defined as the proportion of weekly collected urine samples negative for illicit opioids. If noninferiority was demonstrated at a margin of 0.15, testing for superiority was planned. Key secondary infant outcomes from medical records were opioid treatment for neonatal opioid withdrawal syndrome (NOWS; yes or no) and number of opioid treatment days for NOWS.

**Results:**

Among 140 randomized participants, the mean (SD) age was 31.2 (4.6) years. There were 10 Black participants (7.1%), 10 Hispanic participants (7.1%), 116 (82.9%) White participants, and 14 participants (10.0%) who belonged to additional groups. All but 2 were already prescribed sublingual buprenorphine. Study completion was 98% through pregnancy (137 participants) and 81% through 12 months post partum (114 participants). Illicit opioid abstinence was higher during pregnancy for participants receiving extended-release vs sublingual buprenorphine (82.5% vs 72.6%; mean difference, 9.84 [95% CI, 1.72 to 17.95] percentage points; *P* = .009). Postpartum abstinence rates declined and were similar in both groups (60.2% vs 59.5%; mean difference, 0.65 [98% CI, −12.72 to 14.02] percentage points; *P* = .45). Those receiving extended-release buprenorphine experienced fewer serious adverse events during pregnancy (8.7% vs 26.8%; *P* = .007) and post partum (6.0% vs 18.6%; *P* = .04). Nonserious adverse events rates did not differ between groups, but more were deemed medication-related for extended-release participants during pregnancy (26.1% vs 7.0%; *P* = .003). Infants exposed to extended-release vs sublingual buprenorphine did not differ in need for opioid treatment (30.2% vs 26.5%; relative risk, 1.14 [98% CI, 0.54 to 1.99]; *P* = .64) or mean (SE) treatment days (10.9 [2.2] vs 14.8 [3.0] days; relative risk, 0.73 [98% CI, 0.36 to 1.51]; *P* = .28). At birth, extended-release–exposed neonates had larger mean (SE) head circumferences than those exposed to sublingual buprenorphine (34.0 [0.2] vs 33.4 [0.2] cm; mean difference, 0.63 [95% CI, −0.00 to 1.26] cm; *P* = .049).

**Conclusions and Relevance:**

The findings of this randomized clinical trial support weekly extended-release buprenorphine for OUD treatment during pregnancy.

**Trial Registration:**

ClinicalTrials.gov Identifier: NCT03918850

## Key Points

**Question:**

Does weekly subcutaneous extended-release buprenorphine treatment for opioid use disorder (OUD) in pregnancy produce noninferior illicit opioid abstinence rates and superior neonatal opioid withdrawal syndrome (NOWS) outcomes vs sublingual buprenorphine?

**Findings:**

In this randomized clinical trial with 140 pregnant adults, illicit-opioid abstinence in the extended-release group was statistically superior to that in the sublingual group. NOWS outcomes and maternal adverse events (AEs) did not differ between treatments, although the latter were more commonly rated as medication-related in the extended-release group during pregnancy; however, maternal serious AEs were less common in the extended-release group throughout the trial.

**Meaning:**

These findings support using weekly extended-release buprenorphine for OUD during pregnancy.

## Teaser

This randomized clinical trial evaluates whether weekly extended-release buprenorphine has noninferior effectiveness and safety outcomes compared with sublingual buprenorphine among pregnant adults with opioid use disorder.

## Introduction

Sublingual buprenorphine is a standard treatment for opioid use disorder (OUD) in peripartum persons^1^ but has disadvantages, including risk of diversion and misuse,^2^ poor adherence,^3^ and daily peak-trough effects that may inadequately mitigate opioid-related cravings and withdrawal,^4^ especially during pregnancy.^5^ Extended-release buprenorphine helps address these disadvantages and has demonstrated superior illicit opioid abstinence in nonpregnant adults.^6^ A potential concern of extended-release buprenorphine use in pregnancy is the higher buprenorphine exposure (ie, area under the curve) for this formulation, relative to comparable doses of sublingual buprenorphine,^7^ translating into greater fetal buprenorphine exposure. While the published literature includes several case and cohort studies of extended-release buprenorphine in pregnant persons,^8,9,10,11,12,13^ there have been no completed randomized clinical trials.

## Methods

### Trial Design

This intent-to-treat, 2-group, open-label, noninferiority randomized clinical trial evaluated the effectiveness and safety of extended-release buprenorphine relative to sublingual buprenorphine. The trial was pragmatic in that it was open label; was conducted in geographically varied clinical settings with existing collaborative care models; allowed site-preferred sublingual buprenorphine formulations and site-standard induction and dosing practices; relied heavily on medical record data rather than extensive research-only assessments; and allowed variation in hospital NOWS practices. The trial protocol is in Supplement 1. The protocol followed the Consolidated Standards of Reporting Trials (CONSORT) guideline and was approved by the Food and Drug Administration, the data and safety monitoring board of the National Institute on Drug Abuse Treatment Clinical Trials Network, and by a single institutional review board (IRB) of record, the University of Cincinnati IRB. The trial was conducted at 13 US sites between July 2, 2020, and October 30, 2024; 1 site, which was closed for poor recruitment, never randomized participants. Study sites had close collaboration between prenatal care and addiction treatment clinicians.^14^ Most were specialty obstetrics and gynecology programs providing OUD services affiliated with an academic institution.^14^ Participants received study medication and attended weekly visits through 12 months post partum. Longer research visits occurred 2 weeks after randomization and then monthly until delivery and at months 1, 3, 6, 9, and 12 during the postpartum phase. Visits could occur onsite or offsite, and some elements (eg, self-report assessments) could be completed via telehealth. Participants provided written informed consent and were compensated for participating.

### Participants

Participants were aged 18 to 41 years, with a singleton pregnancy of estimated gestational age (EGA) between 6 and 30 weeks at randomization, meeting *Diagnostic and Statistical Manual of Mental Disorders*, *Fifth Edition* (*DSM-5*) criteria for moderate or severe OUD and eligible for buprenorphine treatment and/or already prescribed buprenorphine. They were planning for delivery at a hospital that, based on a completed Better Outcomes Through Research for Newborns survey,^15^ met 3 requirements designed to reduce variability in procedures that can impact neonatal opioid withdrawal syndrome (NOWS) severity and outcome measures^16,17^: (1) had a written NOWS management protocol; (2) offered rooming-in for infants being observed for NOWS; and (3) did not discharge infants receiving opioid treatment. Exclusion criteria included physiological dependence on alcohol or sedatives requiring medically managed withdrawal; having a psychiatric or medical condition that may make study participation difficult or unsafe; current or pending criminal justice involvement that could interfere with study participation; receiving methadone or naltrexone treatment; or current or planned enrollment in treatment beyond level 3.3 of the American Society of Addiction Medicine Criteria.^18^

Per National Institutes of Health requirements, race and ethnicity were assessed. Participants were asked whether they considered themselves to be Hispanic or Latino (yes or no) and what race they considered themselves to be. The participant’s response was recorded by a research staff member with race categorized into the following: American Indian or Alaska Native, Asian, Black or African American, Native Hawaiian or Pacific Islander, White, or some other race; the latter category reflects respect for participant self-identification and autonomy.

### Intervention Groups and Medication Dosing

Participants were randomized 1:1 to extended-release or sublingual buprenorphine, balancing on site, EGA at randomization (6-18 weeks vs 19-30 weeks), and whether they were already receiving sublingual buprenorphine (yes or no). Study-purchased generic sublingual buprenorphine was provided as tablets and/or buprenorphine/naloxone film, based on site and participant preference. Braeburn donated extended-release buprenorphine (CAM2038/Brixadi). The target dose was 16 mg/d of sublingual buprenorphine or the equivalent extended-release buprenorphine weekly dose (24 mg), consistent with recommended dosing during pregnancy,^19^ but dosing was determined by the clinician. Pregnant and breastfeeding participants in the extended-release buprenorphine group received weekly injections due to concerns about excipient (ie, N-methyl-2-pyrrolidone) in monthly formulations increasing risk for adverse fetal developmental effects^20^; if not breastfeeding, monthly injections were offered. Between December 20, 2021, and May 10, 2022, a supply disruption in extended-release buprenorphine paused site enrollment and led 2 pregnant and 14 postpartum participants to stop study medication; all were offered sublingual buprenorphine. eTable 1 in Supplement 2 details dosing before, during, and after the supply disruption.

### Assessment

The primary outcome was illicit opioid abstinence during pregnancy, defined as the proportion of urine drug screens negative for fentanyl, morphine, codeine, ethylmorphine, heroin, hydrocodone, hydromorphone, methadone, and oxycodone. Consistent with a CAM2038 phase 3 trial,^6^ results missing for any reason were imputed as positive for illicit opioids. Urine samples were collected weekly throughout the study. Postpartum illicit opioid abstinence was the maternal key secondary outcome. Infant key secondary outcomes were opioid treatment for NOWS (yes or no) and if yes, the number of days of opioid treatment, both extracted from medical records. Maternal secondary outcomes were collected during pregnancy and post partum, except Kotelchuck’s Adequacy of Prenatal Care Utilization index,^21^ which was based on medical records and combines prenatal care start timing with the ratio of observed-to-expected visits. Buprenorphine adherence was calculated as days adherent divided by active treatment study days (regardless of study or medication discontinuation); for participants in the extended-release buprenorphine group, days affected by the supply disruptions were excluded. Adherence was assessed weekly and included both study-provided and nonstudy buprenorphine (ie, clinically provided outside of the study). Extended-release buprenorphine adherence was defined as 7 days of adherence per weekly injection and 28 days per monthly injection. Sublingual buprenorphine adherence was defined as receipt of study dispensation or prescription and self-reported use, reduced by 7 days for each negative buprenorphine and norbuprenorphine urine result. Drug and alcohol abstinence was defined as the proportion of urine drug screens negative for illicit opioids, cocaine, methamphetamine, amphetamine, cannabis, benzodiazepines, barbiturates, phencyclidine, methylenedioxymethamphetamine, and ethyl glucuronide. According to the package inserts for the urine drug screen tests used in this study, comparisons with confirmatory gas chromatography–mass spectrometry (MS) or liquid chromatography–tandem MS testing showed a 0% false-positive rate. Although false positives can occur in clinical settings, this risk is minimal and did not justify the added cost of confirmatory testing for this trial. Outcomes collected during research visits were the (1) Opioid Craving Scale (each of 3 items scored from 0-10 and averaged, with higher scores indicating more craving)^22^ and (2) Short Opiate Withdrawal Scale–Gossop (10 items each scored from 0-3 to give total scores ranging from 0-30, with higher scores indicating more withdrawal).^23^

Of the 6 secondary infant outcomes, 3 NOWS-related outcomes and 2 discharge status outcomes came from medical records. NOWS outcomes included length of hospital stay (days old at discharge), peak NOWS score on the Modified Finnegan^21^ (the only scoring method with sufficient data for analysis), and for morphine-treated infants, total milligrams’ morphine received. Hospital discharge outcomes included maternal custody and whether a case was open with child protective services (CPS). The sixth infant outcome indicated any potential delay at 12 months in communication, gross motor, fine motor, problem solving, or personal-social development assessed by the 12-month version of the Ages and Stages Questionnaire, Third Edition, which scores each item as yes (10 points), sometimes (5 points), or not yet (0 points), with total scores compared with cutoffs to identify delays.^24^

Maternal safety measures assessed weekly included reported adverse events (AEs) and injection site examinations for extended-release buprenorphine participants, with the latter not captured as AEs. AEs were classified using Medical Dictionary for Regulatory Activities (MedDRA) version 27.1. Maternal safety measures at research visits included self-reported opioid overdoses—defined as an overdose causing unresponsiveness or inadequate breathing, resulting in naloxone rescue and/or emergency medical care or hospitalization^25^—and the Hospital Anxiety and Depression Scale, which has 7 items each for depression and anxiety scored from 0 to 3, with total scores ranging 0 to 21, and higher scores indicating more severe anxiety and depression symptoms.^26^ Other maternal safety outcomes—including primary cesarean delivery; abnormal fetal presentation; labor complications; and use of pain medication during labor and delivery, postpartum hospitalization, or prescribed at discharge—were obtained from medical records.

Infant safety measures assessed weekly post partum included participant-reported serious AEs and infant sedation signs, such as not waking for feeding, difficulty breathing (beyond a stuffy nose), or being limp when held. Additional medical record outcomes were live birth, EGA at delivery, birth head circumference, weight, length, Apgar scores at 1 and 5 minutes, presence of abnormal conditions (eg, respiratory distress, feeding issues), related interventions (eg, resuscitation, assisted ventilation), preterm status (<37 weeks), and discharged alive.

### Statistical Analysis

The statistical analysis plan (Supplement 1) used the intent-to-treat sample and was finalized before analyses were undertaken. The Bonferroni method set the family-wise α level. Primary outcome noninferiority and superiority tests used a 1-tailed α = .025. The 3 key secondary analyses used α = .05/3 = .0167, and the 15 secondary analyses used α = .05/15 = .0033. Safety analyses applied α = .05 to detect safety differences. SAS version 9.4 (SAS Institute) was used to conduct analyses.

For primary and maternal key secondary outcomes, data were truncated at the supply disruption for affected extended-release buprenorphine participants, analyzed via mixed-model regression with treatment cohort as the main effect and random effects for site and baseline EGA of less than 19 weeks. If noninferiority was demonstrated, superiority testing followed. Infant key secondary analyses excluded infants of mothers who experienced supply disruptions for extended-release buprenorphine and used regressions adjusting for treatment cohort and fixed effects (site and baseline EGA if significant).

Secondary and safety outcomes included data during the supply disruption, with regressions adjusting for treatment cohort, fixed effects (site and baseline EGA if significant), and baseline outcome values. Sensitivity analyses incorporated postdisruption data and assessed covariate impact per the statistical analysis plan. Fisher exact tests compared AEs between groups.

The target sample size was 300 based on a primary outcome noninferiority margin of 0.11, 65% sublingual buprenorphine illicit opioid abstinence, and a 0.11 variance in both groups.^20^ During early implementation, the noninferiority margin was deemed too conservative and the power calculations were repeated with a margin of 0.15^27^; the sample size was reduced to 200. Due to slow enrollment during the COVID-19 pandemic and paused enrollment during the extended-release buprenorphine supply disruption, sample size was reestimated in January 2022 and again in March 2023. The first reestimation used the variance from the first 64 participants, and the second used the abstinence rate and variance from the 52 completed sublingual buprenorphine pregnancies; type I error rate was not impacted. Both reestimations demonstrated at least 84% power to detect a noninferiority margin of 0.15 with a sample size of 126.

## Results

### Participants

One hundred forty participants were randomized to extended-release buprenorphine (n = 69) or sublingual buprenorphine (n = 71) (Figure). Pregnancy and postpartum phases were completed by 98% and 81% of participants (137 and 114 participants), respectively, with no group differences. Table 1 provides participant demographic and clinical characteristics. The mean (SD) age was 31.2 (4.6) years; there were 10 Black participants (7.1%), 10 Hispanic participants (7.1%), 116 (82.9%) White participants, and 14 participants (10.0%) who belonged to additional groups; and 47 (33.6%) were employed. Mean (SD) baseline EGA was 21.1 (6.5) weeks. All but 2 participants were receiving sublingual buprenorphine at randomization.

**Figure.**
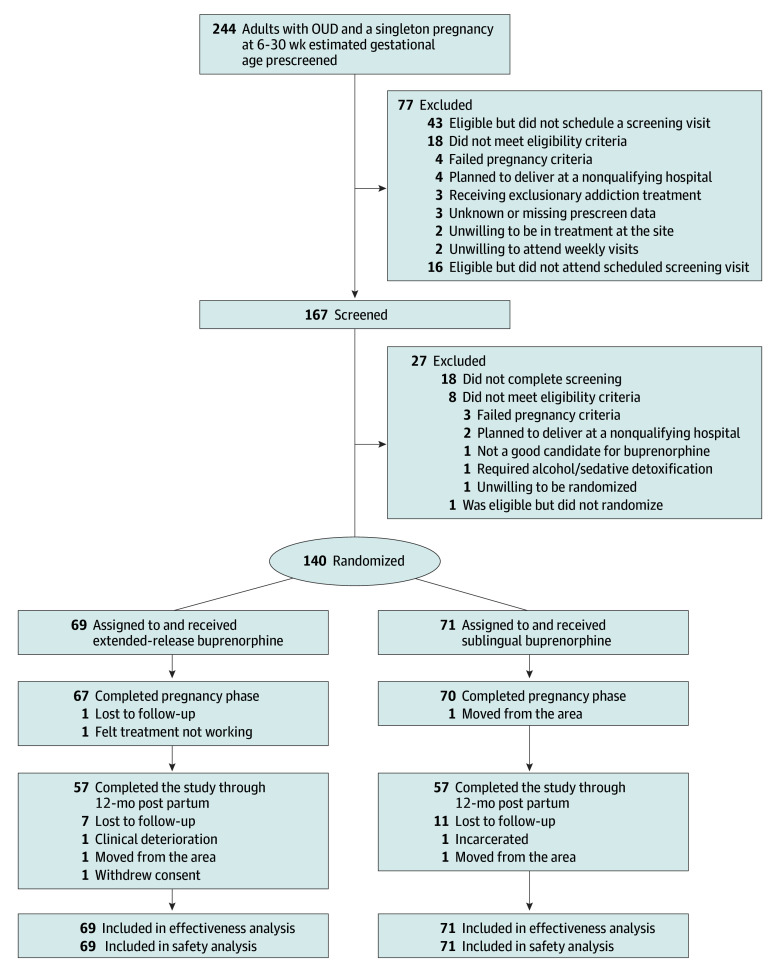
Flowchart of Study Enrollment and Retention OUD indicates opioid use disorder.

**Table 1.** Baseline Characteristics of Randomized Participants by Study Treatment Group

Characteristic	Participants by buprenorphine treatment group, No. (%)^a^		All participants, No. (%) (N = 140)
	Extended release (n = 69)	Sublingual (n = 71)	
Age, mean (SD), y	31.1 (4.6)	31.4 (4.7)	31.2 (4.6)
Hispanic or Latina ethnicity^b^	4 (5.8)	6 (8.5)	10 (7.1)
Race^b^			
Black or African American	3 (4.3)	7 (9.9)	10 (7.1)
Additional groups	7 (10.1)	7 (9.9)	14 (10.0)
White	59 (85.5)	57 (80.3)	116 (82.9)
Estimated gestational age of fetus, mean (SD), wk	21.5 (6.5)	20.6 (6.6)	21.1 (6.5)
High school diploma, GED, or less^c^	43 (64.2)	35 (50.7)	78 (57.4)
Employed^d^	26 (37.7)	21 (29.6)	47 (33.6)
Married^e^	10 (14.5)	11 (16.2)	21 (15.3)
Substances for which urine drug screen was positive for ≥5% of treatment group			
Alcohol	5 (7.2)	0	5 (3.6)
Amphetamine	11 (15.9)	9 (12.7)	20 (14.3)
Benzodiazepines	0	5 (7.0)	5 (3.6)
Buprenorphine	69 (100.0)	70 (98.6)	139 (99.3)
Cannabis	11 (15.9)	13 (18.3)	24 (17.1)
Cocaine	3 (4.3)	4 (5.6)	7 (5.0)
Fentanyl	6 (8.7)	6 (8.5)	12 (8.6)
Methamphetamine	9 (13.0)	6 (8.5)	15 (10.7)
Opiates	2 (2.9)	4 (5.6)	6 (4.3)
Cotinine	57 (82.6)	62 (87.3)	119 (85.0)
Taking buprenorphine at randomization	68 (98.6)	70 (98.6)	138 (98.6)
Time taking buprenorphine, median (IQR), d^f^	66.0 (34.0-290.0)	72.5 (23.8-243.5)	67.0 (29.0-251.5)
Participants reporting intravenous illicit opioid use ≥1 d in the 30 d before consent	6 (8.7)	4 (5.6)	10 (7.1)
*DSM-5* use disorders (past 12 mo)			
Alcohol	13 (18.8%)	13 (18.3%)	26 (18.6%)
Amphetamine	30 (43.5%)	29 (40.8%)	59 (42.1%)
Cannabis	24 (34.8%)	23 (32.4%)	47 (33.6%)
Cocaine	16 (23.2%)	17 (23.9%)	33 (23.6%)
Opioids	57 (82.6%)	49 (69.0%)	106 (75.7%)
Sedatives	10 (14.5%)	12 (16.9%)	22 (15.7%)

Abbreviations: *DSM-5*, *Diagnostic and Statistical Manual of Mental Disorders, Fifth Edition*; GED, General Educational Development diploma.

^a^ Percentages may not total to 100 because of rounding.

^b^ Race and ethnic group were reported by the participants. The additional groups category included 3 American Indian or Alaska Native, 1 Asian, 1 Native Hawaiian or Pacific Islander, and 7 multiracial individuals as well as 2 who identified as another racial group.

^c^ Reported by 67 participants in the extended-release group and 69 in the sublingual group.

^d^ The remaining participants were temporarily laid off or on leave, unemployed, disabled, keeping house, or students or had other status.

^e^ Reported by 69 participants in the extended-release group and 68 in the sublingual group; the remaining participants were divorced, separated, never married, or living with partner.

^f^ Data available for 127 participants (63 in the extended release group and 64 in the sublingual group) of the 138 participants taking buprenorphine at randomization.

### Medication Exposure

During pregnancy, for the dose closest to delivery or at medication disruption, 41 participants in the extended-release buprenorphine group (59%) were receiving 32 mg, the highest dose, which is approximately equivalent to 18 to 24 mg of sublingual buprenorphine. For the sublingual buprenorphine group, 49 (69%) received buprenorphine-only tablets and 61 (86%) dosed multiple times per day; the mean (SD) daily dose was 18.8 (6.7) mg. Medication was discontinued by 8 participants in the extended-release buprenorphine group (12%) and 6 in the sublingual buprenorphine group (9%) during pregnancy. During the postpartum phase, for the last dose or the dose at medication disruption, 34 participants in the extended-release buprenorphine group (58%) were receiving the monthly injection. For those receiving weekly injections, the highest dose (32 mg) was the most frequent, accounting for 44% of the sample (11 of 25 participants). For those receiving monthly injections, the highest dose (128 mg) was the most frequent, accounting for 65% of the sample (22 of 34 participants). For the sublingual buprenorphine group, 28 of 65 (43%) received buprenorphine-only tablets and 54 (83%) dosed multiple times per day; the mean (SD) daily dose was 18.2 (6.8) mg. Medication was discontinued by 25 extended-release buprenorphine (37%) and 23 sublingual buprenorphine (33%) participants post partum. eTable 2 in Supplement 2 provides additional dosing information.

### Effectiveness Outcomes

Both the maternal primary and key secondary outcomes met prespecified criteria for noninferiority (Table 2). Superiority analyses demonstrated superiority of extended-release buprenorphine for illicit opioid abstinence during pregnancy (primary outcome: mean [SE] negative urine samples, 82.5% [4.2] vs 72.6% [4.2]; mean difference, 9.84 [95% CI, 1.72 to 17.95] percentage points; *P* = .009) but not post partum (key secondary outcome: mean [SE] negative urine samples, 60.2% [4.2] vs 59.5% [4.1]; mean difference, 0.65 [98% CI, −12.72 to 14.02] percentage points; *P* = .45). Significant group differences were not found for any other maternal effectiveness outcomes (Table 2). Table 3 shows infant effectiveness results. There were no significant group differences at the preestablished α levels for any outcome, including the key secondary outcomes of need for opioid treatment for NOWS (mean [SE] proportion treated with opioids: 30.2% [5.8] vs 26.5% [5.4]; relative risk, 1.14 [98% CI, 0.54 to 1.99]; *P* = .64) and mean (SE) days of opioid treatment (10.9 [2.2] vs 14.8 [3.0] days; relative risk, 0.73 [98% CI, 0.36 to 1.51]; *P* = .28). All sensitivity analyses produced comparable results.

**Table 2.** Maternal Effectiveness and Significant Safety Outcomes as a Function of Study Treatment Group

Outcome	Participants by study treatment group, mean (SE)		Effect size (CI)^a^	*P* value
	Extended release	Sublingual		
**Effectiveness outcomes: pregnancy**				
Total No.	69	71	NA	NA
Illicit opioid–negative urine samples, % UDS^b^^,^^c^	82.5 (4.2)	72.6 (4.2)	9.84 (1.72 to 17.95) pp	.009
Buprenorphine adherence, % days	84.4 (3.1)	84.9 (3.0)	−0.51 (−13.32 to 12.31) pp	.91
Drug and alcohol abstinence, % UDS^d^	58.8 (3.4)	52.2 (3.4)	4.42 (−10.02 to 18.86) pp	.36
Opioid Craving Scale^e^	1.9 (0.2)	2.5 (0.2)	−0.31 (−1.23 to 0.61)	.31
Short Opiate Withdrawal Scale^e^	5.6 (0.5)	6.2 (0.5)	−1.11 (−3.38 to 1.16)	.14
Adequacy of prenatal care utilization, %^f^				
Inadequate	36.7 (8.9)	19.4 (6.7)	OR, 2.56 (0.61 to 10.73)	.05
Intermediate	13.3 (6.3)	16.7 (6.3)		
Adequate	23.3 (7.9)	13.9 (5.8)		
Adequate plus	26.7 (8.2)	50.0 (8.5)		
**Effectiveness outcomes: post partum**				
Total No.	67	69	NA	NA
Illicit opioid–negative urine samples, % UDS^b^^,^^g^	60.2 (4.2)	59.5 (4.1)	0.65 (−12.72 to 14.02) pp	.45
Buprenorphine adherence, % days	69.3 (4.3)	75.5 (4.2)	−6.15 (−24.05 to 11.75) pp	.30
Drug and alcohol abstinence, % UDS^d^	34.7 (3.3)	35.0 (3.2)	−1.34 (−15.08 to 12.39) pp	.77
Opioid Craving Scale^h^	1.5 (0.2)	1.7 (0.2)	0.06 (−0.50 to 0.62)	.82
Short Opiate Withdrawal Scale^h^	4.1 (0.5)	5.3 (0.5)	−1.81 (−3.87 to 0.25)	.009
**Significant safety outcomes**				
Pain management receipt, %				
During postpartum hospital stay^i^	85.7 (4.4)	98.5 (1.5)	RR, 0.87 (0.43 to 1.00)	.03
Opioid medication	30.2 (5.8)	50.0 (6.2)	RR, 0.60 (0.34 to 0.95)	.02
Postpartum phase				
HADS Anxiety total score^h^	5.8 (0.4)	6.8 (0.4)	−1.05 (−2.07 to −0.02)	.04

Abbreviations: HADS, Hospital Anxiety and Depression Scale; NA, not applicable; OR, odds ratio; pp, percentage point; RR, relative risk; UDS, urine drug screen.

^a^ Effect size denotes difference in estimated means; CI matches the α for statistical testing (eg, 95% CI for α = .05; 98.33% for α = .0167; 99.67% CI for α = .0033).

^b^ Data were truncated after medication disruption for impacted participants in the extended-release buprenorphine group.

^c^ Overall, 89.3% of urine samples were collected during pregnancy, 93.0% in the extended-release and 85.7% in the sublingual group; missing results were imputed as positive for illicit opioids.

^d^ Excluding cotinine.

^e^ Data available for 67 participants in the extended-release group and 70 in the sublingual group.

^f^ Data available for 30 participants in the extended-release group and 36 in the sublingual group.

^g^ Overall, 71.6% of urine samples were collected post partum, 69.5% in the extended-release and 73.4% in the sublingual group; missing results were imputed as positive for illicit opioids.

^h^ Data available for 66 participants in the extended-release group and 67 in the sublingual group.

^i^ Data available for 63 participants in the extended-release group and 66 in the sublingual group.

**Table 3.** Infant Effectiveness and Significant Safety Outcomes as a Function of Study Treatment

Outcome	Infants by study treatment group, mean (SE)		Effect size (CI)^a^	*P* value
	Extended release (n = 66)	Sublingual (n = 69)		
Key secondary outcomes				
Infants treated with opioids, %^b^	30.2 (5.8)	26.5 (5.4)	RR, 1.14 (0.54 to 1.99)	.64
Neonatal opioid treatment time, d^c^	10.9 (2.2)	14.8 (3.0)	RR, 0.73 (0.36 to 1.51)	.28
Secondary outcomes				
Total amount of morphine for NOWS, mg^d^	10.0 (3.7)	10.4 (3.2)	−0.90 (−18.27 to 16.47)	.86
Hospital length of stay, d^e^	8.5 (0.9)	9.5 (1.0)	RR, 0.92 (0.59 to 1.42)	.54
Finnegan Modified peak score^f^	10.4 (0.8)	10.0 (0.9)	0.79 (−2.50 to 4.08)	.45
Maternal custody, %^g^	90.9 (3.5)	89.7 (3.7)	RR, 1.01 (0.71 to 1.10)	.81
Case with child protective services, %^h^	32.8 (5.9)	38.8 (6.0)	RR, 0.85 (0.36 to 1.53)	.48
12-mo Developmental issues, %^i^	17.3 (5.2)	15.4 (5.0)	RR, 1.13 (0.26 to 3.31)	.79
Infant safety outcomes				
Head circumference, cm^j^	34.0 (0.2)	33.4 (0.2)	0.63 (−0.00 to 1.26)	.049

Abbreviations: NOWS, neonatal opioid withdrawal syndrome; RR, relative risk.

^a^ Effect size denotes difference in estimated means. The CI matches the α for statistical testing (eg, 95% CI for α = .05; 98.33% for α = .0167; 99.67% CI for α = .0033).

^b^ Data were truncated after medication disruption for impacted extended-release buprenorphine participants.

^c^ For infants receiving opioid treatment (19 in the extended-release group and 18 in the sublingual group).

^d^ Data available for 11 infants in the extended-release group and 13 in the sublingual group.

^e^ Data available for 65 infants in the extended-release group and 67 in the sublingual group.

^f^ Includes neonates scored using the Finnegan Modified version (22 in the extended-release group and 23 in the sublingual group).

^g^ Data available for 66 infants in the extended-release group and 68 in the sublingual group.

^h^ Data available for 64 infants in the extended-release group and 67 in the sublingual group.

^i^ Assessed among infants with a 12-month Ages and Stages Questionnaire, Third Edition (52 in the extended-release group and 52 in the sublingual group).

^j^ Data available for 61 infants in the extended-release group and 67 in the sublingual group).

### Safety Outcomes

Serious and nonserious event summaries (Table 4) showed that fewer participants in the extended-release buprenorphine group had serious AEs during pregnancy (6 [8.7%] vs 19 [26.8%]; *P* = .007) and post partum (4 [6.0%] vs 13 [18.6%]; *P* = .04). Only 1 serious medication-related event occurred (sublingual buprenorphine group; hospitalization after return to opioid use). All 135 infants with a medical record safety outcome had a live birth and were discharged alive. Infant serious events mainly involved hospitalizations, occurring for a variety of reasons (eg, pneumonia, poor weight gain, and others) (eTable 3 in Supplement 2) and congenital anomalies (eTable 4 in Supplement 2). With the exception of 1 hospitalization (infant in the sublingual buprenorphine group for NOWS), no hospitalization or congenital anomaly was related to study drug. One infant death (sublingual buprenorphine group, unrelated to medication) occurred during a cosleeping event. Maternal nonserious adverse events did not differ between groups, but during pregnancy, these were more frequently deemed medication-related in the extended-release than sublingual buprenorphine group (18 [26.1%] vs 5 [7.0%]; *P* = .003). These medication-related AEs were rated as mild or moderate, with more gastrointestinal issues in the extended-release buprenorphine group (eTable 5 in Supplement 2). eTable 6 in Supplement 2 provides AEs by MedDRA System Organ Class. Injection site reactions were generally mild (eTables 7 and 8 in Supplement 2). Other maternal safety results in Table 2 show fewer extended-release buprenorphine participants received any pain medication (mean [SE], 85.7% [4.4] vs 98.5% [1.5]; *P* = .03) and opioids specifically (30.2% [5.8] vs 50.0% [6.2]; *P* = .02) during postdelivery hospitalization. Extended-release buprenorphine participants had significantly lower postpartum anxiety scores. All other maternal safety analyses were not significant (eTable 9 in Supplement 2). Nonfatal overdoses occurred in 1 participant in the sublingual buprenorphine group during pregnancy, and 3 participants in the extended-release buprenorphine group post partum; data were insufficient for statistical analysis. The participant in the sublingual buprenorphine group reported inconsistent medication use, and all participants in the extended-release buprenorphine group stopped the medication for several months before the overdoses. Other infant safety results (Table 3) significantly differed only in mean (SE) infant birth head circumference, which was larger among infants exposed to extended-release buprenorphine (34.0 [0.2] vs 33.4 [0.2] cm; *P* = .049); the non–statistically significant results are provided in eTable 10 in Supplement 2.

**Table 4.** Serious and Nonserious Adverse Events Occurring During the Study^a^

Adverse event	Participants by treatment group, No. (%)					
	Maternal (pregnancy)		Maternal (post partum)		Infant	
	Extended release (n = 69)	Sublingual (n = 71)	Extended release (n = 67)	Sublingual (n = 70)	Extended release (n = 67)	Sublingual (n = 70)
Participants with ≥1 serious event						
Any serious event	6 (8.7)^b^	19 (26.8)^b^	4 (6.0)^c^	13 (18.6)^c^	9 (13.4)	11 (15.7)
Medication related	0	1 (1.4)	0	0	0	1 (1.4)
Congenital anomaly or birth defect	0	0	0	0	4 (6.0)	4 (5.7)
Persistent or significant disability or incapacity	0	0	0	0	0	0
Death	0	0	0	0	0	1 (1.4)
Initial or prolonged hospitalization	6 (8.7)^b^	19 (26.8)^b^	4 (6.0)	11 (15.7)	6 (9.0)	6 (8.6)
Life threatening	0	0	0	2 (2.9)	0	0
Important medical event	0	0	0	1 (1.4)	0	0
Participants with ≥1 nonserious TEAE^d^						
Any nonserious TEAE	44 (63.8)	47 (66.2)	37 (55.2)	45 (64.3)	NA	NA
Medication related	18 (26.1)^b^	5 (7.0)^b^	2 (3.0)	3 (4.3)	NA	NA
Organized by severity^e^						
Mild	21 (47.7)	16 (34.0)	13 (35.1)	11 (24.4)	NA	NA
Moderate	17 (38.6)	17 (36.2)	18 (48.6)	23 (51.1)	NA	NA
Severe	6 (13.6)	14 (29.8)	6 (16.2)	11 (24.4)	NA	NA

Abbreviations: NA, not applicable; TEAE, treatment-emergent adverse event.

^a^ Maternal adverse and serious adverse events occurred in the safety window, which began at the first dose date and ended either 7 days after the last sublingual buprenorphine dose, 7 days after the last weekly extended-release dose, or 28 days after the last monthly extended-release dose. An α of .05 was selected for each test of significance.

^b^ *P* < .01.

^c^ *P* < .05.

^d^ TEAEs were not tracked in infants.

^e^ Proportion based on participants with a nonserious event.

## Discussion

To our knowledge, this is the first randomized clinical trial to test extended-release buprenorphine for OUD in pregnancy. Participants receiving weekly extended-release buprenorphine had a significantly higher illicit opioid abstinence rate during pregnancy compared with those receiving sublingual buprenorphine. Other maternal and infant outcomes were generally similar between groups, save for fewer serious AEs during pregnancy and post partum, less maternal opioid pain medication receipt during postdelivery hospitalization, lower postpartum anxiety scores, and higher medication-related AEs during pregnancy in the extended-release buprenorphine group compared with sublingual group. Larger birth head circumference in extended-release buprenorphine–exposed neonates, relative to those exposed to sublingual buprenorphine, was the only statistically significant infant safety finding.

Illicit opioid abstinence was significantly greater among those receiving extended-release buprenorphine (82.5%) than sublingual buprenorphine (72.6%) during pregnancy, which was higher than the 65% reported in prior research.^28^ Opioid craving and withdrawal were minimal, and buprenorphine adherence was high (approximately 85%) in both groups during pregnancy. The superior illicit opioid abstinence observed in the extended-release buprenorphine group potentially is related to its pharmacokinetic advantages (eg, no daily peak-trough).

Only 28% of infants received opioid treatment for NOWS, lower than the 39% to 48% reported in the literature.^29,30,31^ This may be due to the majority of participants having adequate or better prenatal care (Kotelchuck index^21^), and already being established on buprenorphine at the time of randomization. Some of the hospitals used the Eat, Sleep, Console method for scoring NOWS, which has been shown to result in a lower proportion of infants being started on opioids.^32^ However, there was not a significant difference in the proportion receiving opioid treatment for the 30 infants scored with this system vs the 105 infants scored with another or unknown system (23.3% vs 29.1%; *P* = .65 [data not shown]). Postpartum, illicit opioid abstinence rates dropped and were similar in both groups (60.2% vs 59.5%). Postpartum individuals face additional challenges, including the demands of infant care for those with infant custody (90% of the sample)^1^ and, for those with an open CPS case (36% of the sample), potential concern about continuing treatment^33^; the high rate of open CPS cases is consistent with the finding that opioid-medication treatment is associated with an increased risk of CPS referral often due to state laws requiring reporting of opioid-medication exposure.^34^ The combination of high rates of custody and open CPS cases may account for the observed increase in medication discontinuation postpartum (35%) relative to during pregnancy (10%). While not significant, postpartum buprenorphine adherence was higher in the sublingual buprenorphine (75.5%) than extended-release buprenorphine (69.3%) group. Breastfeeding participants who received extended-release buprenorphine continued weekly injections (81%), whereas sublingual buprenorphine participants received buprenorphine covering a longer period, which may have been more convenient. Recent animal research suggests that N-methyl-2-pyrrolidone, an excipient in monthly extended-release buprenorphine, does not have adverse fetal-infant effects^8^ and Food and Drug Administration labeling was updated to remove the previously stated potential effect; thus, the monthly formulation might be considered for use when breastfeeding.^35^

Importantly, participants in the extended-release group had fewer serious AEs during pregnancy and post partum. However, they had more nonserious AEs during pregnancy rated as medication related. Notably, these were mostly mild, and none were severe. Research has found that attributing AEs to study medication can be unreliable, particularly for gastrointestinal AEs (ie, constipation, nausea, and vomiting),^36^ and AEs are overestimated in open-label trials.^37^ The absence of treatment group differences in AE rates (ie, regardless of medication relatedness) in the present trial, along with gastrointestinal AEs attributed to extended-release buprenorphine (n = 14) at double the rate for sublingual buprenorphine (n = 6) suggests a potential bias in attributing AEs to the more novel medication. Fewer participants in the extended-release group received opioid pain medication after delivery, and they had modestly lower postpartum anxiety scores. Sublingual buprenorphine has been shown to be efficacious for pain^38^ and to have anxiolytic effects.^39^ These effects might be more pronounced for extended-release buprenorphine, which provides more stable buprenorphine levels and higher exposure. Extended-release injection site reactions were primarily mild and moderate, consistent with past research.^40^ Overdose events were rare in both groups and associated with no longer taking the medication or inconsistent medication use.

The only significant infant safety difference between groups was a larger head circumference at birth in the extended-release buprenorphine group, although both groups’ averages were within normal range.^41^ This finding’s clinical significance is unclear, but it is reassuring given the higher exposure from extended-release buprenorphine.^7^ The lack of adverse safety outcomes in extended-release buprenorphine–exposed infants, relative to sublingual buprenorphine–exposed infants, is consistent with research finding that higher doses of buprenorphine during pregnancy are not associated with adverse infant outcomes.^42^ The observed lack of significant treatment group differences in infant sedation is consistent with a recent study of extended-release buprenorphine, which found that, similar to the results from studies of sublingual buprenorphine, buprenorphine plasma concentrations in breastfed infants were below the lower limit of quantification.^35^ Many of the other maternal and infant safety outcomes are consistent with normal ranges and rates in the general US population (ie, Apgar scores,^43^ rates of primary cesarean delivery,^44^ and medical complications^45^) or with findings from buprenorphine-exposed neonates (ie, length and weight,^46^ rates of preterm birth,^30^ abnormal conditions,^47^ neonatal intensive care unit admissions^30^). The rate of approximately 16% of infants scoring below the cutoff for a developmental area is consistent with the 12% to 16% prevalence of developmental disabilities in US children.^24^

### Strengths and Limitations

This trial’s strengths include a randomized design, 13 geographically diverse sites, comprehensive effectiveness and safety assessments, a sample with polysubstance use, and high medication adherence and study completion rates.

It also has limitations. Some outcomes may have been underpowered despite adequate power for the primary measure; outcomes relying on medical records had more missing data and potential quality issues, including likely variation in Modified Finnegan scoring across hospitals. The sample was clinically stable relative to many pregnant persons with OUD, as most participants were receiving sublingual buprenorphine treatment prior to randomization; thus, it is not clear whether the findings will generalize to a more unstable OUD population or to patients being initiated on buprenorphine. However, the sample did have high rates of current cannabis and stimulant use disorders and nicotine use, which is common among patients receiving buprenorphine treatment. Finally, the sample was largely White and non-Hispanic, and it is unclear whether the findings will generalize to a more ethnically and racially diverse patient population.^48^

## Conclusions

In this trial, pregnant adults with OUD who were randomized to receive extended-release buprenorphine had higher rates of illicit opioid abstinence during pregnancy than those randomized to receive sublingual buprenorphine. However, there was not between-group difference in the postpartum period. These results support the use of weekly extended-release buprenorphine in pregnant individuals with OUD.

## Notes

Supplement 1.
Trial Protocol and Statistical Analysis Plan

Supplement 2. **eTable 1.** Summary of medication dosing for participants impacted by medication disruption **eTable 2.** Study medication dosing as a function of phase and treatment group **eTable 3.** Listing of initial or prolonged hospitalizations in infants as a function of treatment group **eTable 4.** Listing of congenital anomalies in infants as a function of treatment group **eTable 5.** Study medication-related adverse events during pregnancy as a function of treatment group **eTable 6.** Serious and nonserious adverse events occurring during the study by MedDRA System Organ Class **eTable 7.** Summary of study injection site examinations **eTable 8.** Injection site reactions by severity and time to resolution **eTable 9.** Other maternal safety outcomes as a function of phase and treatment group **eTable 10.** Other infant safety outcomes as a function of phase and treatment group

Supplement 3.
Data Sharing Statement
