# Supplementary material for: Extended-Release vs Sublingual Buprenorphine in Pregnancy Through 12 Months Post Partum: A Randomized Clinical Trial
Source: JAMA Intern Med. 2026 Mar 16;186(5):533–43. doi: 10.1001/jamainternmed.2026.0057 (PMC12993732; doi:10.1001/jamainternmed.2026.0057)
Supplement: Supplement 1. — Trial Protocol and Statistical Analysis Plan [file jamainternmed-e260057-s001.pdf]

NIDA CTN Protocol 0080

Medication treatment for Opioid use disorder in expectant Mothers (MOMs): a pragmatic randomized trial comparing extended-release and daily buprenorphine formulations

Lead Investigator: T. Winhusen, PhD
Sub-Investigator: Michelle Lofwall, MD
Protocol Manager: Frankie Kropp, MS

Funded by: National Institute on Drug Abuse (NIDA)

May 21, 2021

Version 8.0

CONFIDENTIAL

Protocol Development Team

Shelly Greenfield, MD, MPH	Harvard University
Connie Guille, MD	The Medical University of South Carolina
Hendree Jones, PhD	University of North Carolina
Michelle Lofwall, MD	University of Kentucky
Stephanie Merhar, MD, MS	Cincinnati Children's Hospital
Sean M. Murphy, PhD	Weill Cornell Medicine
Edward Nunes, MD	Columbia University
John Rotrosen, MD	New York University
Davida M. Schiff, MD	Massachusetts General Hospital
Michael Silverstein, MD, MPH	Boston University
Mishka Terplan, MD, MPH	Virginia Commonwealth University
Elisha M Wachman, MD	Boston Medical Center
Scott Wexelblatt, MD	Cincinnati Children's Hospital
Christine Wilder, MD	University of Cincinnati

Emmes

Dikla Blumberg, PhD	Abigail G Matthews, PhD
Mitra Lewis, MS	Lauren Yesko
Eve Jelstrom, CRNA, MBA	Kelsey Krause

NIDA CCTN

Carmen Rosa, MS	CCTN Scientific Officer

TABLE OF CONTENTS

1.0 LIST OF ABBREVIATIONS 7
2.0 STUDY SCHEMA 9
3.0 STUDY SYNOPSIS 10
3.1 STUDY OBJECTIVES 10
3.2 STUDY DESIGN 10
3.3 STUDY POPULATION 10
3.4 TREATMENTS 10
3.5 ASSESSMENTS 11
3.6 PRIMARY ANALYSIS 11
4.0 BACKGROUND AND RATIONALE 12
4.1 BACKGROUND 12
4.1.1 Introduction 12
4.1.2 Treatment Adherence and Retention 12
4.1.3 Daily Peak-Trough Effects 12
4.1.4 Potential Benefits of Extended Release (XR) formulations 13
4.1.5 Overview of Protocol Objectives 13
4.2 RATIONALE FOR STUDY MEDICATIONS 14
4.2.1 CAM2038 vs. Sublocade™ 14
4.2.2 BUP-SL vs. Methadone as the Comparator 15
4.2.3 BUP-SL and BUP/NX-SL 15
4.3 RATIONALE FOR CLINICAL TRIAL DESIGN ELEMENTS 16
4.3.1 Open-Label vs. Double Dummy Design 16
4.3.2 Medication Check Visits vs. Research Visits 16
4.3.3 Non-inferiority Primary Analysis 16
4.3.4 Standardization of NOWS Scoring and Treatment 16
4.3.5 Potential Ancillary Studies 17
4.4 CONCEPTUAL MODEL 17
4.4.1 Mother Outcomes 18
4.4.2 Infant Outcomes 18
4.5 HEALTH ECONOMICS 20
4.6 INFANT NEURODEVELOPMENT 21
5.0 STUDY OBJECTIVES 22
5.1 PRIMARY OBJECTIVE 22
5.2 SECONDARY OBJECTIVE 22
5.3 TERTIARY OBJECTIVE 22
5.4 QUATERNARY OBJECTIVE 22
6.0 STUDY DESIGN 23
6.1 OVERVIEW OF STUDY DESIGN 23
6.2 NUMBER OF SITES AND PARTICIPANTS 23
6.3 STUDY DURATION 23
6.4 SITE AND PARTICIPANT SELECTION 23
6.4.1 Site Selection 23
6.4.1.1 Site Characteristics 24
6.4.2 Participant Selection 24
6.4.2.1 Inclusion Criteria 24
6.4.2.2 Exclusion Criteria 25
6.4.2.3 Rationale for Eligibility Criteria 26

7.0 STUDY MEASURES 27

7.1 Key Outcome Measures of the Primary Objective 27

7.1.1 Primary Outcome - Illicit Opioid Abstinence During Pregnancy 27

7.1.2 Key Secondary Outcomes (Primary Objective) 27

7.1.2.1 NOWS Severity 27

7.1.2.2 Postpartum Illicit Opioid Abstinence 28

7.2 Secondary Outcome Measures (Primary Objective) 28

7.2.1 Mother Secondary Outcomes 28

7.2.1.1 BUP Medication Adherence 28

7.2.1.2 Drug and Alcohol Abstinence 28

7.2.1.3 The Opioid Craving Scale 28

7.2.1.4 Adequacy of Prenatal Care Utilization 29

7.2.1.5 The Short Opiate Withdrawal Scale (SOWS)-Gossop 29

7.2.2 Infant Secondary Outcomes 29

7.2.2.1 Other NOWS-related Outcomes 29

7.2.2.2 Discharge Outcomes 29

7.2.2.3 Infant Development 29

7.3 Safety Measures of the Primary Objective 30

7.3.1 Adverse Events (AEs) 30

7.3.2 The Hospital Anxiety and Depression Scale (HADS) 30

7.3.3 Prior/Concomitant Medications 30

7.3.4 Fetal Outcomes 30

7.3.5 Maternal Delivery Outcomes 30

7.3.6 Birth/Neonatal Outcomes 31

7.3.7 Injection Site Examination 31

7.3.8 Opioid Overdose Tracking 31

7.3.9 Infant Sedation 31

7.4 Covariates 31

7.4.1 Covariates for Mother Outcomes 32

7.4.2 Covariates for Infant Outcomes 32

7.5 Health Economic Measures of the Tertiary Objective 33

7.5.1 Healthcare Service Utilization 33

7.5.2 Health-related quality of life 33

7.5.3 Abstinent Year 34

7.6 Other Measures 34

7.6.1 Screening Assessments 34

7.6.2 Sample Characteristics 35

7.6.3 General Measures 36

7.7 Administrative Forms 36

7.8 Conceptual Model Assessments of the Secondary Objective 37

7.9 Infant Neurodevelopmental Outcomes of the Quaternary Objective 39

8.0 STUDY PROCEDURES 40

8.1 Study Overview 40

8.2 Participant Recruitment, Pre-screening, and Consent 40

8.3 Screening/Baseline 41

8.4 Randomization Plan 41

8.5 Active Treatment Phase 45

8.6 Participant Reimbursement 45

8.7 Medication and Trial Discontinuation 46

8.7.1 Medication Discontinuation 46

8.7.2 Trial Discontinuation 46

8.8 Access to Treatment After Study Completion 46

8.9 Conceptual Model Assessments (CMA) Sub-study 46

8.9.1 Overview 46

8.9.2 Recruitment and Consent 47

8.9.3 Visits for the CMA Sub-study ... 47
8.10 Infant Neurodevelopmental Outcomes (INO) Sub-study ... 47
8.10.1 Overview ... 47
8.10.2 Recruitment ... 48
8.10.3 INO Visits ... 48

9.0 STUDY MEDICATIONS ... 49

9.1 BUP-XR (CAM2038) ... 49
9.2 BUP-SL ... 49
9.3 Dispensing Study Medication ... 49
9.3.1 Induction ... 50
9.4 Missed Doses ... 51
9.5 Clinical non-response ... 51
9.6 Study Medication Management/Drug Accountability ... 51
9.6.1 Storage ... 51
9.6.2 Documentation ... 51
9.6.3 Used/Unused Medication ... 52
9.6.4 Lost Medication ... 52
9.6.5 Medication Packaging ... 52
9.7 Concomitant Medications ... 52

10.0 ANALYTICAL PLAN ... 53

10.1 Statistical Hypotheses for Primary Objective ... 53
10.1.1 Key Hypotheses ... 53
10.1.2 Secondary Hypotheses ... 53
10.2 Intent-to-Treat Participant Population ... 53
10.3 Analysis Plan ... 54
10.3.1 Non-Inferiority Margin ... 54
10.3.2 Key Outcomes ... 54
10.3.3 Secondary Outcomes ... 55
10.3.4 Safety Analyses ... 56
10.3.4.1 Adverse Events ... 56
10.3.4.2 The Hospital Anxiety and Depression Scale (HADS) ... 56
10.3.4.3 Fetus/Delivery-Related Outcomes ... 56
10.3.4.4 Birth/Neonatal Outcomes ... 56
10.3.5 Missing Data ... 56
10.4 Sample Size Analysis ... 57
10.4.1 Parameter Selection for Calculations ... 57
10.4.2 Results ... 57
10.5 Descriptive Statistics ... 57
10.6 Interim Analyses ... 58
10.7 Minority/Sex Analyses ... 58
10.8 Conceptual Model Analyses ... 58
10.9 Health Economic Analytic Plan ... 58
10.9.1 Overview ... 58
10.9.2 Intervention Costs ... 59
10.9.3 Healthcare Service Utilization ... 59
10.9.4 QALYs ... 60
10.9.5 ICERs and Cost-Effectiveness ... 60
10.9.6 Missing Health Economic Data ... 60
10.9.7 Sensitivity Analyses ... 60
10.10 Neurodevelopmental Outcomes Analysis ... 60
10.11 Post-hoc Analyses ... 61

11.0 REGULATORY COMPLIANCE, REPORTING, AND MONITORING 62
11.1 Regulatory Compliance 62
11.2 Statement of Compliance 62
11.3 Institutional Review Board Approval 62
11.4 Regulatory Files 62
11.5 Research Advisory Panel of California (California sites only) 63
11.6 Informed Consent 63
11.7 Participant and Data Confidentiality 64
11.7.1 Health Insurance Portability and Accountability Act (HIPAA) 65
11.8 Investigator Assurances 65
11.8.1 Financial Disclosure/Conflict of Interest 65
11.8.2 DEA Registration 65
11.8.3 Investigational New Drug (IND) Requirements 65
11.9 Quality Assurance Monitoring 65
11.10 Prisoner Certification 66
11.11 Protections for Pregnant Women, Fetuses, and Neonates 66
11.12 Records Retention and Requirements 67
11.13 Reporting to Sponsor 67
11.14 Audits 67
11.15 Study Documentation 67
11.16 Protocol Deviations 67
11.17 Safety Monitoring 68
11.17.1 Data and Safety Monitoring Board (DSMB) 68
11.17.2 Adverse Events (AEs) 68
11.17.3 Medical Monitor 70
11.17.4 Known Potential Toxicities of Study Medication/Intervention 71
11.18 Training Requirements 71
12.0 DATA MANAGEMENT AND PROCEDURES 72
12.1 Design and Development 72
12.1.1 Site Responsibilities 72
12.1.2 Data Center Responsibilities 72
12.1.3 Data Collection 72
12.2 Data Acquisition and Entry 72
12.3 Data Editing 72
12.4 Database Transfer/Lock 73
12.5 Data Sharing 73
12.6 Data Training 73
12.7 Data QA 73
13.0 PUBLIC ACCESS AND DATA SHARING PLAN 74
14.0 PROTOCOL SIGNATURE PAGE 75
15.0 REFERENCES 76
16.0 APPENDIX A: ADVERSE EVENT REPORTING AND PROCEDURES 87
17.0 APPENDIX B: DATA AND SAFETY MONITORING PLAN 92
17.1 Brief Study Overview 92
17.1.1 Protocol Description 92
17.1.2 Key outcome measures 92
17.1.3 Inclusion/Exclusion Criteria: 92
17.1.4 Sample Size 94
17.1.5 Overview of Protocol Monitoring 94
17.2 Oversight of Clinical Responsibilities 94
17.2.1 Site Principal Investigator (PI) 94

17.2.2 CCC Medical Monitor 95
17.2.3 Mandatory Regulatory Reporting in IND Trials 95
17.3 DATA AND SAFETY MONITORING BOARD (DSMB) 95
17.4 QUALITY ASSURANCE (QA) MONITORING 95
17.5 MANAGEMENT OF RISKS TO PARTICIPANTS 96
17.5.1 Confidentiality 96
17.5.2 Information That Meets Reporting Requirements 96
17.5.3 Participant Protection 96
17.6 PREGNANCY AND PREGNANCY OUTCOMES 96
17.7 STUDY SPECIFIC RISKS 97
17.8 COLLECTION AND MANAGEMENT OF AES AND SAES 97
17.9 DATA OVERSIGHT 98
17.9.1 Data and Statistics Center Responsibility 98
17.9.2 Data Collection and Entry 98
17.9.3 Data Monitoring, Cleaning, and Editing 98
17.9.4 Database Lock and Transfer 99

18.0 APPENDIX C: REGULATORY 100

18.1 SINGLE IRB 100
18.2 STUDY DISCONTINUATION 100

1.0 LIST OF ABBREVIATIONS

Abbreviation	Definition
ACOG	American College of Obstetricians and Gynecologists
AE	Adverse Event
APNCU	Adequacy of prenatal care utilization
ASQ-3	Ages and Stages Questionnaire, Third Edition
Bayley™-4	Bayley Scales of Infant and Toddler Development™, Fourth Edition
BORN	Better Outcomes Through Research for Newborns
BPP	Biophysical profile
BUP-SL	Sublingual buprenorphine; refers to both with and without naloxone
BUP-XR	Extended-release buprenorphine
CBCL	Child Behavior Checklist
CCC	Clinical Coordinating Center
CCTN	Center for Clinical Trials Network
CDC	Centers for Disease Control and Prevention
CMA	Conceptual model assessment
C_{max}	Peak BUP plasma concentration
C_{min}	Trough BUP plasma concentration before the subsequent dose
CoC	Certificate of Confidentiality
CRF	Case report form
CTN	Clinical Trials Network
DEA	Drug Enforcement Agency
DSC	Data and Statistics Center
DSMB	Data and Safety Monitoring Board
EDC	Electronic Data Capture
EGA	Estimated gestational age
EHR	Electronic Health Record
EOT	End of Treatment
FDA	Food and Drug Administration
FHRV	Fetal heart rate variability
GCP	Good Clinical Practice
GDO	Glycerol dioleate
HADS	Hospital Anxiety and Depression Scale
HHS	Department of Health and Human Services
HIPAA	Health Insurance Portability and Accountability Act
HRQoL	Health-related quality-of-life
ICER	Incremental cost-effectiveness ratio
IND	Investigational New Drug
INO	Infant Neurodevelopmental Outcomes
IRB	Institutional review board
ITT	Intent-to-Treat
IUP	Intrauterine Pregnancy
LI	Lead Investigator
LOS	Length of stay

Abbreviation	Definition
MAT	Medication-assisted treatment
MC	Medical Clinician
NAS	Neonatal Abstinence Syndrome
NIDA	National Institute on Drug Abuse
NMOS	Non-study Medical and Other Services
NMP	N-methyl-2-pyrrolidone
NOWS	Neonatal Opioid Withdrawal Syndrome
NST	Non-stress test
OHRP	Office for Human Research Protections
OUD	Opioid use disorder
PAASA	Pregnancy and Addiction Services Assessment
PI	Principal Investigator
PK	Pharmacokinetic
PRISM	Psychiatric Research Interview for Substance and Mental Disorders
PROMIS	Patient-Reported Outcomes Measurement Information System
PROPr	PROMIS-Preference
QA	Quality Assurance
QALYs	Quality-adjusted life-years
RA	Research assistant
RAP-C	Research Advisory Panel of California
RCT	Randomized controlled trial
SAE	Serious Adverse Event
SAMHSA	Substance Abuse and Mental Health Services Administration
SOP	Standard Operating Procedure
SOWS	Short Opiate Withdrawal Scale
SUD	Substance Use Disorder
TLFB	Timeline Followback
UC	University of Cincinnati
UDS	Urine drug screen

2.0 STUDY SCHEMA

Fig. 1: Study Schema

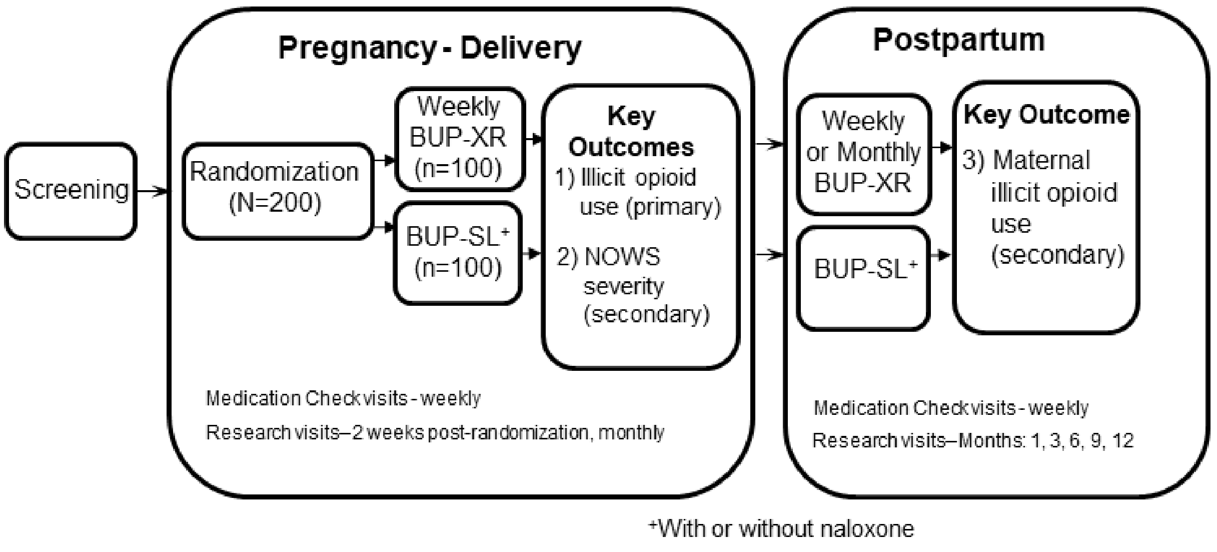

3.0 STUDY SYNOPSIS

3.1 Study Objectives

CTN-0080 includes four objectives:

- Primary Objective: To evaluate the impact of treating opioid use disorder (OUD) in pregnant women with extended-release (XR) buprenorphine (BUP), compared to sublingual (SL) BUP, on mother and infant outcomes. Hypothesized outcomes are that the BUP-XR, relative to the BUP-SL, group will:
    1) not have greater illicit opioid use during pregnancy (primary, non-inferiority);
    2) have lower infant neonatal opioid withdrawal syndrome (NOWS) severity (key secondary, superiority); and
    3) not have greater postpartum illicit opioid use (key secondary, non-inferiority).
- Secondary Objective: To test conceptual models of the mechanisms by which BUP-XR may improve mother-infant outcomes, relative to BUP-SL.
- Tertiary Objective: To determine the economic value of utilizing BUP-XR, relative to BUP-SL, to treat pregnant women.
- Quaternary Objective: To evaluate the impact of BUP-XR, relative to BUP-SL, on infant neurodevelopment.

3.2 Study Design

This is an intent-to-treat, two-arm, open-label, pragmatic randomized controlled trial. Eligible participants will be randomized in a 1:1 ratio to BUP-XR or BUP-SL, balancing on site, estimated gestational age (EGA) at time of randomization (6 weeks-18 weeks vs. 19 weeks-30 weeks), and whether they are on BUP-SL at the time of randomization (yes vs. no). Participants will be provided with study medication and attend weekly medication visits through 12 months postpartum. Participants will be invited to participate in the conceptual model assessment (CMA) sub-study, which will be used to evaluate the MOMs conceptual models. Infant caregivers will be invited to participate in the infant neurodevelopmental outcomes (INO) sub-study, which will include a 24-month child assessment. The INO data will be locked separately from the rest of the CTN-0080 database to allow CTN-0080 database lock following collection of the final (non-INO) CTN-0080 data point.

3.3 Study Population

Approximately 200 pregnant women, recruited from approximately 10 sites, will be randomized into the trial. Sites that provide BUP to pregnant women in an office-based setting, offer BUP treatment following delivery for ≥12 months, and admit enough potentially eligible women to meet the target randomization rate (1.25 per month) are eligible. The study population will include pregnant women who have an EGA of 6-30 weeks at randomization, and, in the judgment of the treating provider, are good candidates for BUP-maintenance treatment. All randomized participants will be encouraged to participate in the CMA and INO sub-studies.

3.4 Treatments

Participants randomized to BUP-XR will receive a weekly formulation of CAM2038 during pregnancy. During the 12-month postpartum phase, women who are breastfeeding will continue

receiving BUP-XR weekly while women who are not breastfeeding will receive monthly BUP-XR. Participants randomized to BUP-SL will receive buprenorphine, with or without naloxone, based on site preference, during pregnancy and the 12-month postpartum phase.

3.5 Assessments

The primary outcome is illicit opioid abstinence during pregnancy, assessed by urine drug screens (UDSs). Key secondary outcomes for the primary objective are infant NOWS severity assessed by total days of opioid treatment (derived from medical records), and mother postpartum illicit opioid abstinence assessed by UDSs. The CMA sub-study includes assessments of: 1) maternal trough BUP plasma concentrations at study weeks 3 and 5; 2) fetal non-stress test and biophysical profile at ~36 weeks EGA at maternal peak BUP plasma level; 3) maternal peak and trough BUP plasma concentrations at ~36 weeks EGA; and 4) cord and maternal plasma BUP/BUP-metabolite levels at delivery. The main economic outcome will be the incremental cost-effectiveness ratio (ICER). The main neurodevelopmental outcome of the INO sub-study will be the cognitive subscale of the Bayley Scales of Infant and Toddler Development™, Fourth Edition (Bayley™-4) when the child is approximately 24 months of age.

Study assessments will ideally occur at the clinic site; however, as needed these visits may occur in whole or in part via telemedicine, at other institutionally-affiliated clinical sites, or elsewhere in the community (including, but not limited to, home visits, visits at other non-affiliated clinical/laboratory sites, or other community sites affording appropriate safety and confidentiality) as permitted by the institution and other regulatory bodies. For the CMA and INO sub-studies, it is likely that some study procedures will occur outside the clinic at locations deemed most appropriate by the providers performing the study assessments (including, but not limited to, the delivery hospital, an external laboratory, or other clinical location).

3.6 Primary Analysis

A Type-I error rate of α=.025 will be used for the non-inferiority primary outcome analysis.

4.0 BACKGROUND AND RATIONALE

4.1 Background

4.1.1 Introduction

The growing opioid-use epidemic in the U.S. has been associated with a significant increase in the prevalence of pregnant women with opioid use disorder (OUD)[1-4] and neonatal abstinence syndrome (NAS)/neonatal opioid withdrawal syndrome (NOWS).[5, 6] NAS/NOWS is associated with adverse health effects for the infant[7-9] and with costly hospitalizations.[6] In 2012, the average length of stay (LOS) for NAS/NOWS was 16.9 days and the average cost was $66,700.[6] In 2013, about 4% of total neonatal intensive care unit days were attributed to infants with NAS/NOWS, compared to just 0.6% in 2004.[10] The American College of Obstetricians and Gynecologists (ACOG) Committee, together with the American Society of Addiction Medicine, published their recommendation in 2012 that opioid-dependent pregnant women should not be tapered off opioids and should be treated with methadone or, possibly, buprenorphine (BUP).[11] A more definitive recommendation for either methadone or BUP treatment was made by the World Health Organization in 2014.[12] This opinion has been maintained based on more recent literature reviews,[13] and the relative advantages/disadvantages of each treatment approach have been further delineated. Relative to methadone, BUP offers the advantages of greater convenience for pregnant women and lower NAS/NOWS severity in their infants.[14] Some disadvantages of BUP include increased risk of diversion,[15] poorer adherence,[15] greater treatment dropout,[16-18] and daily peak-trough effects.[19] While applicable to individuals with OUD in general, these disadvantages may be more pronounced in pregnant women.

4.1.2 Treatment Adherence and Retention

Pregnant women with OUD face a number of challenges, including psychosocial stressors and psychiatric co-morbidity that may serve to reduce medication adherence.[20] In addition, because the most common concerns of BUP-maintained pregnant women are the health of their unborn infants and the potential for NAS/NOWS,[21] expecting 100% adherence to daily self-administration of a medication that a woman worries may harm her unborn infant may be unrealistic. While the reasons are not entirely clear, treatment retention in BUP-maintained pregnant women is problematic, with retention rates estimated to be 57.7% compared to retention rates of 78.1% for methadone treatment.[14] In SAMHSA's 2018 clinical guidance,[13] it was noted that the postpartum period is a time when women are particularly vulnerable for relapse and an increased risk of overdose during the postpartum phase has been documented.[22] Hence, receiving effective treatment during the postpartum period is of critical importance. The limited research on postpartum retention in opioid maintenance treatment has found dropout rates of up to 36% by 3 months postpartum and 62% by 6 months postpartum.[23] The high dropout rates are perhaps not surprising given the demands that motherhood entails, which likely serve to make daily medication adherence and treatment attendance more difficult.

4.1.3 Daily Peak-Trough Effects

In individuals with OUD, the daily BUP-SL trough is problematic in that plasma levels may be insufficient for suppressing opioid withdrawal symptoms.[19] In pregnant women, both the BUP-SL peak <u>and</u> trough may be problematic. For the fetus, the BUP-SL peak is associated with adverse effects, including decreased fetal heart rate and heart rate variability.[24] Problems with BUP-SL trough are likely exacerbated in pregnant women, relative to non-pregnant individuals, since BUP is cleared more rapidly during pregnancy.[25, 26] Research suggests that BUP plasma concentrations ≥1 ng/mL are required to suppress opioid withdrawal symptoms[27] and, thus,

BUP-SL should be dosed to maintain individuals at ≥1 ng/mL throughout the dosing cycle.[19] Modeling studies suggest that plasma concentrations of 2-3 ng/mL are required for opioid blockade;[19, 28] however, analysis of a recent opioid blockade study[29] revealed that BUP plasma concentrations ≥1.25 ng/mL were sufficient for achieving complete opioid blockade.[30] Thus, BUP plasma concentrations ≥1 ng/mL should, theoretically, be sufficient for suppressing opioid withdrawal and, for the subset of patients who use illicit opioids, BUP plasma concentrations ≥1.25 ng/mL should be sufficient to block illicit opioid effects. The results of a recent pharmacokinetic (PK) study indicate that pregnant women treated with BUP-SL may have BUP plasma concentrations <1 ng/mL, and, thus, be at sub-therapeutic doses, for most of their dosing interval.[31] Utilizing higher BUP-SL doses[26] and more frequent dosing in pregnant women[31] have been recommended as potential solutions. However, higher doses may be problematic from the perspective of fetus-infant outcomes in that higher maternal BUP-SL doses may be associated with: 1) greater depression of fetal heart rate even at trough;[24] 2) lower birth weight and length;[32] and 3) greater NAS/NOWS severity.[32, 33] Using a more frequent dosing schedule may also be problematic in that greater than once daily dosing is associated with lower rates of medication adherence.[34, 35]

4.1.4 Potential Benefits of Extended Release (XR) formulations

Extended release (XR) formulations can address some of the disadvantages of BUP-SL including potential peak-trough issues, non-adherence[36, 37] and diversion.[36] Two new BUP-XR products (CAM2038 from Braeburn Pharmaceuticals and Sublocade™ from Indivior) have recently been reviewed by the FDA. Once approved, CAM2038 will be marketed as Brixadi; therefore, all instances of CAM2038 in this document also refer to Brixadi. Both products are subcutaneously injected and form a gel deposit under the skin that releases BUP at a steady rate for up to one month; the CAM2038 product is also available as a weekly formulation. The benefits of eliminating potential diversion go beyond the individual patient in that potential diversion is a barrier to prescribing for some BUP-waivered providers;[38] removal of this barrier thus has the potential to increase the availability of treatment. This is especially important for pregnant women with OUD for whom there is a sizeable treatment gap.[39-41] XR formulations also avert the daily peak-trough cycle of BUP-SL, which, as noted above, is particularly problematic in pregnant women. A BUP formulation that minimizes peak concentrations while also helping to ensure that trough levels are ≥1 ng/mL would be ideal for meeting the needs of both the mother and infant; PK data suggest that CAM2038 achieves this. Specifically, the 24 mg weekly CAM2038 formulation approximates 12-16 mg of daily BUP-SL (i.e., the typical target dose range for treating OUD in pregnant women[13]) while having both lower peak (4.97 ng/mL vs. 6.09 ng/mL) and higher trough (1.18 ng/mL vs. 0.85 ng/mL) levels.[42]

4.1.5 Overview of Protocol Objectives

The present study is a pragmatic multi-site, randomized controlled trial (RCT) with the primary objective of evaluating the impact of treating OUD in pregnant women with BUP-XR, relative to BUP-SL, on mother-infant outcomes. In addition to being the first trial to evaluate BUP-XR in pregnant women, it will be one of the few multi-site RCTs of opioid maintenance treatment conducted with a relatively large sample size of pregnant women. Indeed, only one such RCT has been conducted: the Maternal Opioid Treatment: Human Experimental Research (MOTHER) trial,[43] which included 175 participants. The MOTHER trial, which compared methadone and BUP-SL maintenance treatments, was a very important clinical trial with a number of strengths. However, as noted by the investigators, MOTHER was a tightly controlled efficacy study that maximized internal validity at the expense of external validity,[44] which limits the generalizability of the results to clinical practice. Given the dearth of evidence upon which to base clinical guidance documents,[13] the present trial is designed to protect internal validity

through the use of randomization but to otherwise favor external validity. As noted by Ford and Norrie,[45] pragmatic trials are typically not pragmatic on all dimensions. CTN-0080 follows the suggestion to utilize pragmatic features where feasible while still maintaining trial quality and the ability to answer the question of interest.[45] The rationales for key design decisions related to the primary objective are provided below. A secondary objective of MOMs is to contribute to the science of OUD treatment in pregnant women by testing conceptual models of the mechanisms by which BUP-XR may improve mother and infant outcomes relative to BUP-SL. The conceptual models are described in ***section 4.4***. A tertiary objective of MOMs is to conduct a health economic analysis comparing the costs and benefits of BUP-XR and BUP-SL; the rationale for which is described in ***section 4.5***. A quaternary objective is to evaluate the impact of BUP-XR, relative to BUP-SL, on infant neurodevelopment; the rationale for which is described in ***section 4.6***.

4.2 Rationale for Study Medications

4.2.1 CAM2038 vs. Sublocade™

Two BUP-XR products have been developed recently (CAM2038 from Braeburn Pharmaceuticals and Sublocade™ from Indivior), both of which are subcutaneously injected and form a gel deposit under the skin that releases BUP at a steady rate for up to one month; the CAM2038 product is also available as a weekly formulation. Sublocade™ received FDA approval in 2017. In 2018, the FDA determined that CAM2038 met all safety and efficacy standards necessary for approval but did not approve it for marketing due to exclusivity issues. The monthly formulations of both Sublocade™ and CAM2038 use N-methyl-2-pyrrolidone (NMP), a compound which animal studies suggest may have adverse fetal-infant effects, as an excipient.

The weekly CAM2038 product does not include NMP and, thus, was selected as the BUP-XR medication for MOMs. Specifically, the weekly CAM2038 product will be utilized while participants are pregnant or breastfeeding and the monthly formulation will be utilized during the postpartum phase for women who are not breastfeeding. The weekly CAM2038 product includes three excipients: 1) phosphatidylcholine; 2) glycerol dioleate (GDO); and 3) ethanol. The target weekly CAM2038 dose is 24 mg, but a minority of participants may require the highest dose (32 mg). The maximum potential exposure to the excipients for any participant/fetus would be for a pregnant woman randomizing into MOMs at 6 weeks EGA, delivering at 40 weeks, and receiving the maximum potential CAM2038 dose (32 mg of buprenorphine in 0.64 mL) every week. The maximum potential exposure to these excipients for any participant/breastfeeding infant postpartum would be for a participant receiving the 32 mg CAM2038 dose every week.

Phosphatidylcholine may offer health benefits during pregnancy[46] and choline supplements of 450 mg/day during pregnancy have been recommended by the Institute of Medicine[47] and the American Medical Association.[48] The maximum weekly dose of CAM2038 (32 mg) includes 0.26 grams of phosphatidylcholine; the total possible maximum exposure during pregnancy would be 8.84 grams, which is less than the 107.1 grams that would be consumed by taking the recommended supplement of 450 mg/day. The total maximum exposure postpartum would be 13.52 grams compared to 163.8 grams if the 450 mg/day supplement were continued through one year postpartum. The second excipient, GDO, is a diglyceride and naturally occurs in human plasma. Diacylglycerol, of which GDO is a significant component, is widely used in food products (e.g., mayonnaise, salad dressings, margarine, icing, etc.).[49] The maximum weekly dose of CAM2038 (32 mg) includes 0.26 grams of GDO; the total possible maximum exposure during pregnancy would be 8.84 grams, which equates to less than one tablespoon (i.e., the

total would be 0.69 tablespoons). The total maximum exposure postpartum would be 13.52 grams, which equates to approximately one tablespoon (i.e., the total would be 1.06 tablespoons). The third excipient, ethanol, when consumed in sufficient quantities by pregnant women can have a teratogenic effect.[50] The maximum weekly dose of CAM2038 (32 mg) contains .061 grams of ethanol; a standard alcoholic drink in the US contains 14 grams of ethanol.[51] The total maximum exposure to ethanol during pregnancy would be 2.074 grams (i.e., <15% of the ethanol in a single standard drink). Although no level of alcohol exposure is considered safe during pregnancy, the potential substantial benefit of extended-release buprenorphine, relative to sublingual buprenorphine, (e.g., superior PK profile, elimination of diversion potential, improved adherence) justifies any theoretical risk from this negligible amount of subcutaneously injected ethanol. While alcohol use is not encouraged in breastfeeding women, the CDC notes that moderate alcohol use (up to 1 standard drink per day) is not known to be harmful to the infant.[52] The maximum potential total postpartum exposure (i.e., assuming 52 weeks at the maximum weekly CAM2038 dose) would be 3.172 grams, which is <23% of the ethanol in a single standard drink; thus the risk-benefit ratio for postpartum use is also justified.

4.2.2 BUP-SL vs. Methadone as the Comparator

BUP-SL, as opposed to methadone, was selected as the comparator for MOMs to maximize the potential impact of the study findings on clinical practice. Methadone can only be dispensed by licensed opioid treatment programs, which are highly regulated and have relatively limited availability. BUP, by contrast, is more widely available since it can be prescribed in office-based practices by certified practitioners. For outpatient practitioners, the results of a trial comparing a medication they can prescribe for the treatment of OUD (BUP-XR) to one they cannot prescribe (methadone) is likely to have little impact on practice. By using BUP-SL as the comparator, MOMs has the potential to show that BUP-XR is not inferior, and perhaps superior, to BUP-SL and, thus, is a reasonable alternative to BUP-SL, thus expanding the available treatment options.

4.2.3 BUP-SL and BUP/NX-SL

Participants randomized to BUP-SL will receive buprenorphine, without (BUP-SL) or with naloxone (BUP/NX-SL). The primary rationale for allowing both medications is to avoid discouraging site/patient participation by requiring the use of a medication that is inconsistent with site preference. In addition, allowing both medications is consistent with the goal of maintaining a pragmatic design where feasible. The inclusion of both BUP-SL and BUP/NX-SL may also make a valuable contribution to the field. As noted by Nguyen and colleagues,[53] BUP-SL is more commonly used in pregnant women based on the principal of limiting fetal exposure to additional compounds and the potential for induced withdrawal if BUP/NX-SL is injected, but BUP-SL is more likely to be diverted and misused than BUP/NX-SL. The existing literature on the relative safety of utilizing BUP/NX-SL during pregnancy is limited to retrospective chart reviews,[53] which generally have found no evidence of worse outcomes with BUP/NX-SL[54, 55] with the exception of a recent study which found rates for prematurity and low birth weight that were higher than expected.[53] In addition to being retrospective, the study sample sizes have been limited (i.e., N=10,[56] N=30,[57] N=7,[54] N=26,[53] N=31[58]). It is anticipated that at least three of the approximately 10 sites will utilize BUP/NX-SL; hence, it is estimated that a minimum of 38 MOMs participants will be taking BUP/NX-SL. Exploratory analyses of safety information comparing participants taking BUP-SL to those taking BUP/NX-SL could make an important contribution to the field.

4.3 Rationale for Clinical Trial Design Elements

4.3.1 Open-Label vs. Double Dummy Design

As noted above, there is a dearth of evidence on which to base clinical guidance documents for the treatment of OUD in pregnant women.[13] Consistent with the CTN mission, this trial is designed to compare the effectiveness of interventions as they would be used in the real world. The present trial is, thus, designed to protect internal validity using randomization but to otherwise favor external validity. In addition to not representing clinical practice, the use of a double dummy design would artificially remove a key advantage of BUP-XR: avoiding daily self-administration; hence, MOMs is an open-label trial.

4.3.2 Medication Check Visits vs. Research Visits

In a pragmatic trial, research assessments/interactions that could impact outcomes and, thus, reduce the generalizability of the results to real world practice are minimized. However, this goal must be balanced with the need to closely monitor safety given that CTN-0080 is the first trial to evaluate the BUP-XR formulation in pregnant women. This balance will be achieved by including weekly Medication Check Visits, which include a minimal number of assessments and procedures (see ***Table 3***), while limiting more intensive data collection to Research Visits, which will occur less frequently (e.g., monthly during pregnancy, etc.; see ***Table 3***).

4.3.3 Non-inferiority Primary Analysis

As detailed in the conceptual model (***section 4.4***), BUP-XR may improve outcomes, relative to BUP-SL, due to its superior PK profile. However, to be consistent with the design of the CAM2038 Phase 3 trial,[59] CTN-0080 will utilize a non-inferiority design. A finding of non-inferiority would suggest that BUP-XR is a reasonable alternative to BUP-SL, thus expanding available treatment options. This is important because, at present, the number of BUP providers is insufficient to meet treatment needs (particularly in rural areas). A significant concern of clinicians who are unwilling to prescribe BUP-SL is the potential for diversion;[38] removal of this barrier would, thus, have the potential to increase the availability of treatment.

4.3.4 Standardization of NOWS Scoring and Treatment

The primary outcome measure for CTN-0080 is illicit opioid use during pregnancy. This, combined with the goal of utilizing pragmatic features where feasible, suggests that standardization of NOWS scoring/treatment at the delivery hospitals, which are not participating in the trial as study sites, should not be undertaken. However, the variability in delivery-hospital approach to NOWS could adversely impact the evaluation of treatment effects on NOWS severity, which is a key secondary measure. Hence, it was decided to decrease delivery-hospital variability. Based on a review of the literature, as well as consensus of the NOWS experts of the protocol development team, it was determined that participants are only eligible (see ***section 6.4.2***) if they plan to deliver at a hospital that meets all the following requirements:

1) has a written protocol for the management of NAS/NOWS since it has been shown that implementation of a standard protocol decreases length of opioid treatment days, infant LOS, and use of adjunctive drug therapy.[60, 61] Based on a survey of Better Outcomes Through Research for Newborns (BORN) hospitals,[62] it is estimated that 88% of potential delivery hospitals will have a written protocol for NAS/NOWS management.

2) offers rooming-in while infants are being observed for NAS/NOWS, since rooming-in is associated with decreased need for pharmacologic treatment for NAS/NOWS and

shorter LOS.[63] Based on the results of the BORN survey,[62] it is estimated that 73% of potential delivery hospitals will offer rooming-in.

3) does not send infants home on opioids for the treatment of NAS/NOWS. This is required since infant NOWS severity will be measured by total number of opioid treatment days as assessed by medical record; opioids provided at home would not be captured making the outcome inaccurate. Based on the results of the BORN survey,[62] it is estimated that 83% of potential delivery hospitals will not send infants home on opioids.

In addition, participants are only eligible to participate (see ***section 6.4.2***) if they plan to deliver at a hospital that has provided information about their approach to NAS/NOWS, so that important variables can be controlled for in the NOWS analyses (see ***section 7.4.2***).

4.3.5 Potential Ancillary Studies

CTN-0080 has the potential to serve as a parent trial for ancillary studies that could make further important contributions to the field. It is likely that ancillary studies will be conducted at a subset of sites due to the need 1) for specific expertise and/or equipment that may not be available at all locations and 2) to avoid overwhelming potential CTN-0080 participants with study requests. An example of the former would be a study of brain development requiring pediatric neuroimaging. Another example of an ancillary study, the pursuit of which will be encouraged by interested investigators, would be a lactation sub-study to assess the exposure of the infants to buprenorphine and/or buprenorphine-naloxone through breastfeeding. There are a limited number of lactation studies with women maintained on BUP-SL,[64-67] with the largest study including 10 women.[67] To date, there are no published lactation studies for breastfeeding women maintained on BUP/NX-SL. Thus, even a relatively small lactation sub-study could contribute valuable information to the field.

4.4 Conceptual Model

A secondary objective of MOMs is to test a conceptual model of the mechanisms by which BUP-XR may improve mother and infant outcomes relative to BUP-SL; the conceptual models to be tested are outlined in ***Figures 2a*** (mother outcomes) and ***2b*** (infant outcomes).

Fig. 2a: MOMs Conceptual Model: Mother Outcomes

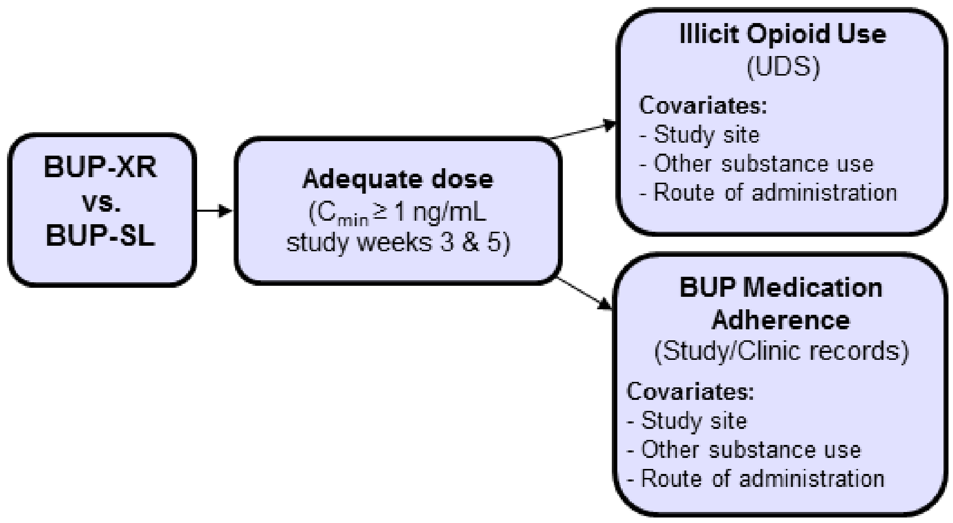

4.4.1 Mother Outcomes

The conceptual model describing the mechanism by which BUP-XR may improve mother outcomes relative to BUP-SL is outlined in ***Figure 2a***. As noted above, BUP plasma concentrations ≥1 ng/mL should be sufficient for suppressing opioid withdrawal[27] and, for the subset of participants using illicit opioids, BUP plasma concentrations ≥1.25 ng/mL should be sufficient to block the illicit opioid effect.[30] The main conceptual model will focus on the ≥1 ng/mL threshold since this should apply to all of the participants whereas the ≥1.25 ng/mL threshold is pertinent for participants using illicit opioids; the ≥1.25 ng/mL threshold will be evaluated in exploratory analyses. There is evidence that pregnant women may have BUP plasma concentrations <1 ng/mL for most of their dosing interval.[31] It has been hypothesized that inadequate BUP plasma concentrations may account for illicit opioid use and treatment dropout in pregnant women treated with BUP-SL.[26] For participants who consent to the CMA sub-study, trough BUP plasma levels (C_{min}) will be assessed in conjunction with the study week 3 and 5 visits. Based on the half-life of BUP-XR,[29] it is estimated that steady state will be reached at approximately study days 20-25; thus, steady state should be reached by study week 5. The rationale for including the week 3 sample is that, in order to predict treatment dropout, data need to be obtained from participants who subsequently dropout and research suggests that BUP dropout tends to occur within the first 30 days of treatment.[2,16] Based on its PK profile, BUP-XR, relative to daily BUP-SL, should result in a greater proportion of women with C_{min} ≥1 ng/mL, which, in turn, is hypothesized to result in less illicit opioid use and better BUP medication adherence. However, the difference in C_{min} between the BUP-XR and BUP-SL groups should be less pronounced in women following a split dosing schedule (i.e., taking BUP-SL more than once a day) or a higher dose; thus, BUP-SL dosing frequency and dose will be used to define BUP-SL subgroups. Three covariates that are likely to impact mother outcomes will be controlled for in the analyses: 1) study site (e.g., due to the provision of different psychosocial services, etc.); 2) use of other substances; and 3) route of administration of illicit opioid use (intravenous vs. not intravenous). It is important to note that C_{min} is being measured to test a conceptual model; there is insufficient evidence to support its use in guiding BUP dosing decisions and the results will not be given to the treating provider.

4.4.2 Infant Outcomes

The conceptual model describing the mechanisms by which BUP-XR may improve infant outcomes relative to BUP-SL is provided in ***Figure 2b***. In this conceptual model, fetal behavior, as measured by fetal heart rate variability (FHRV)[68,69] at maternal BUP C_{max} at ~36 weeks EGA, is hypothesized to be directly related to both infant development and NOWS-related outcomes. Fetal exposure, as indicated by cord norbuprenorphine plasma levels, is hypothesized to be related to NOWS-related outcomes and NOWS-related outcomes are hypothesized to be related to infant development.

Fig. 2b: MOMs Conceptual Model: Infant Outcomes

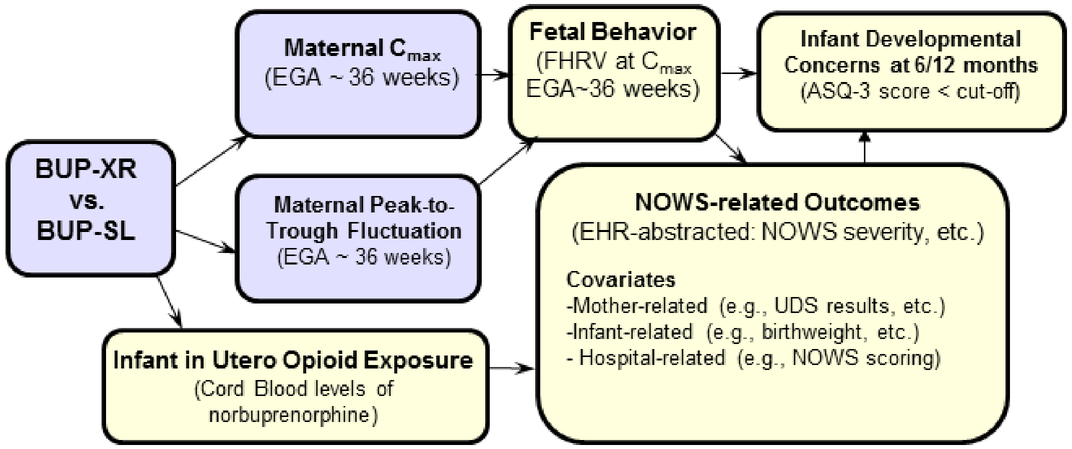

<u>Maternal peak BUP plasma level (C_{max}) and fetal behavior</u>. BUP C_{max} is associated with adverse fetal effects, including decreased FHRV.[24] PK data suggest that the 24 mg weekly formulation of CAM2038, relative to 16 mg of daily BUP-SL, has a lower C_{max} (4.97 ng/mL vs. 6.09 ng/mL) level.[42] It is thus predicted that the BUP-XR group will have lower C_{max}, relative to the BUP-SL group, and, thus, have greater FHRV. However, the difference should be less pronounced in women following a split dosing schedule or at a lower dose; thus, BUP-SL dosing schedule and dose will be used to define BUP-SL subgroups.

<u>Maternal peak-to-trough fluctuation and fetal behavior</u>. It has been hypothesized that the daily peak-to-trough fluctuation associated with opioid maintenance treatment results in the fetus experiencing withdrawal, which negatively affects fetal health.[70] It is predicted that the greater the maternal peak-to-trough fluctuation, the greater the level of withdrawal the fetus will experience. PK data suggest that the 24 mg weekly formulation of CAM2038, relative to 16 mg of daily BUP-SL, has both lower C_{max} (4.97 ng/mL vs. 6.09 ng/mL) and higher C_{min} (1.18 ng/mL vs. 0.85 ng/mL) levels.[42] It is, thus, predicted that the BUP-XR group will have a smaller peak-to-trough fluctuation, relative to the BUP-SL group, and, thus, have greater FHRV. However, the difference should be less pronounced in women following a split dosing schedule or at a lower dose; thus, BUP-SL dosing schedule and dose will be used to define BUP-SL subgroups.

<u>**Infant Development**</u>. Recent reviews have noted the need to consider the longer-term impact of in utero exposure to opioid maintenance on children, for which there is currently a dearth of research.[14] The 6-month and 12-month versions of the Ages and Stages Questionnaire, third edition (ASQ-3)[71] will be used to screen infants for developmental issues. The ASQ-3 is a validated, parent-administered screen used throughout the world[71-73] and deemed appropriate for assessing infants exposed to opioids in utero.[13] An association between FHRV and development has been found in a number of studies.[74-76] Furthermore, a recent study found that the relationship between maternal depression and neonatal neurobehavioral immaturity was mediated by FHRV.[77] It is thus predicted that fetal behavior, as measured by FHRV, will be a significant predictor of scoring below the ASQ-3 cut-off. Research has also found a relationship between NOWS severity and infant development[78, 79] and it is, thus, predicted that NOWS-related outcomes will predict ASQ-3 scores.

NOWS-related outcomes. Approximately 50% of infants born to women on opioid maintenance treatment will experience NAS/NOWS.[14] Factors that can impact NOWS include maternal polysubstance use,[32, 80] EGA at delivery and birth weight,[81, 82] and nonpharmacological treatment approaches.[83] Controlling for these factors as needed, the mechanisms by which BUP-XR, relative to BUP-SL, may impact NOWS-related outcomes will be evaluated. It has been hypothesized that the daily peak-to-trough fluctuation associated with opioid maintenance treatment results in the fetus experiencing withdrawal, which increases the incidence and severity of NAS/NOWS.[70] Consistent with this hypothesis, a retrospective study evaluating NAS/NOWS incidence in infants born to mothers maintained on a split-dosing schedule of methadone reported a lower incidence of NAS/NOWS (29%)[70] relative to the 50% typically associated with methadone maintenance.[14, 83] It is thus predicted that fetal behavior, as measured by FHRV, will be a significant predictor of NOWS outcomes.

Infant in utero opioid exposure and NOWS-related outcomes. Another key factor in determining NOWS severity is, of course, infant in utero opioid exposure. Research suggests that exposure to norbuprenorphine, a major active metabolite of BUP, may play an important role in the development of NOWS. EGA at delivery is positively associated with NAS/NOWS severity[81, 82] and placenta conversion of BUP to norbuprenorphine increases with increasing EGA.[84] Research has found a significant positive association between infant norbuprenorphine levels and hospital LOS.[85] In addition, a recent study found a significant positive association between norbuprenorphine levels in cord blood and the need for pharmacotherapy for NAS/NOWS.[82] While data on placenta conversion of BUP to norbuprenorphine is not available for BUP-XR, PK data suggest that there is less first-pass metabolism of BUP to norbuprenorphine for BUP-XR, with norbuprenorphine:BUP ratios 3-7 times lower for BUP-XR compared to BUP-SL.[86] Hence, it is predicted that norbuprenorphine levels in infants, as assessed in cord blood, will be significantly lower in the BUP-XR, relative to BUP-SL, group, which will, in turn, be predictive of lower NOWS severity. Assessing buprenorphine and its metabolites in cord blood offers an additional advantage in understanding the relationship between maternal BUP treatment and NOWS. Specifically, genetics likely play a role in infant in utero opioid exposure [87, 88] but much work remains to fully elucidate the genetic factors involved.[84, 89] It has been suggested that evaluating BUP and its metabolites in cord blood allows an evaluation of opioid exposure that reflects the end product of maternal, placental, and fetal genetics[82] and, thus, genetics will be taken into account in this manner.

4.5 Health Economics

NAS/NOWS and OUD are public health priorities. As mentioned above, NAS/NOWS is associated with adverse health effects for the infant[7-9] and with costly hospitalizations.[6] OUD is associated with lower health-related quality of life (HRQoL)[90]; excess high-cost healthcare utilization (e.g., emergency department visits and inpatient admissions)[91, 92]; and other adverse personal and social consequences that, in total, cost the U.S. over $500 billion, annually.[93] BUP-SL has been shown to be effective and cost-effective in treating OUD, resulting in improved HRQoL and reduced healthcare costs; however, evidence of these effects among pregnant women/new mothers with OUD does not exist.[94] As also noted above, adherence to OUD pharmacotherapy can be problematic among pregnant women,[14, 20] an issue that can be addressed with XR formulations;[36, 37] however, the additional cost of BUP-XR may serve as a barrier to adoption. The wholesale acquisition costs for CAM2038 have not been established. The current wholesale acquisition costs per monthly dose of Sublocade™ is $1,580, versus $489 and $406 for monthly doses of 16mg/day buprenorphine-naloxone film (Suboxone®) and generic tablets, respectively.[95] Focusing on the cost of the therapy in isolation is shortsighted, as it does not account for the many potential value offsets associated with effective treatment of

OUD; understanding the relative costs/benefits of BUP-XR is thus a tertiary objective of the MOMs trial.

4.6 Infant Neurodevelopment

There have been relatively few well-designed studies of neurodevelopmental outcomes among children prenatally exposed to opioid agonist medications. The reported data have been sparse and often conflicting[96] and this represents a substantial knowledge gap.[14] A systematic review and meta-analysis from 2014 was able to include only five studies quantitatively reporting neurobehavioral function, and the included studies were all assessed as “weak” or “moderate” quality, with relatively small samples.[97] Though there were trends for poorer outcomes, the meta-analysis showed no significant impairments for the children exposed to chronic opioids in-utero, compared to the non-exposed children. A recent retrospective chart review suggested deleterious neurodevelopmental effects of chronic in-utero opioid exposure,[78] but considerable methodological issues with the study design and its interpretation have been noted.[98] The results from a prospective, longitudinal study of 96 infants from the MOTHER trial suggest that there were no significant adverse neurodevelopmental effects from either prenatal opioid exposure (with no difference between methadone- and buprenorphine-maintenance) or opioid treatment for NAS/NOWS.[99]

In light of the relative dearth of high-quality research data available on this important topic, the MOMs trial presents an opportunity to conduct neurodevelopmental assessments on a relatively large sample of children with prenatal exposure to buprenorphine and to evaluate these data while controlling for a number of prospectively collected contributing factors.[98, 100] The CTN-0080 parent trial includes the collection of the Ages and Stages Questionnaire, third edition (ASQ-3) at two time points – when the infant is approximately 6 and 12 months of age (see ***section 7.2.2.3***). The ASQ-3 is a validated, parent-administered screen used throughout the world[71-73] and deemed appropriate for assessing infants exposed to opioids in utero.[13] In addition, the ASQ-3 can be completed by the caregiver in his/her home and mailed to the research team; this convenience might serve to increase completion rates. However, some research has found relatively poor agreement between the ASQ-3 and the Bayley™-4, which is considered to be a gold standard assessment of early child development.[101]

Thus, a quaternary objective of the MOMs trial is to evaluate the impact of BUP-XR, relative to BUP-SL, on infant neurodevelopment as measured by the Bayley™-4 when the infant/child is approximately 12 and 24 months of age (see ***section 8.10***).

5.0 STUDY OBJECTIVES

5.1 Primary Objective

The primary objective of this trial is to evaluate the impact of treating OUD in pregnant women with BUP-XR, compared to BUP-SL, on mother and infant outcomes. It is hypothesized that the BUP-XR, relative to the BUP-SL, group will:

1) not have greater illicit opioid use during pregnancy (primary, non-inferiority);
2) have lower infant neonatal opioid withdrawal syndrome (NOWS) severity (key secondary, superiority); and
3) not have greater postpartum illicit opioid use (key secondary, non-inferiority).

5.2 Secondary Objective

Testing a conceptual model of the mechanisms by which BUP-XR may improve mother-infant outcomes, relative to BUP-SL, is a secondary trial objective.

5.3 Tertiary Objective

To determine the economic value of BUP-XR, compared to BUP-SL, in the treatment of OUD among pregnant mothers by evaluating the cost of each treatment strategy, changes in the utilization of associated treatment and other healthcare services, and improvements in health-related quality-of-life (HRQoL), measured as quality-adjusted life-years (QALYs), is a tertiary trial objective. We anticipate that a larger reduction in the utilization of high-cost healthcare services and increase in QALYs will result in BUP-XR being cost-effective compared to BUP-SL from a healthcare sector perspective, according to traditionally accepted value thresholds.

5.4 Quaternary Objective

The quaternary objective is to evaluate the impact of BUP-XR, relative to BUP-SL, on neurodevelopment when the infant/child is approximately 12 and 24 months of age. The main outcome of interest is the score on the cognitive subscale of the Bayley™-4[102] at the 24-month assessment. Based on the outcomes of the MOTHER neurodevelopmental study,[99] it is predicted that prenatal buprenorphine exposure will not result in significant adverse neurodevelopmental outcomes and that there will be no difference between the BUP-XR and BUP-SL treatment arms.

6.0 STUDY DESIGN

6.1 Overview of Study Design

This is an intent-to-treat, two-arm, open-label, pragmatic RCT. Eligible participants will be randomized in a 1:1 ratio to BUP-XR or BUP-SL, balancing on site, EGA at time of randomization (6 weeks – 18 weeks vs. 19 weeks – 30 weeks), and whether they are on BUP-SL at the time of randomization (yes vs. no). Participants will be provided BUP-XR/BUP-SL through 12 months postpartum. Key outcome measures are: 1) the proportion of illicit opioid-negative urines during pregnancy (primary); 2) infant's NOWS severity, as assessed by total days of opioid treatment derived from the infant's medical record (key secondary); and 3) proportion of illicit opioid-negative urines during the 12-month postpartum phase (key secondary). Safety measures will include adverse events, mood measures, and delivery-related outcomes.

6.2 Number of Sites and Participants

Approximately 200 participants, recruited from approximately 10 sites, will be randomized into the trial. Pregnant patients who have an EGA of 6 - 30 weeks at randomization, and, in the judgment of the treating provider, are good candidates for BUP-maintenance treatment will be recruited for the study. Participants may be recruited from a variety of other sources, including advertising, but must have completed intake at a study site to be eligible for randomization. Recruitment advertisements will be approved by the Institutional Review Board (IRB). Efforts will be made to recruit a study sample that reflects, or exceeds, the proportion of minorities in treatment at the sites.

6.3 Study Duration

Sites will be initiated on a rolling basis. Once all sites are initiated, enrollment is expected to take place over a period of approximately 32 months. Duration of participation may vary from approximately 15 months to 22 months (e.g., depending on the time taken to complete screening/baseline, EGA at enrollment, etc.) for the main CTN-0080 trial.

6.4 Site and Participant Selection

6.4.1 Site Selection

A pragmatic trial should include a mix of sites/investigators that are representative of real world practice and that possess the skills to effectively implement the study intervention.[45] To this end, CTN-0080 seeks to include a mix of academically-affiliated and community clinics. Evaluating BUP-XR, compared to BUP-SL, in the treatment of pregnant women with OUD under real world conditions requires that sites already have experience with BUP-SL dosing in pregnant women. The majority of the site eligibility criteria outlined in ***section 6.4.1.1*** are the minimum requirements needed to successfully implement MOMs. The criterion related to delivery hospitals is included to reduce variability in neonatal outcomes and to ensure that important covariates can be collected and accounted for in the data analyses. The recommended model of care for pregnant women with OUD is one in which there is close collaboration between prenatal care and addiction treatment providers and, where possible, integrated treatment.[13] This approach will be used by all sites eligible for CTN-0080, but the treatment components will likely vary among sites. A review of the literature failed to identify an instrument for characterizing models of care for the management of pregnant women with OUD; such an

assessment will be developed and used to characterize treatment at the MOMs sites (*see **section 7.4***).

6.4.1.1 *Site Characteristics*

Participating sites should:

1. provide BUP to pregnant women in an "office-based" setting (i.e., outside of a treatment program requiring daily/near-daily in-clinic administration);
2. offer OUD treatment (including BUP) for patients following delivery for ≥12 months;
3. include a close collaboration between prenatal care and addiction treatment providers;
4. be willing to comply with study procedures, including weekly Medication Check Visits with participants;
5. enroll enough potentially eligible patients to meet the target randomization of 1.25 per month;
6. have access to a medical clinician who can prescribe BUP (the degree and licensing requirements depend on the regulations of the state in which the site is located), to determine participant eligibility, and to regulate the medication dose appropriately;
7. have access to, or the ability to contract with, a pharmacy/pharmacist (or other appropriately qualified entity based on local/state regulations) to store/dispense study medications;
8. be able to provide after-hours clinical back-up for study-related emergencies;
9. have access to, or the ability to contract with, a phlebotomist or other appropriate professional, to complete blood draws;
10. have ≥75% of their pregnant patients delivering at hospitals that: a) have a written protocol for the management of NAS/NOWS; b) offer rooming-in while infants are being observed for NAS; c) do not send infants home on opioids for the treatment of NAS/NOWS; d) have agreed to complete the BORN survey (see ***section 7.4.2***) at two time-points (e.g., pre-site initiation and post-delivery for the final site participant).

6.4.2 Participant Selection

The eligibility criteria for a pragmatic trial should define participants representative of the patient pool for whom the intervention would be utilized.[45] The rationale for each eligibility criterion is provided in ***section 6.4.2.3***.

6.4.2.1 *Inclusion Criteria*

Potential participants <u>must</u>:

1. be 18-41 years of age;
2. be pregnant with an EGA of 6 - 30 weeks at randomization, have evidence of a viable intrauterine pregnancy (IUP) if EGA <12 weeks, and is not planning to terminate the pregnancy;
3. have a single fetus pregnancy (can be based on self-report if an objective assessment is unavailable);

4. meet DSM-5 criteria for moderate/severe OUD and be a good candidate for BUP maintenance and/or be currently prescribed BUP for the treatment of OUD;
5. be willing to be randomized to BUP-XR or BUP-SL and to comply with study procedures, including weekly Medication Check Visits;
6. be planning to deliver at one of the hospitals for which the BORN survey was completed and that: a) has a written protocol for the management of NAS/NOWS, b) offers rooming-in while infants are being observed for NAS/NOWS; and c) does not send infants home on opioids for the treatment of NAS/NOWS;
7. be enrolled in outpatient addiction treatment at a participating site (e.g., have completed intake);
8. be able to understand the study, and having understood, provide written informed consent in English.

6.4.2.2 *Exclusion Criteria*

Potential participants must not:

1. have a physiological dependence on alcohol or sedatives requiring medical detoxification;
2. have a psychiatric condition that, in the judgment of the site medical clinician (MC), would make study participation unsafe or which would make treatment compliance difficult;

   Examples include:

   - Suicidal or homicidal ideation requiring immediate attention
   - Severe, inadequately-treated mental health disorder (e.g., active psychosis, uncontrolled bipolar disorder)
3. have a medical condition that, in the judgment of the site MC, would make study participation unsafe or which would make treatment compliance difficult. Medical conditions that may compromise participant safety or study conduct include, but are not limited to, allergy/sensitivity to study medications and the following based on clinical labs:
   - AST/ALT greater than 5X upper limit of normal
   - serum creatinine greater than 1.5X upper limit of normal
   - total bilirubin greater than 1.5X upper limit of normal
4. be currently in jail, prison, or any inpatient overnight facility as required by court of law or have pending legal action or other situation (e.g., unstable living arrangements) that, in the judgement of the site investigator, could prevent participation in the study or in any study activities;
5. be currently receiving methadone or naltrexone treatment;
6. be enrolled in or planning to enroll in treatment beyond the level 3.3 (Clinically Managed Population-Specific High-Intensity Residential Services) of the American Society of Addiction Medicine criteria; for level 3.3, the participant must have the ability to leave the facility unaccompanied by staff as needed;[103]
7. be enrolled in or planning to enroll in: a) a trial testing medication for managing OUD during pregnancy; b) research testing an intervention for substance use disorder or

NOWS in their infant unless they are willing to provide a release for the research records.

6.4.2.3 *Rationale for Eligibility Criteria*

The rationale for each inclusion and exclusion criterion is provided in ***Table 1***. Some criteria were selected to reflect the eligibility criteria from the MOTHER study[43] in order to increase the comparability of the MOTHER and MOMs study samples.

Table 1: Rationale for Study Eligibility Criteria

Criterion#	Criterion Description	Criterion Rationale
I1	18-41 years of age	Definition of Study Sample (adults); >41 year of age associated with obstetrical problems; MOTHER eligibility criterion
I2	Pregnant, 6-30 weeks EGA; evidence of viable IUP if EGA <12 weeks; not planning to terminate	Definition of Study Sample (pregnant); MOTHER eligibility criterion
I3	Single fetus pregnancy	Multiple fetuses associated with poorer neonatal outcomes; MOTHER eligibility criterion
I4	DSM-5 criteria for OUD, BUP candidate; prescribed BUP	Definition of Study Sample (candidates for BUP-maintenance treatment)
I5	Willing to be randomized; comply with study procedures	To help ensure that the participant will provide useful data
I6	Delivery hospital criteria	NOWS approach standardization
I7	Completed intake for treatment at study site	Required by study design (evaluating medication within Substance Use Disorder [SUD] treatment context)
I8	Understand study/give consent	Good Clinical Practice (GCP) Requirement
E1	Alcohol/sedative physiological dependence	Safety
E2	Psychiatric condition making participation unsafe/difficult	Safety and to help ensure that the participant will provide useful data
E3	Medical condition making participation unsafe/difficult	Safety and to help ensure that the participant will provide useful data
E4	Situation that could prevent participation in the study/study activities	To help ensure that the participant will provide useful data
E5	Taking methadone/naltrexone	Contraindication
E6	Beyond 3.1 treatment level	To reduce sample heterogeneity
E7	Other research participation	Would adversely impact study validity (a) or may add variance that needs to be accounted for (b)

7.0 STUDY MEASURES

Ideally, pragmatic trial measures would be obtained unobtrusively in order to reduce participant burden and to avoid research assessments/interactions that could impact outcomes and, thus, reduce the generalizability of the results to real world practice; however, some outcomes can only be obtained with participant input.[45] CTN-0080 is designed to rely, as much as possible, on medical record data and to collect data directly from participants only when measures reflect important outcomes or are needed to interpret outcomes (i.e., explanatory variables).

7.1 Key Outcome Measures of the Primary Objective

7.1.1 Primary Outcome - Illicit Opioid Abstinence During Pregnancy

Avoiding illicit opioid use is a key rationale for providing opioid maintenance therapy to pregnant women with OUD. While BUP-SL is effective in reducing illicit opioid use, the CAM2038 Phase 3 trial revealed that CAM2038 was superior to BUP/NX-SL on the proportion of illicit opioid-negative urine samples.[104] MOMs will use proportion of illicit opioid-negative urine samples during pregnancy as the primary outcome.

Urine samples will be collected at the time of the weekly Medication Check Visits, ideally using temperature monitoring, and the validity of urine samples will be checked with the use of a commercially available adulterant test. In cases where the temperature reading (when available) or adulterant test indicates a non-valid sample, an attempt will be made to obtain a second urine sample. Samples will be shipped to a central lab for analysis using a rapid UDS system. Urine samples will be tested for: buprenorphine/ norbuprenorphine, fentanyl, cocaine, methamphetamine, amphetamine, opioids, marijuana, benzodiazepines, methylenedioxymethamphetamine (MDMA, Ecstasy), barbiturates, methadone, oxycodone, phencyclidine (PCP), cotinine, and ethyl glucuronide, which is a biomarker of alcohol consumption. The UDS results will not be provided to the study site staff. The UDS system to be utilized has a 0% false positive rate as determined by comparing the results of reported accuracy test samples[105, 106] with standard GC/MS cut-offs for each substance.[107, 108] However, as noted by SAMHSA[13] and ACOG,[109] there is a possibility for false positives with any qualitative test (due to cross-reactions, etc.); hence, *in a clinical scenario*, a positive result *should be subjected to confirmatory testing*, given its potential ramifications (e.g., potential reporting requirements). Given the low false positive rate of the UDS system being utilized and the fact that research results will not be utilized for clinical decisions, we feel confident that confirmatory testing is not necessary. For primary outcome scoring, missing urine samples will be imputed as positive for illicit opioids, which is consistent with the approach taken in the CAM2038 Phase 3 trial[104] and with the greater likelihood of illicit opioid use in patients not engaged in treatment.[110, 111] The number of UDSs expected for each participant will differ based on the length of the pregnancy, thus the number of UDSs potentially imputed will also differ for each participant.

7.1.2 Key Secondary Outcomes (Primary Objective)

7.1.2.1 *NOWS Severity*

NOWS severity will be assessed by total days of opioid treatment during the hospital stay, which is a definition that has been used in past research.[60] This outcome will be abstracted from the medical record.

7.1.2.2 *Postpartum Illicit Opioid Abstinence*

Illicit opioid abstinence postpartum will be assessed in a similar fashion to illicit opioid abstinence during pregnancy (see **section *7.1.1***).

7.2 Secondary Outcome Measures (Primary Objective)

7.2.1 Mother Secondary Outcomes

7.2.1.1 *BUP Medication Adherence*

Adherence to BUP treatment during pregnancy through 12 months postpartum is a clinically important outcome due to the heightened vulnerability to relapse[13] and overdose[22] during this period. Adherence to BUP treatment during pregnancy will be scored as the number of days of adherence divided by the number of days between randomization and delivery. Postpartum adherence to BUP treatment will be scored as the number of days of adherence post-hospital-discharge divided by the number of post-hospital-discharge days in the 12-month postpartum phase. Adherence to BUP-XR will be based on study records (i.e., BUP-XR Injection documentation). For BUP-XR, the receipt of a weekly injection will be scored as 7 days of adherence. If a particular day is covered by two injection windows (e.g., injections on study day 7 and study day 13, where study day 6 overlaps the two seven-day windows), the adherent day will only be counted once. For monthly injections, a participant will be considered as adherent for 28 days and overlapping intervals are handled similarly as for the weekly injections. Adherence to BUP-SL will be defined as: 1) study records showing that BUP-SL was dispensed to the participant (i.e., BUP-SL Dispensing documentation); 2) self-reported adherence will be assessed at the weekly Medication Check Visits; and 3) UDSs positive for buprenorphine/norbuprenorphine. In cases where participants discontinue study medication to transfer to an alternative treatment (e.g., methadone, extended-release naltrexone), a score of 0 days of adherence will be given for the time period post discontinuation. The rationale for not "crediting" receipt of methadone or extended-release naltrexone as adherence is that a switch to an alternative medication is an indicator that BUP-XR / BUP-SL was, in some way, ineffective for the participant and the score of 0 is consistent with the lack of effectiveness. In cases where participants discontinue study medication to transfer to BUP from an alternative provider or were provided non-study BUP during hospital stays or incarceration, a release of information will be obtained, and adherence will be based on clinic/pharmacy records (BUP-XR) or, for BUP-SL, on clinic/pharmacy records along with participant self-report and study UDSs if available. For the case where clinic/pharmacy records could not be obtained for any reason, the participant will be considered non-adherent. If participants drop out of the study prematurely, they will be considered non-adherent for all days after drop-out. In the case of intermittent missing UDSs (e.g., UDSs not collected for any reason but the participant did not drop out), then adherence will be based on study/clinic/pharmacy records and self-report.

7.2.1.2 *Drug and Alcohol Abstinence*

Drug and alcohol abstinence during pregnancy and postpartum are secondary outcomes and will be assessed in a fashion similar to the assessment of illicit opioid abstinence, but rather than being restricted to illicit opioid use, will include alcohol and other drugs of abuse. Missing urine samples will be imputed as positive.

7.2.1.3 *The Opioid Craving Scale*

Craving will be assessed with the Opioid Craving Scale, which was utilized in CTN-0030 and shown to have predictive validity.[112] The total score is calculated by averaging the scores from

three visual analogue scales which assess craving, cue-induced craving, and likelihood of using.

7.2.1.4 *Adequacy of Prenatal Care Utilization*

Kotelchuck's Adequacy of Prenatal Care Utilization (APNCU) index,[113] which is a well-established index,[114] will be used to assess the adequacy of prenatal care. The APNCU includes two indices that are combined to obtain a total score. One index is the timing of prenatal care initiation scored in 4 categories [EGA months: 1) 1 and 2; 2) 3 and 4; 3) 5 and 6; and 4) 7 to 9]. The second is the ratio of observed to expected visits based on the length of time between the first prenatal care visit and delivery, scored in 4 categories: 1) Inadequate (received less than 50% of expected visits); 2) Intermediate (50%-79%); 3) Adequate (80%-109%); 4) Adequate Plus (110% or more). The information for the APNCU will be derived from either medical records or the birth certificate. The % of expected visits attended prior to study randomization will be included as a baseline covariate in analyses.

7.2.1.5 *The Short Opiate Withdrawal Scale (SOWS)-Gossop*

The SOWS-Gossop[115] will be used to measure opioid withdrawal symptoms. The SOWS-Gossop, which is a self-administered scale that includes 10 items, rated on a scale of 0 (none) to 3 (severe), is a validated instrument with good reliability.[116] It will be completed following the schedule in ***Table 3***.

7.2.2 Infant Secondary Outcomes

7.2.2.1 *Other NOWS-related Outcomes*

Five additional NOWS-related outcomes will be abstracted from the medical record:

1. use of opioid medication for NOWS symptoms (yes/no); if yes, medication used;
2. infant hospital LOS defined as the infant's age, in days, at discharge;
3. use of adjunct medications (e.g., phenobarbital, clonidine);
4. NOWS scoring assessment used and peak score; and
5. an ICD-10 code indicative of NOWS (Yes/No); infants will be scored as "yes" if their medical record includes an ICD-10 code of P96.1 (neonatal withdrawal symptoms from maternal use of drugs of addiction) and/or P96.2 (withdrawal symptoms from therapeutic use of drugs in newborn) within the first 10 days of life.

7.2.2.2 *Discharge Outcomes*

Discharge outcomes to be abstracted from the medical record include: custody (e.g., mother, other relative, foster/adoptive family), medications at discharge (e.g., phenobarbital, clonidine), and an open case with child protective services (yes/no).

7.2.2.3 *Infant Development*

The 6- and 12-month versions of the Ages and Stages Questionnaire, third edition (ASQ-3)[71] will be used to screen for developmental issues in the infants. The ASQ-3 is a validated, parent-administered screen used throughout the world[71-73] and deemed appropriate for assessing infants exposed to opioids in utero.[13] The outcome of interest is whether or not the infant scores below the ASQ-3 cut-off (yes/no). The ASQ-3 will be completed by the infant's caregiver, which

may or may not be the birth mother. Thus, flexibility in the collection of this assessment will be allowed, including collection via mail. Infant development can be significantly impacted by psychosocial stressors and, thus, the individual completing the ASQ-3 will also be asked to complete The Family Psychosocial Screener (*see* ***section 7.6.3***). Caregivers of infants scoring below the cut-off will be offered a referral for further evaluation.

7.3 Safety Measures of the Primary Objective

7.3.1 Adverse Events (AEs)

AEs will be assessed by study staff as outlined in ***Table 3***. If an AE requires medical attention, it should be reported to a site MC immediately. For visits held via telemedicine or at an external location, research staff will contact a qualified medical clinician via phone to coordinate an assessment via telemedicine as quickly as possible; ideally this assessment will occur while the staff and participant are together at the external location. Recording of safety information, including Adverse Events, is described in **section 11.17**.

7.3.2 The Hospital Anxiety and Depression Scale (HADS)

The Hospital Anxiety and Depression Scale (HADS)[117] will be used to assess for symptoms of depression and anxiety. The HADS is a brief, validated instrument that screens for both depression and anxiety[118] and will be completed following the schedule outlined in ***Table 3***. Participants who score in the range for possible depression (total depression score of 8 or higher) or anxiety (total anxiety score of 8 or higher) should be assessed by a qualified clinician before leaving the clinic as specified in the site clinical standard operating procedure (SOP). For visits held via telemedicine or at an external location, research staff will contact a qualified clinician via phone to coordinate an assessment via telemedicine as quickly as possible; ideally this assessment will occur while the staff and participant are together at the external location. If a participant is assessed and, at subsequent visits, continues to have the same elevated score, then the need for reassessment is at the discretion of a qualified clinician; a subsequent increase in the HADS score would require assessment by a qualified clinician.

7.3.3 Prior/Concomitant Medications

All medications taken by the participant since the start of her pregnancy and during the active study will be documented on a Prior/Concomitant Medications assessment (see ***Table 3***). All medications taken by the participant while in the study should ideally be pre-approved by the MC whenever possible to avoid interactions with the study drugs.

7.3.4 Fetal Outcomes

Adverse fetal outcomes, including gestational age at time of outcome, will be abstracted from the medical record. These outcomes include: spontaneous abortions/miscarriages, stillbirth (i.e., death of a fetus at any time after the 20th week of pregnancy), pregnancy terminations and indications for termination (elective or medical and, if medical, the reason for termination).

7.3.5 Maternal Delivery Outcomes

Maternal delivery outcomes to be abstracted from the medical record include: cesarean section, abnormal fetal presentation during delivery, medical complications at delivery, and analgesic receipt during labor and delivery, postpartum, and upon discharge.

7.3.6 Birth/Neonatal Outcomes

Birth/neonatal outcomes will be abstracted from the medical record. These outcomes include: head circumference, weight and length at birth, gestational age at delivery, and 1-minute and 5-minute Apgar (activity, pulse, grimace, appearance, respiration) scores. Other outcomes will include: major birth defects, neonatal death, and need for resuscitation. Adverse birth outcomes will be characterized, including (if preterm), the reason for preterm birth (e.g., fetal distress, etc.). Other adverse neonatal outcomes will be captured including respiratory distress symptoms, need for respiratory support in the neonatal unit, feeding problems (need for nasogastric tube), seizures, and other co-morbidities.

7.3.7 Injection Site Examination

Participants will be asked to immediately report any injection site reactions to study staff for evaluation, monitoring, and possible referral, as needed. For weekly injections, the site of the last BUP-XR injection will be examined at the weekly Medication Check Visits. For monthly injections, the BUP-XR injection site will be examined at the weekly Medication Check Visit following the visit during which the injection was given. Injection site reactions will be documented on the Injection Site Reaction Reporting form.

7.3.8 Opioid Overdose Tracking

Some evidence suggests that women with OUD are at heightened risk for overdose during the postpartum period.[22] During the study, the participant will complete a self-report assessment about opioid overdose as outlined in ***Table 3***. If a participant reports an overdose, research staff will refer her to the clinic staff who will follow up each case as specified in the site clinical SOP. A self-reported overdose resulting in naloxone rescue will be collected as an SAE.

7.3.9 Infant Sedation

The limited data available has revealed low levels of BUP and BUP metabolites in the breastmilk of BUP-maintained women[119] and breastfeeding is recommended for stable BUP-maintained women.[120] However, more data on potential infant exposure through breastfeeding is needed for BUP-maintained women and data on BUP/NX-maintained women[67] and BUP-XR-maintained women is lacking. While no adverse reactions are expected in breastfed infants in the present study, a theoretically possible and concerning adverse reaction, infant sedation, will be assessed via mother-report as outlined in ***Table 3***. The infant sedation assessment will be completed by participants who are feeding their infants with breastmilk and/or formula and will assess for signs of infant sedation (e.g., not waking for feeding, difficulty breathing, etc.). In addition, if an infant sedation assessment in a breastfed infant meets the criteria of an SAE, it will be collected as an SAE, as all infant SAEs occurring in breastfed infants will be collected.

7.4 Covariates

There are a number of variables with the potential to impact mother-infant outcomes and, thus, either mask or exaggerate study medication effects. Randomization will help ensure that the BUP-XR and BUP-SL groups are balanced on these variables, however, they can still be an important source of variance. Analyses will control for these variables, referred to as covariates.

<u>Pregnancy and Addiction Services Assessment (PAASA)</u>. Study site will be included as a covariate in the secondary analyses. As noted in ***section 6.4.1***, all MOMs sites will use a collaborative care model but will likely differ in the specifics of the care model used (e.g., psychosocial treatment provided etc.), which could impact mother outcomes. A review of the

literature failed to identify an instrument for characterizing models of care for the management of pregnant women with OUD. Hence, an assessment was developed for use in this protocol, the PAASA, which will be used to characterize the treatment provided by the MOMs sites. The PAASA will also be used for the health economic analysis (see ***section 10.9***); the PAASA data will likely need to be supplemented with data collected through semi-structured interviews (e.g., can be collected by e-mail correspondence, phone, or in-person interviews) for this purpose. In CTN-0080, a staff member from each site will complete the PAASA at two time-points (i.e., pre-site initiation and near the end of participant data collection).

7.4.1 Covariates for Mother Outcomes

Two covariates will be controlled for in the mother outcomes in addition to study site: 1) the use of other substances at baseline, which can impact both treatment adherence and illicit opioid use and 2) route of illicit opioid use (intravenous vs. not intravenous); the Phase 3 CAM2038 trial found that illicit use outcomes were significantly better for CAM2038, relative to BUP-SL, for participants with intravenous use.[59]

7.4.2 Covariates for Infant Outcomes

The list of potential covariates for infant outcomes is provided in ***Table 2***. The mother-related and infant-related covariates will be assessed in the process of collecting other outcome measures (e.g., UDS will capture substance use, including nicotine use, etc.) whereas the delivery hospital-related covariate needs to be obtained from the delivery hospitals; the BORN Survey will be used to obtain these data.

**Table 2. Covariates for Infant Outcomes**
Mother-related Covariates
Tobacco use[81, 121, 122]
UDS positive for: 1) substance of abuse; 2) illicit-opioids only[32, 80]
Exposure (yes vs. no) to psychiatric medications (e.g., antidepressants, benzodiazepines, gabapentin, etc.) during pregnancy[81, 123, 124]
Infant-related Covariates
EGA at delivery[81, 82]
Birthweight[81, 82]
Pharmacotherapy received for NAS/NOWS[125, 126]
Breastfed while in the hospital[127, 128]
NAS/NOWS scoring procedure utilized (e.g., Finnegan, Eat Sleep Console, other)[129, 130]
Delivery Hospital-related Covariate
Minimum days of observation for NAS/NOWS for infants exposed to long-acting opioids[131]

<u>BORN Survey.</u> The Better Outcomes Through Research for Newborns (BORN) network of the Academic Pediatric Association created the BORN survey, which assesses hospital characteristics and all aspects of NAS/NOWS management.[62] The BORN survey was recently used to assess practices for managing NAS/NOWS in BORN-affiliated hospitals; 76 hospitals, located in 34 states completed the BORN survey, with the results revealing significant diversity in approach across hospitals.[62] The delivery hospital-related covariate in ***Table 2*** will be assessed by having a staff member from delivery hospitals meeting the criteria outlined in ***section 6.4.1*** (i.e., written protocol for NAS/NOWS management, etc.) complete the BORN survey at two time-points; staff will be reimbursed for their time. If a participant delivers at a different hospital than originally planned, an attempt will be made to obtain a completed BORN survey for the hospital if one has not been obtained previously.

7.5 Health Economic Measures of the Tertiary Objective

The main economic outcome will be the incremental cost-effectiveness ratio (ICER), calculated as the incremental cost of BUP-XR relative to BUP-SL, divided by the incremental effectiveness of BUP-XR relative to BUP-SL. The main measure of effectiveness will be QALYs. The secondary measure of effectiveness will be Abstinent Years (a measure of time abstinent). The QALY is a measure that combines the HRQoL associated with an individual's health state and their time spent in that state, and is recommended as the main effectiveness measure in economic evaluation studies due to its ability to be compared across interventions and disorders, thereby enabling a broader economic interpretation.[132] In addition, generally accepted thresholds for defining value have been established for QALYs, unlike clinical measures.[133, 134] Also, HRQoL is increasingly recognized as a key indicator of patient well-being that is not captured in clinical measures.[135, 136] Time abstinent is an important measure of effectiveness for clinical stakeholders, and calculating cost-per-Abstinent-Year enables comparisons with existing economic evaluations that have utilized similar effectiveness measures, especially those that have relied solely on time abstinent measures.[94]

7.5.1 Healthcare Service Utilization

The utilization of healthcare services will be measured using medical records, the Treatment Services Review (see ***section 7.6.3***), and the Non-study Medical and Other Services (NMOS) form, which includes items assessing utilization of therapy for issues other than SUD, out-of-pocket healthcare expenditures, and type of insurance (if any). Healthcare services will include OUD treatment medications, residential and outpatient SUD treatment days; hospital SUD detoxification days; mental health treatment visits; and non-SUD inpatient, outpatient, and emergency department visits. The reliability and validity of self-reported data is well established over recall periods similar to those in our study.[137] The NMOS form has been successfully used in prior economic evaluations,[138, 139] including alongside CTN trials.[140, 141]

7.5.2 Health-related quality of life

Health-related quality of life (HRQoL) will be measured using the Patient-Reported Outcomes Measurement Information System (PROMIS).[142, 143] PROMIS was developed using item response theory, with support from the National Institutes of Health. Because of its foundation, PROMIS is able to improve upon common deficiencies of existing, widely-used, HRQoL instruments capable of generating a single health utility index value for the calculation of QALYs[144,145,146,147] including: floor and ceiling effects among participants who are especially ill or healthy, respectively, and imprecise questions that blend concepts.[148] The PROMIS-Preference (PROPr) scoring system uses the respondent's scores for each of the following PROMIS domains to calculate a health utility index value that represents the general US population's preference for the respondent's current health state: Cognitive Function–Abilities, Depression, Fatigue, Pain Interference, Physical Function, Sleep Disturbance, and Ability to Participate in Social Roles and Activities.[149, 150] PROMIS has 5 levels for each domain: no problems, slight problems, moderate problems, severe problems, and extreme problems. The health-utility value produced by PROPr can range from -0.022 to 1, where 0 represents death, 1 represents perfect health, and values below 0 represent states perceived to be worse than death. Construct validity for PROPr has been demonstrated using other HRQoL instruments and two large datasets from the general US population.[148] The health-utility value is then used to calculate QALYs, as our team, and many others have done in similar studies.[94, 138, 140, 141, 151]

7.5.3 Abstinent Year

The abstinent year will be operationalized as the predicted proportion of the year that the participant was abstinent from opioids. Opioid abstinence will be calculated using urine testing as described in ***sections 7.1.1*** and ***7.1.2***.

7.6 Other Measures

7.6.1 Screening Assessments

Pre-screen Interview: The pre-screen interview includes questions about pregnancy status, plans for treatment, and substance use.

PhenX Tier 1: The PhenX Tier 1 of the Substance Abuse and Addiction core[152] will be used to collect information on demographic characteristics (e.g., sex, age, ethnicity, etc.) and information on recent and lifetime use of tobacco, alcohol, and other substances.

The DSM-5 Checklist: The DSM-5 Checklist is a semi-structured, interviewer-administered instrument that will be used to assess for DSM-5 substance use disorders including: opioid, alcohol, amphetamine, cocaine, cannabis, and sedative. It will be completed during screening/ baseline.

Suicidal and Homicidal Screening Form (PRISM): The Suicide and Homicide Screening Form is a structured, reliable interview modified from the Psychiatric Research Interview for Substance and Mental Disorders- PRISM[153] and will be completed by study staff during screening/baseline. A qualified mental health professional must assess participants reporting current suicidal/ homicidal intent as specified in the site clinical SOP. For visits held via telemedicine or at an external location, research staff will contact a qualified clinician via phone to coordinate an assessment via telemedicine as quickly as possible; ideally this assessment will occur while the staff and participant are together at the external location.

Blood Chemistry: Clinical labs need to be reviewed during screening/baseline to assess eligibility. Results from labs completed within 30 days before consent can be utilized for this purpose. Otherwise, blood will be collected in serum separation evacuated venous blood collection tubes. Quantitative analysis will be performed, which will include the following analytes: glucose, creatinine, alanine aminotransferase (ALT/SGPT), aspartate aminotransferase (AST/SGOT), total bilirubin, and blood urea nitrogen (BUN). A prescription topical numbing cream may be offered to all participants prior to the blood draw.

Pregnancy Test: A urine pregnancy test designed to measure human chorionic gonadotropin hormone will be completed during screening/baseline to confirm the participant’s pregnancy status.

Estimated Gestational Age (EGA): If available, EGA will be abstracted from the medical record. Ideally, EGA will be defined based on an ultrasound completed as part of routine prenatal care. However, if an ultrasound is not available, then EGA will be based on last menstrual period (LMP), which has been shown to have good concordance with ultrasound results.[154] In the case where EGA based on ultrasound is not available prior to randomization but is available later, the EGA for randomization will be based on LMP and will be compared to the ultrasound-based EGA to ensure that the proper randomization indicator (6 weeks -18 weeks vs. 19 weeks - 30 weeks) was used.

Treatment and Research Status: The Treatment and Research Status form will be used to assess study candidates' status on study inclusion/exclusion criteria related to OUD treatment (e.g., enrolled in treatment, etc.), plans for delivery (e.g., planned delivery hospital), and research participation. In addition, information regarding pressure to attend treatment, which can be related to substance use outcome, will be assessed.

Medical and Psychiatric History: A site MC will obtain a medical and psychiatric history from the participant covering past and present health conditions to help determine eligibility and to provide baseline information.

Drop-out Risk Assessment: The Drop-out Risk Assessment form, which was used in CTN-0052, will be used to assess study candidates' status on the exclusion criterion of being unlikely to complete the study protocol (e.g., due to relocation from the clinic area, probable incarceration, etc.).

7.6.2 Sample Characteristics

Pregnancy-history characteristics: Pregnancy history (e.g., gravidity, parity, etc.) will be assessed by self-report and medical record data, as available, with medical record data being the preferred data source.

HIV/Hepatitis C status: HIV and Hepatitis C status will be assessed by self-report and medical record data, as available, with medical record data being the preferred data source.

Trauma history: Exposure to traumatic events is elevated in women with SUDs and is associated with worse treatment outcomes.[155] The Trauma History Screen is a brief, self-administered instrument with good reliability and validity.[156, 157] The Trauma History Screen will be used to assess the participant's exposure to 14 traumatic events and the severity and duration of the emotional response to any event experienced.

Psychosocial status: The Trauma History Screen does not assess intimate partner violence, which is fairly prevalent in women with OUD, and, thus, will be supplemented with two items that have been used to assess intimate partner violence in past research.[158] In addition, other characteristics of interest, including living arrangements, pregnancy intention, and number of children and their custody will be assessed.

Maternal depression: The Patient Health Questionnaire-9, will be used to assess for symptoms of major depression.[159] It will be completed following the schedule in ***Table 3***. A qualified mental health professional must assess participants reporting current suicidal intent as specified in the site clinical SOP.

Fagerström Test for Nicotine Dependence: Approximately 90% of pregnant women receiving opioid maintenance treatment smoke cigarettes,[160] which is associated with worse pregnancy outcomes and NAS/NOWS.[121, 122] The Fagerström is a brief self-administered assessment of cigarette use patterns,[161] which yields a single overall dependence score.

The Timeline Followback (TLFB) procedure:[162, 163] will assess the participants' self-reported use of substances for the 30 days before study consent.

Thoughts about Abstinence: The Thoughts about Abstinence assessment,[164] which assesses desire to quit, expected success in quitting and estimated difficulty in avoiding relapse, has been found to be predictive of treatment response in pregnant substance users.[165] It will be completed for alcohol, drugs, and cigarettes following the schedule outlined in ***Table 3.***

Opioid Overdose Risk Survey: This survey assesses factors, including having experienced an opioid overdose, that are associated with an increased risk of experiencing an opioid overdose.[166]

Marijuana Use Assessment: This survey assesses the participant's recreational and medical marijuana use frequency over the past 12 months, including reasons for use (e.g., to address medical/psychological concerns, to replace other substances or medications), method of administration, and perceived harm or benefit associated with use.

7.6.3 General Measures

Treatment Services Review: The addiction-related treatment services received by participants during the prior 28 days will be assessed with the use of the Treatment Services Review, Version 6[167] according to the schedule in ***Table 3***. CTN-0080 will utilize the version of the instrument created for use in CTN.

The Family Psychosocial Screener: A number of psychosocial stressors can impact infant development, including maltreatment, parental mental health, and domestic violence.[168, 169] The Family Psychosocial Screener includes components designed to evaluate parental depression, intimate partner violence, parental history of abuse, and a number of other risk factors for developmental problems.[170] During initial development, it was shown to identify significantly more mothers with low self-esteem, depression, and history of abuse as a child than simply reviewing medical records.[171] The instrument is a self-administered questionnaire that typically takes ≤10 minutes to complete. It will be completed by the individual completing the ASQ-3 at 6 and 12 months.

Pregnancy Test: During the postpartum phase, a urine pregnancy test will be completed prior to medication administration for BUP-XR participants receiving the monthly formulation. Additionally, a urine pregnancy test will be completed if the participant self-reports a new pregnancy.

7.7 Administrative Forms

Locator Form(s): A locator form is used to obtain information to assist in finding participants during treatment and at follow-up. This form collects contact information including the participant's current address, email address, phone numbers, and social media contact information. In order to facilitate locating participants if direct contact efforts are unsuccessful, addresses and phone numbers of family/friends who may know how to reach the participant are collected, as well as additional participant information such as social security number, driver's license number, and other information to aid in searches of public records. This information will be collected as outlined in ***Table 3***. The updating of this form may occur during visits at the clinic or via telemedicine. For visits occurring off-site, research staff will contact the participant before or after the visit to update the form via telemedicine. In the event that the participant does not retain custody of her infant, an additional locator form may be obtained from the guardian. No information from locator forms is used in data analyses.

Study Eligibility: This form, which lists all the study inclusion and exclusion criteria, must be completed for every participant who has signed informed consent and entered the Screening/Baseline phase. Eligibility is assessed on an ongoing basis during the screening phase. This form is to be completed in the data system prior to randomization. Eligible participants will be randomized; ineligible participants will be excluded and deemed screen failures.

Study Completion: This form, which indicates that the participant has formally terminated his/her study involvement, must be completed for every participant who has been randomized into the study. The purpose of the Study Completion Form is to document: 1) the date on which a randomized participant attended her final study visit, 2) whether the participant completed the study or ended study involvement prematurely, and 3) if the participant ended study involvement prematurely, the reason why that occurred. This form also provides a location for the site PI attestation of review of all study data.

Missed Visit and Visit Documentation: This form is designed to capture the reason a study visit was missed, the location(s) in which the visit occurred and if assessments occurred outside of the expected window. For missed visits, once the visit window closes without completion of the visit, this assessment will be completed directly in the electronic data capture system. Completing this form will remove the requirement for all assessments scheduled for that visit. Active tracking and follow-up should be performed for all missed visits. For visits that occur, this assessment will be completed directly in the electronic data capture system once the visit is complete to ensure all locations and out-of-window assessments are documented.

End of Medication: This form is completed for all randomized participants. If a participant permanently discontinues study medication during the trial, the reason(s) for discontinuation will be captured. In cases where participants discontinue study medication to transfer to an alternative treatment (e.g., methadone, extended-release naltrexone, BUP from another provider) a release of information will be obtained to confirm treatment engagement.

Protocol Deviation: This form should be entered into the electronic data capture system whenever a protocol deviation occurs. This form will document a description of the deviation, how it occurred, the corrective action taken to resolve the specific deviation, as well as a description of the plan implemented to prevent future occurrences of similar deviations.

Mental Health Follow-up Assessment: This assessment must be completed by a qualified clinician if the participant endorses suicidality on the PHQ-9 and/or either suicidality or homicidality on the PRISM or has a HADS score of 8 or higher on depression or anxiety subscales. In addition, this form will be completed each time an assessment of suicidality/homicidality occurs due to spontaneous participant report during the study. The completion of the Mental Health Follow-up Assessment form requires direct evaluation of the participant for suicide/homicide risk by a qualified mental health professional according to the site's specific SOP. This evaluation will ideally take place prior to the participant leaving the study visit. For visits held via telemedicine or at an external location, research staff will contact a qualified clinician via phone to coordinate an assessment via telemedicine as quickly as possible; ideally this assessment will occur while the staff and participant are together at the external location.

7.8 Conceptual Model Assessments of the Secondary Objective

Randomized participants will be offered the opportunity to participate in the conceptual model assessment (CMA) sub-study (*see* ***section 8.9***), which will provide the data needed to test the MOMs conceptual models (*see* ***section 4.4***). Participants signing informed consent for the CMA sub-study will ideally provide the measures described below. Several measures involve the collection of blood samples; for all blood samples the level of buprenorphine and its metabolites (norbuprenorphine, buprenorphine-glucuronide, and norbuprenorphine-glucuronide) will be assessed.

1. Maternal C_{min} at study weeks 3 and 5. Participants will be scheduled for their study week 3 and 5 visits at the time corresponding with their C_{min}. For BUP-XR participants, a blood sample will be taken right before the administration of the BUP-XR dose. Participants in the BUP-SL arm will have their blood sample drawn prior to taking their (first) BUP-SL dose for the day. The time and date of the most recent medication dose will be assessed based on dosing record (BUP-XR) or self-report (BUP-SL). As permitted by institutional policies and local regulations, staff may complete these blood draws at a remote laboratory and/or community location; alternately sites may contract for phlebotomy/ laboratory services to perform these draws.

2. Fetal Assessment at ~36 weeks EGA. The peak-trough effects for BUP-SL increase with increasing EGA[24] and, thus, the timing of this assessment will be at approximately 36 weeks EGA; this timing should increase the likelihood of observing BUP effects while helping to ensure that the majority of participants are still pregnant. The fetal assessment, consisting of a non-stress test (NST) and biophysical profile (BPP), will be obtained at approximately C_{max}, which is when the BUP effect on the fetus is most likely to be observed. For BUP-SL participants, the monitoring session will occur approximately 2.5 hours after dosing. For BUP-XR participants, the monitoring session will occur approximately 24 hours after injection.[29] The NST will use Doppler measurement to assess fetal heart function over a continuous observation period of ≥20 minutes.[172] The BPP will use ultrasound imaging over a 30-minute period to determine a total score (range: 0-10) based on five parameters: 1) a reactive NST; 2) two or more gross body movements; 3) one or more episodes of limb or hand flexion and extension (muscle tone); 4) breathing movements lasting at least 20 seconds; and 5) a normal amniotic fluid volume index (5-24 cm for the 4 quadrant total).[172] As noted in ***section 4.4***, the variable of most interest is fetal heart rate variability (FHRV) at ~36 weeks EGA. Fetal heart rate accelerations, a measure of episodic FHRV,[24, 69] will be used as the FHRV measure for MOMs. Jansson et al. recently reported that FHR accelerations at ~36 weeks EGA were significantly greater in number at BUP trough, relative to BUP peak.[24] In a secondary analysis of data from the MOTHER trial, Salisbury et al.[172] reported significantly more FHR accelerations for women maintained on BUP-SL, relative to women maintained on methadone, at EGA ~32 weeks. Information about any action specifically taken as the result of the CMA fetal monitoring will be collected. While some sites may be equipped to perform this assessment in-clinic, it is likely that this assessment may occur at other clinical sites either within the institution or at an external clinical site providing these services.

3. Maternal peak-to-trough fluctuation at ~36 weeks EGA. Two blood samples will be taken from participants at approximately 36 weeks EGA, roughly corresponding to C_{max} and C_{min}. The participants will have been taking BUP for a minimum of approximately 6 weeks and so will have reached steady state. Peak-to-trough fluctuation will be calculated as the ratio of C_{max} to C_{min}. The time and date of the most recent medication dose prior to each blood sample will be assessed based on dosing record (BUP-XR) or self-report (BUP-SL). As permitted by institutional policies and local regulations, staff may complete these blood draws at a remote laboratory and/or community location; alternately sites may contract for phlebotomy/laboratory services to perform these draws.

4. Cord and maternal plasma BUP/BUP-metabolite and cotinine levels. Cord plasma is relatively easy to collect and does not require maternal consent.[13] BUP metabolites in cord blood have been found to be predictive of NAS/NOWS severity.[82] Cord cotinine levels reflect tobacco exposure stemming from both first and secondhand smoke

exposure[173] and will be used as the prenatal nicotine exposure covariate for CMA analyses if available. Maternal plasma will be obtained to evaluate the association between maternal BUP/BUP-metabolite levels and NOWS-related outcomes. These samples will be collected at the delivery hospital.

7.9 Infant Neurodevelopmental Outcomes of the Quaternary Objective

Infant caregivers will be offered the opportunity to participate in the infant neurodevelopmental outcomes (INO) sub-study (*see **section 8.10***). Individuals signing consent will participate in the assessments listed below. In addition, a locator form (see ***section 7.7***) will be completed at the 12-month assessment to facilitate retention at the 24-month assessment.

<u>Demographics</u>. The demographics questions from the PhenX toolkit will be utilized.

<u>Bayley™-4</u>. The Bayley™-4[102] is considered the gold standard assessment of early child development and includes cognitive, language, fine motor, and gross motor subscales. The Bayley™-4 will be used to evaluate infants at the 12- and 24-month assessments by a certified examiner who is blind to the CTN-0080 participant's treatment arm. The main outcome is the score on the cognitive subscale at the 24-month assessment. While some sites may be equipped to perform this assessment in-clinic, it is it is likely that this assessment may occur at other clinical sites either within the institution or at an external clinical site providing these services. As determined by the provider, it is permissible for this assessment to be administered as a home visit.

<u>Child Behavior Checklist (CBCL)</u>. The CBCL[174] is completed by caregivers and includes items that describe behavioral, emotional, and social problems that characterize preschool children. The CBCL is the measure used by the National Institute of Child Health and Human Development Neonatal Research Network to assess problem behaviors at the 22-26-month visit and will be collected at the 24-month assessment in the INO. Flexibility in the collection of this assessment is allowed including collection via mail.

8.0 STUDY PROCEDURES

8.1 Study Overview

Many of the CTN-0080 outcomes will be abstracted from medical records. For each participant, these records will be obtained from several institutions including, but not limited to: 1) the study site's treatment program, 2) the participant's OB provider, and 3) the participant's delivery hospital. Releases to access these records will be obtained as outlined in ***Table 3*** and will be used to access the medical records for all participants, including study dropouts, unless the participant explicitly withdraws consent. To streamline data collection, the medical records will serve as source documents, with research staff transcribing the required information onto study case report forms (CRFs). The schedule of research visits and research assessments for all participants are delineated in ***Table 3***. ***Table 5*** provides an overview of the CMA sub-study procedures and assessments. ***Table 6*** provides an overview of the INO sub-study procedures and assessments. While ideally all main study visits will occur at the participating clinic, alternate arrangements may be made at the site's discretion to accommodate participant needs and safety concerns. In accordance with the site's institutional policies and procedures, study visits may occur at other locations affiliated with the institution or elsewhere in the community (including, but not limited to, home visits or visits at non-affiliated community healthcare/laboratory sites), or via remote contact, such as by telephone or other institutionally-approved telemedicine mechanisms. Some assessments may be completed remotely and mailed/shipped to the study team; still others may involve allowing the participant to access the participant self-report data collection system remotely on non-study specific electronic devices using a secure login. CMA and INO procedures may also occur at other locations to accommodate participant and/or provider needs and preferences. All visits occurring outside of the primary research study site will be managed in such a way that there is no increased risk to participant safety.

8.2 Participant Recruitment, Pre-screening, and Consent

Potential participants will be primarily recruited from intakes at participating sites. Advertisements may be used, as needed, but all participants must have completed intake at a study site to be eligible for randomization. Interested candidates will complete a pre-screen, ideally prior to their intake/scheduled clinic visit. The pre-screen will include questions about pregnancy status, past drug use, plans for treatment, and general health status. Candidates who pass the pre-screen will be scheduled for administration of the written consent procedure and a subsequent screening visit. The consent procedure will occur during a live interaction between the candidate and study staff; however, this interaction may occur face-to-face at the study site or at another approved location, or via telemedicine in accordance with institutional policies. If still interested after receiving an explanation of the study, the candidate will be given an opportunity to review, inquire about, and sign the informed consent form. Any participant who has difficulty understanding the information contained in the consent form will be asked to review the misunderstood portion(s) of the consent and discuss them with a research staff member until she shows complete understanding of the information and may thus give full consent. Research staff members will work closely with the study candidates in an effort to help them understand the requirements of their participation. Persons with literacy problems will be assisted to the extent possible. Any participant who is unable to demonstrate understanding of the information contained in the informed consent will be excluded from study participation. In the event that the consent procedure occurs via telemedicine, the candidate will provide consent via a HIPAA-compliant electronic system or will be provided with a prepaid shipper/envelope for

returning the signed consent to the study team. No other study procedures will occur until the signed consent is received back from the candidate.

8.3 Screening/Baseline

After signing the informed consent form, the study participant will proceed through the screening/baseline phase. Ideally, the screening/baseline procedures will be completed within a week, but the allowable time for completion is within 28 days of signing consent.

8.4 Randomization Plan

Eligible participants will be randomized in a 1:1 ratio to BUP-XR or BUP-SL. The randomization process will be performed by computer by the Data and Statistics Center (DSC). A permuted block randomization procedure with random block sizes will be implemented to balance on site, whether participants are on BUP-SL at the time of randomization (yes vs. no), and EGA at time of randomization (6 weeks - 18 weeks vs. 19 weeks - 30 weeks); EGA was used as a stratification variable in the MOTHER trial.[43]

Table 3: Overview of Assessments and Procedures

Perinatal Phase:	Pregnancy					Delivery & Immediate Post-partum	Post-partum												As Needed
	Pre- Screen	Screen/ Base[1]	Random-ization[2]	Week 3	Monthly Post-rand[3]		Month 1	Month 2	Month 3	Month 4	Month 5	Month 6	Month 7	Month 8	Month 9	Month 10	Month 11	Month 12	
**Research Visits**		X	X	X	X		X		X			X			X			X♣	
**Screening Assessments**																			
Pre-screen verbal consent	X																		
Pre-screen interview	X																		
Informed Consent		X																	
Medical record releases		X																	X
Demographics/PhenX Tier 1		X																	
DSM-5 checklist		X																	
PRISM-Suicide/Homicide		X																	
Blood Chemistry[4,5]		X																	
Urine Pregnancy Test		X																	
Estimated Gestational Age [4,5]		X																	
Treatment and Research Status		X																	
Medical and Psych History (Hx)		X																	
Drop-out Risk Assessment		X																	
**Weekly Medication Check Visits[6]**																			
BUP-XR administration/documentation			Weekly administration[6]				Weekly or Monthly administration[7]												
BUP-SL dispense/document; adherence			Weekly - Monthly dispensing[8]				Weekly - Monthly dispensing[8]												
Adverse Events/Serious Adverse Events			Weekly[6]				Weekly[6]												
Injection Site Reaction Reporting Form																			X
Urine drug screen		X	Weekly[6]				Weekly[6]												
Infant sedation assessment							Weekly[6]												
Pregnancy Test (BUP-XR)							Monthly for participants on monthly BUP-XR formulation												X
**Medication Check Visit Compensation**			$20 per visit not corresponding to a research visit																

Table 3 continued on next page

Table 3: Overview of Assessments and Procedures

Perinatal Phase:	Pregnancy					Delivery & Immediate Post-partum	Post-partum												As Needed
	Pre- Screen	Screen/ Base[1]	Random-ization[2]	Week 3	Monthly Post-rand[3]		Month 1	Month 2	Month 3	Month 4	Month 5	Month 6	Month 7	Month 8	Month 9	Month 10	Month 11	Month 12	
**Research Visits**		X	X	X	X		X		X			X			X			X♣	
**Sample Characteristics**																			
Pregnancy Hx, HIV, Hepatitis C		X																	
Trauma History Screen		X																	
Psychosocial status		X																	
Pt. Health Questionnaire-9		X					X											X	
Thoughts about Abstinence		X																	
Timeline Followback (drug/alcohol/tobacco)		X																	
Fagerström		X																	
Opioid Overdose Risk Survey		X																	
Marijuana Use Assessment		X																	
**Safety Assessments**																			
Hospital Anxiety and Depression Scale		X		X	X		X		X			X			X			X	
Prior/Concomitant Meds		X		X	X		X		X			X			X			X	
Fetal Outcomes [4]						X													
Maternal Delivery Outcomes [4]						X													
Birth/Neonatal Outcomes [4]						X													
**Mother Efficacy Assessments**																			
Opioid Craving Scale		X		X	X		X		X			X			X			X	
Adequacy of Prenatal Care Utilization[4]						X													
Opioid Overdose Tracking				X	X		X		X			X			X			X	
SOWS-Gossop		X		X	X		X		X			X			X			X	
**Other Assessments**																			
PROMIS and NMOS		X			X		X		X			X			X			X	
Treatment Services Review		X			X		X		X			X			X			X	
**Maternal Research Visit Compensation**		**$120**		**$40**	**$60**		**$60**		**$60**			**$60**			**$60**			**$70**	

Table 3 continued on next page

Table 3: Overview of Assessments and Procedures

**Perinatal Phase:**	**Pregnancy**					**Delivery & Immediate Post-partum**	**Post-partum**												**As Needed**
	**Pre- Screen**	**Screen/ Base[1]**	**Random- ization[2]**	**Week 3**	**Monthly Post-rand[3]**		**Month 1**	**Month 2**	**Month 3**	**Month 4**	**Month 5**	**Month 6**	**Month 7**	**Month 8**	**Month 9**	**Month 10**	**Month 11**	**Month 12**	
**Research Visits**		X	X	X	X		X		X			X			X			X♣	
**Infant Assessments**																			
NOWS-related and discharge outcomes[4]						X													
ASQ-3[9]												X						X	
Family Psychosocial Screen[9]												X						X	
Medical Record Releases[+]																			X
**Infant assessment compensation**												**$30**						**$30**	
**Administrative Forms**																			
Locator information form(s)[+]	X	X			X		X		X			X			X				X
Study Eligibility			X																
Study Completion																		X	
Missed Visit and Visit Documentation Form		X	X	X	X		X	X	X	X	X	X	X	X	X	X	X	X	
End of Medication																			X
Protocol Deviation																			X
Mental Health Follow-up Assessment																			X

Notes: [1]Can be completed in multiple visits; [2] Randomization will typically occur as part of a screening/baseline visit; [3] Number of visits depends on EGA at randomization and delivery; ♣The 12 month research visit includes the assessment of AEs/SAEs, injection site examination, urine collection, and the infant sedation assessment; [4]completed via medical record abstraction; [5]Will be done for the study if not available from medical record; [6] These visits will occur approximately once per week in accordance with BUP-XR administration windows; [7]Participants who breastfeed will receive the weekly formulation; [8]Dispensing frequency at clinician discretion; [9]May be completed as part of a research visit or outside of a visit (e.g., mailing).[+] are not collected in Advantage eClinical

8.5 Active Treatment Phase

The active treatment phase includes the time during which participants are pregnant (the length of which will depend on the EGA at enrollment and the timing of delivery) through approximately 12 months postpartum. During this time, participants in both treatment conditions will participate in the SUD treatment services typically offered by the site. Participants will receive BUP-XR or BUP-SL as outlined in ***section 9***. Participants in both conditions will meet with study staff in person or remotely to complete study assessments as outlined in ***Table 3***. For participants taking monthly BUP-XR, the last administration/dispensing of the medication will be scheduled for approximately week 49 of the postpartum phase. For participants taking the weekly BUP-XR formulation, the last administration will be scheduled for approximately week 52. Dispensing frequency for BUP-SL (BUP/NX-SL) is at the clinician's discretion. The 12-month research visit will be scheduled for week 53, which will allow the assessment of the participant's medication adherence and any potential adverse events throughout the entire 12-month postpartum period. Participants who are terminating early but are willing to attend an additional visit should complete the assessments that would have been completed at the next scheduled research visit.

8.6 Participant Reimbursement

Participants will be reimbursed for their transportation, inconvenience, and time. The form of this reimbursement will be determined by the study sites. As noted in ***Table 3***, the number of visits during pregnancy will depend on the EGA at randomization and the timing of delivery. The number of weekly Medication Check Visits that do not coincide with a research visit and, thus, for which participants will be reimbursed to help cover transportation costs, are estimated to range from 7-36 visits. The estimated potential range of research monthly visits is 2-8. The recommended reimbursement schedule for participant visits and completion of infant-related assessments is provided in ***Tables 4a*** and ***4b***, respectively. The reimbursement amounts listed in Table 4a will be reduced by $10 if the study participant does not travel to attend the visit.

Table 4a: Reimbursement schedule for participant research visits

Visit	Total per Visit ($)	Total # of Visits	Grand Totals ($)
Screening/baseline			$120
Week 3 visit	$40	1	$40
Weekly Medication Check visits during pregnancy*	$20	7 - 36	$140 - $720
Monthly Research visits during pregnancy	$60	2 - 8	$120 - $480
Weekly Medication Check visits postpartum*	$20	48 - 52	$960 - $1040
Postpartum Research visits (1, 3, 6, 9 months)	$60	4	$240
Postpartum visit month 12	$70	1	$70
Total			$1,690 - $2,710

*Not coinciding with a research visit

Table 4b: Reimbursement schedule for infant-related assessments

Visit	Total per Visit ($)	Total # of Assessment Points	Grand Totals ($)
6-month postpartum assessments	$30	1	$30
12-month postpartum assessments	$30	1	$30
Total			$60

8.7 Medication and Trial Discontinuation

8.7.1 Medication Discontinuation

An investigator may discontinue a participant's medication if he or she deems it clinically appropriate or, at the discretion of the investigator, for any of the reasons listed below.

1. significant side effects that are likely to have been caused by the study medication
2. serious or unexpected AEs which would make further study medication dosing not in the participant's best interest
3. inability or unwillingness of the participant to comply with the study protocol
4. serious illness

A participant may discontinue medication anytime she wishes. Although the participant may withdraw entirely from the study whenever she wishes, she will be strongly encouraged to continue attending visits at which safety measures are scheduled to be assessed.

Study participants withdrawn from the protocol secondary to a medical or psychiatric concern will be referred for appropriate treatment. Participants will be asked to sign a general consent for the release of information to the referred health care provider. Study staff may request transportation for emergency treatment of a participant if medically appropriate (e.g., for acutely psychotic or suicidal participants).

8.7.2 Trial Discontinuation

The study sponsor has the right to discontinue the investigation at any time.

8.8 Access to Treatment After Study Completion

Prior to the 12-month postpartum visit, the research staff will make an effort to arrange for continued treatment with BUP as appropriate within the community. Where this is not possible (due to insurance or availability of treatment resources, etc.), alternative treatment referrals (e.g., methadone maintenance, intensive outpatient psychosocial aftercare, etc.) will be made as appropriate. For participants who do not wish to continue, or for whom community resources are not available, the study will provide up to a two-week BUP-SL taper.

8.9 Conceptual Model Assessments (CMA) Sub-study

8.9.1 Overview

Participants will be invited to participate in the CMA sub-study, which will be used to evaluate the MOMs conceptual models. The CMA includes some assessments that can be readily obtained by study site staff or by clinical/laboratory staff hired by the site to perform these blood draws (e.g., blood draws for C_{max} and C_{min}). The completion of the fetal assessment will likely require the study site to establish a collaboration with an OB clinic capable of completing the fetal assessment. The collection of the cord and mother plasma samples at delivery will need to be coordinated with the delivery hospital. While it would be ideal to have all assessments completed for all participants in the CMA sub-study, feasibility issues may make this difficult for some participants. An overview of CMA assessments and procedures is provided in ***Table 5***.

Table 5. Overview of CMA Assessments and Procedures

	Time+	Pregnancy					Delivery
Research Visits:	Scrn	Wk 3	Wk 5	36 Weeks EGA (1)	36 Weeks EGA (2)	36 Weeks EGA (3)	
Informed Consent	15						
Blood collection for C_{min}		10	10			10	
Blood collection for C_{max}					10		
Fetal Assessment (NST, BPP)				70			
Fetal Monitoring Action*							
Maternal blood collection							X
Cord blood collection							X
Total Time (min)	15	10	10	70	10	10	
Compensation	-	$25	$25	$85	$25	$25	

+Estimated time for participants to complete the assessment/procedure; *Any action taken specifically as a result of the CMA fetal monitoring will be documented.

The reimbursement amounts listed in Table 5 will be reduced by $10 if the study participant does not travel to attend the visit.

8.9.2 Recruitment and Consent

Recruitment for the CMA sub-study will ideally be initiated when a potential participant passes pre-screening for MOMs. The first CMA assessment is scheduled to occur at the study week 3 visit and so the consent will, ideally, be discussed during screening/baseline so that interested participants can be scheduled for the study week 3 visit at a time corresponding to their C_{min}. However, a participant can join the CMA sub-study at any time through her delivery.

8.9.3 Visits for the CMA Sub-study

When possible, the CMA visits will occur in conjunction with the research visits (see ***Table 3***). The first two CMA visits, during which blood is collected at C_{min}, are expected to occur in conjunction with the study week 3 and 5 visits. Three separate CMA assessments will be obtained at approximately 36 weeks EGA. One assessment will be completed at sites responsible for completing the fetal assessment, which may be a study site, or may be another clinical site that provides these services. Participants will complete the fetal assessment when they should be at approximately BUP C_{max} (i.e., roughly 24 hours following BUP-XR injection and 2.5 hours following BUP-SL dose). The second assessment is a blood draw for C_{max}; ideally this blood draw will occur concurrently with the fetal assessment, but may occur on a different day within the 36 weeks EGA timeframe. The third assessment is a blood draw which will ideally occur in conjunction with the research or clinical visit closest to 36 weeks EGA and at a time corresponding to BUP C_{min}.

8.10 Infant Neurodevelopmental Outcomes (INO) Sub-study

8.10.1 Overview

Infant caregivers will be offered the opportunity to participate in the infant neurodevelopmental outcomes (INO) sub-study. An overview of assessments and procedures is provided in ***Table 6***.

The INO data will be locked separately from the rest of the CTN-0080 database to allow CTN-0080 database lock following collection of the final (non-INO) CTN-0080 data point.

Table 6. Overview of INO Assessments and Procedures

	12-month assessment [+]	24-month assessment [+]
Informed Consent	15	
Demographics	5	5**
Locator Form	5	
Bayley™-4	90	120
CBCL		10
Total Time (min)	115	130-135**
Compensation	$100	$150

[+] Estimated time for participants to complete the assessment/procedure.
** Demographics will be collected if caregiver changes.

The reimbursement amounts listed in Table 6 will be reduced by $10 if the study participant does not travel to attend the visit.

8.10.2 Recruitment

The participant in the INO sub-study will be the infant's caregiver, which may or may not be the CTN-0080 participant; hence, recruitment for the INO sub-study will typically occur postpartum. Recruitment of infant caregivers who are not the infant's biological mother will include providing information about the INO sub-study in conjunction with the collection of the ASQ-3s.

8.10.3 INO Visits

At the initial study visit, when the infant is approximately 12 months of age, interested caregivers will complete the informed consent process, provide demographic information, including information about socioeconomic status, and will provide locator information to aid retention efforts for the 24-month assessment. The Bayley™-4 will be administered at both visits. While some sites may be equipped to perform the Bayley in-clinic, it is it is likely that this assessment may occur at other clinical sites either within the institution or at an external clinical site providing these services. As determined by the provider, it is permissible for this assessment to be administered as a home visit.

In addition, at the 24-month assessment, the CBCL will be administered to the caregiver. Flexibility in the collection of the CBCL is allowed, including collection via mail.

If the caregiver changes between the 12-month and 24-month time period, demographic information will be collected a second time.

9.0 STUDY MEDICATIONS

At present, states vary in the extent to which they cover the cost of BUP treatment for pregnant and postpartum women, with some states covering the cost through one year postpartum and others covering only pregnancy. CTN-0080 seeks to evaluate the effectiveness of BUP-XR, relative to BUP-SL, under a model in which states would universally cover the cost of BUP treatment through 12 months postpartum. Given the variability in state coverage, this can only be achieved by providing BUP-XR and BUP-SL at no cost to study participants. Medication will be obtained by National Institute on Drug Abuse (NIDA) or a NIDA contractor for distribution to the sites.

9.1 BUP-XR (CAM2038)

BUP-XR comes in several doses in both the once weekly and once every 4-week (monthly) formulations to allow for individualized medication plans (see ***Table 7***). They are small volume injections that come in prefilled syringes with a safety device that can be stored, unrefrigerated, and administered subcutaneously with a thin needle. The target doses will be 24 mg for the weekly formulation and 96 mg for the monthly formulation, but the actual dose may be lower or higher as determined by the prescribing clinician (e.g., based on craving/withdrawal experienced by the participant, etc.).

Table 7. BUP-SL dose and approximate equivalent weekly and monthly BUP-XR injections

BUP-SL	BUP-XR weekly	BUP-XR monthly
≤6 mg	8 mg (0.16 mL)	--
8-10 mg	16 mg (0.32 mL)	64 mg (0.18 mL)
12-16 mg	24 mg (0.48 mL)	96 mg (0.27 mL)
18-24 mg	32 mg (0.64 mL)	128 mg (0.36 mL)

Note: For a BUP-SL dose >24 mg there is no equivalent BUP-XR dose available for CTN-0080.

9.2 BUP-SL

Sites will be provided with the BUP-SL product(s) that they request. Sites requesting the mono-buprenorphine product will be provided with 2 mg and 8 mg buprenorphine tablets. Sites requesting the combination product will be provided with buprenorphine/naloxone film in 4 mg/1mg and 8mg/2 mg buprenorphine/naloxone doses. Sites may request both forms of BUP-SL (e.g., mono-buprenorphine product for use during pregnancy and combination product for use during the postpartum phase). The target dose will be 16 mg daily, which is consistent with SAMHSA's recommended dose during pregnancy,[13] but the actual dose may be lower or higher as determined by the prescribing clinician (e.g., based on craving/withdrawal experienced by the participant, etc.). A dose of up to 32 mg of BUP-SL daily, which is used in clinical practice, is allowed. While the maximum dose of BUP-XR is equivalent to 24 mg of BUP-SL (see ***Table 7***), allowing up to 32 mg in the BUP-SL arm is consistent with utilizing pragmatic study features where feasible. It should be noted that potential participants who are prescribed 32 mg of BUP-SL daily prior to randomization might not be good candidates for the study because of the lack of an equivalent BUP-XR dose.

9.3 Dispensing Study Medication

Study medications will be provided at no cost to the participants. BUP-XR will be administered by study staff with appropriate licensure and training at induction and approximately every week

during pregnancy. During the postpartum phase, women who are breastfeeding will continue receiving BUP-XR weekly while women who are not breastfeeding may receive BUP-XR every four weeks; a possible exception is continued weekly BUP-XR for women who are not breastfeeding but for whom the prescribing clinician determines that 8 mg is the appropriate dose. Participants transitioning to monthly BUP-XR will be given the dose equating to their BUP-XR weekly dose (see ***Table 7***). CAM2038 should be injected slowly into the subcutaneous tissue of the upper arm, abdomen, buttock or thigh. CAM2038 should not be administered in the same location for a minimum of 8 weeks for the weekly injection. No injection site rotation is needed for the monthly injection. BUP-XR must be dispensed/administered by a licensed medical practitioner appropriately trained and authorized to dispense study medications per local regulations. Dispensing frequency for BUP-SL (BUP/NX-SL) is at the clinician's discretion.

Participants who are discontinued, who do not wish to continue, or for whom community resources to continue are not available, the study may dispense sufficient BUP-SL for up to a two-week taper beginning at any time during treatment. All study medications shall be prepared and dispensed by a pharmacist or licensed medical practitioner appropriately trained and authorized to dispense study medications per local regulations.

9.3.1 Induction

Following randomization, participants will be inducted onto their assigned pharmacotherapy. Guidelines for induction are provided in the study operations manual.

BUP-XR assignment: Research suggests that participants already being treated with BUP-SL can safely be given the corresponding dose of BUP-XR (see ***Table 7***).[175] For participants not being treated with BUP-SL, sites will utilize the induction setting (e.g., outpatient, inpatient, or other in-person setting such as home visit or community clinical location) being used by the clinic for BUP-SL. It should be noted that precipitated opioid withdrawal following BUP-XR administration is not expected for several reasons. First, precipitated withdrawal was not observed in the CAM2038 Phase 2[29] or Phase 3[104] trials. In fact, in the Phase 2 trial participants were stabilized inpatient on 30 mg of morphine four times daily by mouth and then inducted onto 24 or 32 mg weekly CAM2038 15 hours after the last morphine dose (as long as the COWS was ≥8) without precipitated withdrawal. In addition, the authors of the Phase 2 trial reported: *"Owing to a protocol error that may have been somewhat serendipitous, some participants received their first CAM2038 injection with little to no evidence of withdrawal (COWS score <8); none experienced precipitated withdrawal"*.[29] Second, the slower time to onset of C_{max} with the injection (24 hours) versus sublingual buprenorphine/naloxone (2.5 hours) theoretically should decrease the risk of precipitated withdrawal from BUP-XR compared to BUP-SL. Thus, site protocols used for induction on BUP-SL should mitigate risk of precipitated withdrawal from CAM2038. Third, sites will be instructed to only administer the injection when the participants are in opioid withdrawal if they are not already taking BUP-SL.

If precipitated withdrawal does occur after CAM2038 administration, no additional buprenorphine should be given since the levels of buprenorphine will be steadily increasing until they reach a maximum concentration at 24 hours post-administration. Symptomatic non-opioid treatments may be offered as needed to treat withdrawal signs and symptoms as clinically indicated at each site. For example, ondansetron may be administered for nausea and vomiting, acetaminophen for aches and pain, hydroxyzine for anxiety/restlessness, loperamide for loose stool/diarrhea and cyclobenzaprine for muscle cramping. Sites may determine which non-opioid as-needed medications they want to administer, and these medications will be captured as concomitant medications. Reassurance and monitoring should continue until the withdrawal dissipates. Each site should clinically determine whether the withdrawal is severe enough to

warrant: 1) a longer visit to allow time for reassessment, fetal monitoring (to assure continued viability of fetus) and reassurance that the withdrawal is dissipating; 2) hospital admission (e.g., if participant's withdrawal is not subsiding and the withdrawal requires more intensive monitoring and treatment) – for example the participant is unable to stop vomiting and needs IV hydration, fetus needs more intensive monitoring, etc.; and/or 3) an unscheduled visit the following day in order to assure that the withdrawal has dissipated and the participant is stable. Each site must develop its own detailed SOP for handling precipitated withdrawal after CAM2038 administration, with the understanding that more buprenorphine should NOT be given within the 24-hour period after the injection was given. Sites may determine on a case-by-case basis if they want to give more buprenorphine after the 24-hour period after consultation with the site medically responsible investigator.

BUP-SL assignment: The BUP-SL induction procedures typically used by the site will be utilized. As each site already has clinical experience inducting patients onto BUP-SL, we will allow each site to develop its own SOP for handling precipitated withdrawal from BUP-SL and to determine if they want to give more buprenorphine in these cases.

All cases of precipitated withdrawal in either study arm will be considered adverse events. All medications given will be captured as concomitant medications. In the event that precipitated withdrawal leads to fetal monitoring, any action taken specifically as a result of the fetal monitoring will be documented.

9.4 Missed Doses

Study participants may miss doses for a number of reasons including incarceration. A participant who discontinues her medication, for any reason, and then returns to the clinic may be continued/re-inducted on the medication to which she was randomized as long as it is clinically appropriate in the judgment of the prescribing clinician.

9.5 Clinical non-response

Participants with a sub-optimal clinical response to their medication, as determined by the prescribing clinician, may be referred for alternative treatment (e.g., methadone maintenance). Such participants will be encouraged to continue participating in the data collection aspects (e.g., research visits, urine collection, infant assessments) of CTN-0080.

9.6 Study Medication Management/Drug Accountability

Study sites are required to observe local, state, and federal regulations regarding receipt, custody, dispensing, and disposition of all study medications. Each site will maintain an adequate supply of unexpired study medications on site.

9.6.1 Storage

Study medication will be stored in compliance with federal, state, and local laws and institutional policy. Study medication will be stored in a secured location under the conditions specified by the investigator's brochure/package inserts and DEA requirements. Temperature logs should show a daily record of medication storage temperature.

9.6.2 Documentation

Upon receipt, the investigator, pharmacist, or authorized designee at each site is responsible for maintaining written inventory of the study medication obtained for the study. Appropriately qualified and trained study personnel maintain accurate and current accounting of all study

medication by utilizing drug accountability records, which are made available for review by study monitors and other appropriate research personnel. Medication accountability records, including perpetual inventory, will document the amount of study medication ordered, received, and medications administered to, dispensed to, and returned by an individual participant. As with all study documentation, medication accountability records must be maintained in accordance with Good Documentation Practices and must be attributable, legible, contemporaneous, original, accurate, and complete.

Accurate drug accountability records:

- Demonstrate that the study medication was dispensed according to the protocol;
- Document receipt of the study medication, date, lot #, expiration date, quantity, and dosage;
- Account for unopened, un-dispensed, unused, returned, waste, or broken medication;
- Dosing logs should record participant ID #, date dispensed, drug name, lot #, and amount dispensed;
- Indicate who dispensed or handled the study medication.

9.6.3 Used/Unused Medication

Study medication returned by a participant may not be re-issued for use. Unused study medication will be returned and logged into a perpetual inventory of study medication returned. Damaged, returned, expired, or unused study medication will be accounted for by the NIDA contract monitor and sent to the study central pharmacy which will arrange with a reverse distributor for eventual destruction. Other ancillary medications obtained for this study will be destroyed on site or sent for destruction per local institutional policies.

9.6.4 Lost Medication

At the discretion of the site study treatment team, very limited replacement of study medications will be permitted.

9.6.5 Medication Packaging

CAM2038 will be supplied in single use injection kits. The injection kit will be labeled for investigational use only.

Each individual package of sublingual medication, both the mono product and the buprenorphine/naloxone product, will contain the study drug information. The lot number, medication expiration date, and storage conditions, as well as manufacturer and distributor information, will be included on the medication. Each package will be labeled for investigational use only.

9.7 Concomitant Medications

Any medication (including prescription, over-the-counter, herbal supplements, and health store products) to be taken during the study ideally should be approved by the Medical Clinician. Participants who initiate treatment for OUD with methadone or extended-release naltrexone will need to be discontinued from study medication but can otherwise continue with study participation. Participants prescribed benzodiazepines or other CNS depressants should be cautioned to use them only as directed by their physician.

10.0 ANALYTICAL PLAN

10.1 Statistical Hypotheses for Primary Objective

10.1.1 Key Hypotheses

It is hypothesized that the BUP-XR, relative to the BUP-SL, group will: 1) not have greater illicit opioid use during pregnancy (primary, non-inferiority); 2) have lower infant NOWS severity (key secondary, superiority); and 3) not have greater postpartum illicit opioid use (key secondary, non-inferiority).

10.1.2 Secondary Hypotheses

It is also hypothesized that the BUP-XR, relative to the BUP-SL, group will have better:

1. Mother outcomes during pregnancy including significantly:
    - greater proportion of BUP adherent days during pregnancy
    - greater proportion of drug-negative urine samples
    - less opioid craving as assessed by the opioid craving scale
    - better prenatal care utilization as assessed with the APNCU index
2. Mother outcomes postpartum including significantly:
    - greater proportion of BUP adherent days
    - greater proportion of drug-negative urine samples
    - fewer opioid overdoses
    - less opioid craving as assessed by the opioid craving scale
3. Infant outcomes including significantly:
    - lower proportion of infants requiring opioid medication
    - shorter infant hospital length of stay
    - lower proportion of infants for whom adjunct medications are used to treat NOWS
    - lower proportion of infants with an ICD-10 code indicative of NOWS
    - larger head circumference
    - greater weight and length
    - greater gestational age at delivery
    - greater proportion of infants scoring above the ASQ-3 cut-off

10.2 Intent-to-Treat Participant Population

The intent-to-treat population is defined as the participants who are randomized to treatment for maternal outcomes, and their offspring for the infant outcomes.

10.3 Analysis Plan

Each primary and secondary efficacy outcome measure will be analyzed for the intent-to-treat (ITT) population.

The primary outcome will be assessed at a 2.5% significance level for non-inferiority, as recommended by the FDA.[176] If the test of BUP-XR being non-inferior to BUP-SL is statistically significant, then the superiority of the extended-release formulation over the sublingual formulation will be evaluated. There is no need to adjust for this second test if the initial test of non-inferiority is statistically significant. The infant key secondary outcome will be analyzed at the 5% significance level for superiority, and will be interpreted cautiously given the lack of adjustment for multiple testing and will use confidence intervals where possible. The other key secondary outcome (illicit opioid abstinence during postpartum) will be assessed at a 2.5% significance level for non-inferiority.

10.3.1 Non-Inferiority Margin

As noted in ***section 4.0***, there is a dearth of research on the treatment of OUD in pregnant women and, thus, no data upon which to base the non-inferiority margin for CTN-0080 is available. However, the OPTIMA study, a current non-inferiority trial comparing BUP/NX-SL to methadone in non-pregnant participants, is utilizing a primary outcome similar to the CTN-0080 primary outcome (i.e., proportion of opioid-free UDSs collected at the time of the weekly medication check with missing UDSs imputed as positive).[177] The non-inferiority margin for the OPTIMA trial is 15%, which was selected based on a literature review and expert input.[177] Given the more vulnerable nature of the CTN-0080 patient population in which illicit opioid use impacts the health of not only the mother but also the infant, we originally selected the more conservative margin of 11% (i.e., $\Delta=0.11$). However, research continues to suggest that OUD in pregnant and postpartum women is a critical issue in rural communities where access to MOUD is limited.[178] The most cited reason for not prescribing buprenorphine by both rural physicians[179] and nurse practitioners/ physician assistants[180] with a buprenorphine waiver is concern about diversion/misuse. Since this concern would be eliminated by BUP-XR and, hence, BUP-XR has the potential to substantially increase MOUD access for pregnant/postpartum women, it was determined that the 11% margin was overly conservative. Hence, the margin was changed to 15% (i.e., $\Delta=0.15$), which takes into account both the greater vulnerability of pregnant women with OUD and the potential of BUP-XR to substantially increase MOUD access, neither of which is present in the OPTIMA trial and which balance out to utilizing the 15% OPTIMA non-inferiority margin. As outlined in section 10.4 (Sample Size Analysis), the change in margin decreased the number of participants needed for an adequately-powered primary outcome to 176. As noted in **section 6.2**, the target sample size is approximately 200 participants, which would provide more power for secondary analyses, but the primary analysis will be adequately powered with 176 participants. This decrease in required sample size increases the feasibility of the MOMs trial, which was initiated at the start of the COVID-19 epidemic, which, in turn, adversely impacted the randomization rate at all of the study sites. Note that this non-inferiority margin will be used for both the primary outcome, as well as the key secondary outcome of postpartum illicit opioid abstinence.

10.3.2 Key Outcomes

***Illicit opioid abstinence during pregnancy*.** As noted previously, this primary outcome measure is based on urine testing. It is operationalized as the percent of expected UDSs that are negative for illicit opioids. One UDS is expected at the time of each weekly Medication Check Visit between randomization and delivery – any missing UDS is imputed as positive (see

section 10.3.5). The number of expected UDSs will be dependent on the length of time between randomization and delivery, thus we operationalize this primary outcome measure as the *percent* of expected UDSs that are illicit opioid-negative.

The non-inferiority design results in the following hypotheses where μ_A is the mean in arm A and Δ the non-inferiority margin:

$$H_0(BUP - XR\ is\ inferior\ to\ BUP - SL): \mu_{XR} - \mu_{SL} \leq -\Delta$$

$$H_1(BUP - XR\ is\ non - inferior\ to\ BUP - SL): \mu_{XR} - \mu_{SL} > -\Delta\ .$$

To evaluate non-inferiority at the 2.5% significance level, the two-sided 95% confidence interval for the treatment effect, $\mu_{XR} - \mu_{SL}$, will be calculated using a mixed effects model where treatment arm is a fixed effect and the three randomization factors (site, EGA and BUP-SL status at randomization) are random effects. If the lower limit of the confidence interval is greater than -0.15 (i.e., Δ=0.15), we will reject the null hypothesis and conclude non-inferiority of BUP-XR to BUP-SL. Only if the null hypothesis is rejected will superiority of BUP-XR to BUP-SL be considered. This will involve examining the two-sided 95% confidence interval for the treatment effect, $\mu_{XR} - \mu_{SL}$. If the lower limit is above zero, then we can conclude that the extended-release formulation is superior to the sublingual formulation. If this order of testing is followed, then there is no need to adjust for multiplicity.[176, 181] Additional covariates for secondary modeling are described in ***section 7.4***. In the event that the distributional assumptions involved in the mixed effects model are not met, which is likely, alternative non-parametric methods will be considered for this modelling such as quantile regression.

<u>Total days of opioid treatment.</u> Total days of infant opioid treatment will be obtained from the medical record. There are extensive covariates to be included in modelling for the infant outcomes (**Table 2**) at the mother-, infant-, and delivery-hospital-level. Explanation for these covariates is provided in ***section 7.4.*** Since this outcome measure is a count variable and there are covariates requiring adjustment, modelling will utilize Poisson regression. The treatment effect will be measured by the regression coefficient for treatment assignment in the model (H_0: β=0; H_1: $\beta\neq$0).

<u>Illicit opioid abstinence during postpartum.</u> The analytic approach will follow the one outlined above for testing the primary outcome.

10.3.3 Secondary Outcomes

For binary secondary outcomes, such as the proportion of infants requiring opioid medication, and the proportion with an ICD-10 code indicating NOWS, logistic regression or Pearson's χ^2 test of association or another appropriate method will be used to assess the relationship with treatment assignment. Continuous outcomes, such as medication adherence, opioid craving during pregnancy and infant weight at delivery, will be modelled using ANOVA or an appropriate alternative (e.g., quantile regression) to evaluate the effectiveness of BUP-XR. Lastly, the number of opioid overdoses can be modeled as a count variable with possibly zero-inflation, thus a method such as Poisson or Zero-Inflated Poisson regression can be implemented.

10.3.4 Safety Analyses

10.3.4.1 *Adverse Events*

For mothers, adverse events (AEs), including serious adverse events (SAEs), will be summarized by system organ class and preferred term using MedDRA (The Medical Dictionary for Regulatory Activities). Adverse events will be presented as the number and proportion of participants experiencing at least one incidence of each event and will be presented overall and by treatment group. Listings of serious adverse events will be provided, sorted by treatment, system organ class, and preferred term. Detail in these listings will include severity, relationship to study drug, and outcome. The number of participants experiencing each type of AE and SAE may be compared between treatment arms using either Fisher's Exact test or Pearson's χ^2 test, as appropriate.

For infants, events will be summarized in a similar way, however, infant death, congenital anomalies in all infants and other infant SAEs in breastfed infants are reportable.

10.3.4.2 *The Hospital Anxiety and Depression Scale (HADS)*

Depression and anxiety scores, as measured by the HADS, will be summarized using descriptive statistics over time by treatment arm. Repeated measures mixed models may be used to compare the treatment groups on the depression and anxiety symptoms from screening/baseline through 12 months postpartum.

10.3.4.3 *Fetus/Delivery-Related Outcomes*

Fetus/delivery-related outcomes will be summarized by treatment arm. Listings may be provided for fetus- or delivery-related outcomes of particular concern and will be broken down by treatment arm.

10.3.4.4 *Birth/Neonatal Outcomes*

Outcomes related to the birth and/or neonate, such as head circumference or the need for resuscitation, will be summarized by treatment arm and listings may be provided for adverse outcomes, as needed.

10.3.5 Missing Data

Key outcomes. Illicit opioid use during pregnancy (primary outcome) and postpartum (key secondary) are based on UDS; missing UDSs will be imputed as illicit opioid-positive. The number of expected UDSs for each participant during pregnancy will depend on the length of time between randomization and delivery. UDSs are expected at the time of each weekly Medication Check Visit, thus a mother who is on study, for example, for 60 days will be expected to have had 8 UDSs. The final key secondary outcome measure, infant days of opioid treatment, is based on medical records and thus missing data should be minimal. It is possible, albeit rare, given the site selection criteria and the plan for study staff to obtain medical releases for multiple facilities, that a mother may deliver at a hospital for which medical records could not be obtained, and in this case the infant days of opioid treatment will be imputed as the worst value observed (i.e., greatest number of days). Another possible source of missing data will be if the pregnancy does not result in live birth. In this very rare case, the infant days of opioid treatment will also be imputed as the greatest number of days amongst the observed infants.

Different imputation methods, such as those loosening the missing at random assumption, may be considered to measure the sensitivity of the study results to these methods of handling missing data. Sensitivity analyses relating to the impact of the COVID-19 pandemic may be considered as well.

10.4 Sample Size Analysis

10.4.1 Parameter Selection for Calculations

The power analyses required BUP-SL group estimates for the mean and variance of the percent of UDS that are illicit opioid-negative during pregnancy. The mean selected for the sample size simulations was based on a study by Fischer and colleagues[182] which found that the median percentage of urine samples negative for illicit opioids during the entire course of pregnancy was 65% for BUP-SL participants.

10.4.2 Results

Approach. Given a non-inferiority margin of 0.15, the objective of the sample size simulations was to determine whether there would be sufficient power (i.e., ≥80%) to detect that margin with a sample size of 200 participants under different assumptions regarding the impact of the three factors used in randomization. There must be sufficient power if the primary analytic model includes the randomization factors as covariates even if they are not associated with outcome. All simulated data assumed 1:1 random allocation between the treatment arms and thus one half in the BUP-XR arm and one half in the BUP-SL arm.

A simulation study was conducted to assess the power. The details of the simulation will be included in the standalone Statistical Analysis Plan. The mean in the BUP-SL arm was assumed to be 0.65 (***section 10.4.1***), and the mean in the BUP-XR arm was assumed to be 0.50 under the null, which corresponds to the specific non-inferiority margin of 0.15, and 0.65 under the alternative. The variance in both arms was fixed at 0.11, with contributions coming from the three random effects capturing randomization strata/factors and additional random error.

Results. The results of the simulations evaluating the power and type I error for the conservative case where all variance is due to random error and none due to the three random effects is given in **Table 8**. Even if the randomization factors are not associated with the outcome, there will still be *at least* 80% power to detect a 0.15 non-inferiority margin with a 2.5% significance level and a sample size of 200. Note that there is at least 80% power with a slightly lower sample size of 176 as well, meaning that the primary outcome is adequately powered with a sample size of 176.

Table 8. Power for a non-inferiority margin of 15% and a significance level of 2.5%.

Proportion of Variance due to Random Error	Mean in BUP-XR Arm	Power (%)	
		N = 176	N = 200
100%	0.50	2.7%	2.4%
	0.65	84.9%	89.0%

10.5 Descriptive Statistics

Summaries of the characteristics of the participant population in both treatment arms at screening/baseline will be prepared for the intent-to-treat participants. A summary will be prepared to show dropouts/retention over time in each treatment group. The number of missing observations will be compared between treatments.

10.6 Interim Analyses

An interim check of the parameters contributing to the initial power calculations for the BUP-SL group (mean and standard deviation for the percent opioid-negative UDS during pregnancy) will be conducted by the centralized independent DSC. The required sample size to achieve at least 80% power with a margin of 0.15 and a 2.5% type I error rate will be re-calculated. The Data and Safety Monitoring Board (DSMB) will assess whether to continue the trial with or without modification. This interim re-estimation will be implemented since these parameters are being estimated from limited repeated measures data and involve several key assumptions that impact the detectable effect size. The analysis will be conducted when approximately 100 participants (about 50 in the sublingual arm) have been randomized and have primary outcome data available (i.e., completed pregnancy portion of the active treatment phase).

A DSMB will monitor the progress of the present trial. An interim analysis could be performed to assess efficacy, futility, or safety if requested by the DSMB or NIDA. Trial monitoring guidelines for early stopping based on overwhelming benefit might be based on the Lan-DeMets approach to group sequential testing[183] with an O'Brien-Fleming-type boundary.[184] Such an approach does not require the number of interim looks, if any, to be specific *a priori*. The monitoring guidance for early stopping for lack of benefit or for futility might be based upon an approach of conditional probability.[185] Additional safety interim looks could be performed (without formal testing being performed) at the DSMB's or NIDA's request.

10.7 Minority/Sex Analyses

In accordance with National Institutes of Health guidelines, modelling of the key outcomes will be completed to determine whether treatment response was significantly affected by participant minority/sex status using an interaction term between treatment arm and demographic factor being considered. The sex analyses will be completed for the infants. The minority analyses will be conducted for both the mother and infant outcomes.

10.8 Conceptual Model Analyses

Structural equation modeling[186] will be used to test the conceptual models described in ***section 4.4***.

10.9 Health Economic Analytic Plan

A tertiary objective of MOMs is to conduct a health economic analysis comparing the costs and benefits of BUP-XR and BUP-SL; this section describes the analyses to be conducted.

10.9.1 Overview

The economic analyses will be conducted using well-established guidelines,[132, 187, 188] from the perspective of the healthcare sector, as per the recommendation of the Second Panel on Cost Effectiveness in Health and Medicine.[132] The healthcare sector perspective includes all formal (medical) costs incurred by the system on behalf of participants in each arm, and their infants. First, we will determine and value the resources required to start up and manage each treatment strategy on a continuing basis, by site. Next, we will evaluate whether BUP-XR for OUD treatment of pregnant women is associated with more primary, obstetrical, and behavioral healthcare services but fewer emergency and inpatient services; enhanced participant wellbeing; and economic viability, compared to BUP-SL.

The data sources defined in ***section 7.5*** will be used to capture all available healthcare service utilization for participants in both arms over the entire study period (i.e., pregnancy-delivery and

postpartum). The resource costing method will be used to estimate participant-level costs. This method is a powerful tool in a clinical trial environment; it involves determining a price weight for each resource unit consumed and multiplying price weights by units of service.[132, 188, 189] Unit costs will be derived from sources reflecting national real-world costs faced by the healthcare sector. The U.S. Department of Veterans Affairs Federal Supply Schedule will be used to value all medications, as per the current guidelines.[132] Medicare fee-for-service payments will be used to value other healthcare resources, as these payments are designed to reimburse providers for the resources that would be used to treat a typical patient with a given condition and are adjusted for relevant factors that are unique to the patient or provider,[190] as opposed to also including a component for profit and risk adjustment.[132]

To begin, we will conduct bivariate analyses (t-tests for continuous measures and chi-square tests for categorical measures; nonparametric tests will be used for measures that fail to satisfy the assumptions of parametric tests) to test for baseline differences between arms for factors relevant to the economic analysis that may not have been considered in the randomization process (e.g., HRQoL). All data will be analyzed under an intent-to-treat principle. We will model the person period monthly during the pregnancy and delivery phase, and every other month during the 12-month postpartum phase. All analyses will be conducted using a multivariable Generalized Linear Mixed Model (GLMM). The GLMM is an extension of the GLM that allows for the inclusion of random effects. The multivariable aspect of the model is crucial as it allows for the control of factors that are unbalanced between arms because they were not accounted for in the randomization process or may have become unbalanced due to loss to follow-up. The GLMM allows one to choose the most appropriate mean and variance functions according to the fit of the data, and uses all available data for each participant, regardless of whether or not it is complete, making it an ideal statistical procedure for intent-to-treat approaches.[188] Given the differences in mechanisms to generate data, separate multivariable GLMMs will be estimated to predict the mean value for each resource and outcome, at each time period, by study arm. The statistical method of recycled predictions will be used to obtain the final predicted mean values, which will be summed and tested over the pregnancy and delivery phase, and over the entire study period.[188] To account for sampling uncertainty in point estimates, the p-values and standard errors will be estimated using nonparametric bootstrapping techniques within the multivariable framework. All monetary values will be adjusted for inflation. Follow-up measurements obtained beyond 12 months of baseline will be discounted for time preference using the recommended rate of 3%.[132, 188] Our team has extensive experience applying these methods.[138-141]

10.9.2 Intervention Costs

The resources used to implement and administer each treatment strategy, by site, will be estimated using a combination of macro- and micro-costing analyses. To the extent possible, costs will be estimated using a macro-costing (“top down”) approach based on information obtained from the PAASA (see ***section 7.4***). These data may be supplemented with data collected during semi-structured interviews with site clinical leaders, that will allow us to assign individual resources (time and materials) used to deliver the intervention using micro-costing (“bottom up”) techniques.

10.9.3 Healthcare Service Utilization

The analytic methods described in ***section 10.9.1*** will be used to test for differences in utilization of healthcare services, as well as their associated costs, at baseline and over time.

10.9.4 QALYs

We will test for differences in QALYs gained between study arms over the pregnancy and delivery phase, and over the entire study period. A multivariable GLMM regression will be used to estimate the predicted health utility index value for each arm at each time period. The predicted health utility index value will be used to weight the amount of time associated with the arm's typical health state, and mean QALYs gained over time for each arm will be estimated using the area under the curve methodology.[132, 188, 191]

10.9.5 ICERs and Cost-Effectiveness

The costs calculated in ***sections 10.9.2*** and ***10.9.3*** will be combined and the difference between the two arms will be calculated to form the numerator of the ICER (see ***section 7.5***). Four ICERs will be calculated, since we are evaluating two measures of effectiveness (QALYs; Abstinent Years) over two time periods of interest (pregnancy and delivery; entire study period). ICER confidence intervals will be estimated using nonparametric bootstrapping techniques within the multivariable framework. Parametric methods based on parameters obtained from bootstrapping will be used to estimate acceptability curves, which will illustrate the probability that the BUP-XR is a good value relative to BUP-SL for different willingness-to-pay thresholds (i.e., cost-per-QALY and cost-per-Abstinent-Year). ICERs will be calculated and acceptability curves will be constructed regardless of the statistical significance for individual cost and effectiveness differences, as the power to detect a difference in costs and effects jointly exceeds the power to do so individually.[188]

10.9.6 Missing Health Economic Data

To help address censored data we will model the monthly person period and estimate all multivariable regressions using the GLMM, which uses all available data for each participant, regardless of whether the data are complete.[188] In all cases we will diagnose the mechanism of censoring or missingness (i.e., missing completely at random (MCAR), missing at random (MAR), or missing not at random (MNAR)). Logistic regression models predicting missingness, based on participant characteristics and prior measures, will be used to determine whether the data are MCAR and thus ignorable.[192] If not, we will address the missingness using the most appropriate method.[188] We will also conduct extensive sensitivity analyses, including MNAR approaches, to examine the robustness of our outcomes under varying assumptions.[193]

10.9.7 Sensitivity Analyses

Sensitivity analyses will be performed to account for uncertain precision in assumptions and parameter estimates applied in the analyses.[132] For example, we will test the robustness of the results as they pertain to variations in the unit cost estimates, and in the cost of implementing and managing each treatment strategy. Also, values estimated using the more robust and efficient GLMM regression will be compared to those estimated using the more transparent ordinary least squares regression, as well as to the unadjusted mean values.

10.10 Neurodevelopmental Outcomes Analysis

A quaternary objective is to evaluate the impact of BUP-XR, relative to BUP-SL, on neurodevelopment when the infant/child is approximately 12 and 24 months of age. The main outcome is the score on the cognitive subscale of the Bayley™-4 at 24 months of age. The primary method of analysis will be a t-test. For modelling purposes, a mixed model may be used to account for the repeated measures nature of the data and potential covariates, or an

alternative approach that allows for multiple outcome measures per infant/child and construction of a contrast to test the treatment effect at 24 months of age.

10.11 Post-hoc Analyses

In addition to the analyses described above, a number of post-hoc analyses will be completed. Some examples of possible analyses include an exploration of participant screening/baseline variables that are predictive of treatment outcome, and site characteristics associated with treatment outcome.

11.0 REGULATORY COMPLIANCE, REPORTING, AND MONITORING

11.1 Regulatory Compliance

Written approval for the study protocol, consent forms, other supporting documents, and any advertising for participant recruitment will be provided to the sites by the Institutional Review Board (IRB) of record prior to participation in the study. Any amendments to the protocol or consent materials must be approved by the IRB of record before they are implemented. Unanticipated problems involving risk to study participants will be promptly reported to and reviewed by the IRB of record, according to its usual procedures. Annual progress reports and local SAE reports will be submitted to the IRB, according to its usual procedures.

The study will be registered and updated as needed in www.ClinicalTrials.gov.

11.2 Statement of Compliance

This study will be conducted in accordance with the current version of the protocol, in full conformity with the ethical principles outlined in the Declaration of Helsinki, the Regulations for the Protection of Human Subjects codified in the International Council for Harmonisation GCP Guidelines, and all other applicable regulatory requirements. An Operations Manual will be provided as a reference guide and study quality assurance tool.

11.3 Institutional Review Board Approval

Per NOT-OD-16-094, the University of Cincinnati IRB (UC IRB) will be the IRB of record for the protocol and will provide study oversight in accordance with 45 CFR 46. Participating institutions will be asked to agree to rely on the UC IRB and will enter into reliance/authorization agreements for Protocol CTN-0080, as needed. The UC IRB will follow written procedures for reporting its findings and actions to appropriate officials at each participating institution.

Prior to initiating the study, the lead team will ensure that the local IRB at each site involved has entered into a reliance/authorization agreement with the IRB of record (UC IRB) and that written IRB approval has been secured from the IRB of record for each site involved. If changes to the study protocol become necessary, protocol amendments will be submitted in writing for approval by the IRB of record prior to implementation. In addition, the IRB of record will approve all consent forms, recruitment materials, any materials given to the participant, and any changes made to these documents throughout study implementation. For changes to the consent form, a decision will be made regarding whether previously consented participants need to be re-consented. IRB continuing review will be performed annually, or at a greater frequency contingent upon the complexity and risk of the study. Each site principal investigator (PI) is responsible for maintaining copies of all current IRB approval notices, IRB-approved consent documents, and approval for all protocol modifications. These materials must be received by the investigator prior to the initiation of research activities at the site and must be available at any time for audit.

11.4 Regulatory Files

Essential documents are those documents which individually and collectively permit evaluation of the conduct of a trial and the quality of the data produced. These documents serve to demonstrate the compliance of the investigator, sponsor, and monitor with the standards of GCP and with all applicable regulatory requirements. The regulatory files should contain all essential documents, other required regulatory documents, study-specific documents, and all important communications. Regulatory files will be checked at each participating site for

compliance prior to study initiation, throughout the study, as well as at study closure. The Clinical Coordinating Center (CCC) will collaborate with the sites to monitor whether all sponsor-required regulatory documents have been uploaded into the Veeva Vault eTMF.

11.5 Research Advisory Panel of California (California sites only)

Prior to initiating the study, the sponsor or designee will obtain written approval from the Research Advisory Panel of California (RAP-C). Any planned research project to be conducted in California requiring the use of a Schedule I or Schedule II Controlled Substance as its main study medication, as well as research for the treatment of controlled substance addiction or abuse utilizing any drug, scheduled or not (SAT), must be submitted to RAP-C for review and approval prior to study start-up. Study approval is based on review of the study protocol, consent form, and other pertinent study documents. Yearly reports will be provided to the RAP-C by the sponsor or designee in order to obtain continuing study approval. Protocol amendments will also be submitted, and it is required that the Panel be notified of any significant study medication related adverse events that may emerge during conduct of the study at the California sites only.

11.6 Informed Consent

The informed consent form is a means of providing information regarding the trial to a prospective participant and allows for an informed decision about participation in the study. Informed consent continues throughout the individual's study participation. Each study site must have the study informed consent approved by the UC IRB. Prior to initial submission to the IRB and with each subsequent consent revision, the consent form(s) must be sent to the Clinical Coordinating Center and the Lead Node to confirm that each consent form contains the required elements of informed consent as delineated in 21 CFR 50.25(a) and CFR 46.116(b), pertinent additional elements detailed in 21 CFR 50.25(b) and 45 CFR 46.116(c), and any applicable Center for Clinical Trials Network (CCTN) requirements. Every study participant is required to sign a valid, IRB-approved, current version of the study informed consent form prior to the initiation of any study related procedures. The site must maintain the original signed informed consent for every participant in a locked, secure location that is in compliance with all applicable IRB and institutional policies and that is accessible for quality assurance and study monitor review. Every study participant should be given a copy of the signed consent form. Additionally, participants at any California sites will be provided with a copy of the California Research Participant's Bill of Rights.

Prior to signing the informed consent form, research staff who are knowledgeable about the study will explain the study to the potential participant and provide the participant with a copy of the consent to read during the consent (or re-consent) process and to keep for reference. All participants will receive a verbal explanation in terms suited to their comprehension of the purposes, procedures, and potential risks of the study and their rights as research participants. Extensive discussion of risks and possible benefits will be provided to the participants. Participants will have the opportunity to carefully review the written consent form and ask questions prior to signing. The participants should have the opportunity to discuss the study with their family and close friends or think about it prior to agreeing to participate. If the participant is interested in participating in the study, a researcher who is authorized by the PI to obtain informed consent and approved by the UC IRB, will review each section of the informed consent form in detail, answer any of the participant's questions, and determine if the participant comprehends the information provided by administering the comprehension tool. The participant, or participant's legally authorized representative, will consent by signing and dating the consent documents. The person obtaining consent will also sign and date the consent

document. The consent must be properly executed and complete to be valid. It is strongly recommended that another research staff member review the consent after it is signed to ensure that the consent is properly executed and complete.

Staff members delegated by the PI to obtain informed consent must be listed on the Delegation of Responsibility and Staff Signature Log. All persons obtaining consent must have completed appropriate GCP and Human Subjects Protection training, as mandated by NIDA standard operating procedures.

The informed consent form must be updated or revised whenever important new safety information is available, or whenever the protocol is amended in a way that may affect a participant's participation in the trial. The rights and welfare of the participants will be protected by emphasizing to them that the quality of their medical care will not be adversely affected if they decline to participate in this study. The participant will be informed that their participation is voluntary, and they may withdraw from the study at any time, for any reason, without penalty. Individuals who refuse to participate or who withdraw from the study will be treated without prejudice.

For this study, there will be up to four written consents: One for the mother and her as-yet-unborn child to participate in the primary study, one for the mother and her as-yet-unborn child to participate in the CMA sub-study, and one for the caregiver and infant to participate in the INO sub-study; the INO sub-study consent must be co-signed by the infant's Legally Authorized Representative (LAR) in the event that the birth mother no longer has legal authority for the infant. A fourth consent, to be signed by the LAR for the infant, will be used in the event that the birth mother no longer has legal authority for the infant during the postpartum phase of the primary study. The study will seek a waiver of consent for the infant's caregiver to provide information about the infant's development and living environment should the mother lose or surrender custody of the child prior to the end of her participation in the study.

11.7 Participant and Data Confidentiality

Confidentiality will be maintained in accordance with all applicable federal regulations and/or state/Commonwealth law and regulations. By signing the protocol signature page, the investigator affirms that information furnished to the investigator by NIDA will be maintained in confidence and such information will be divulged to the IRB of record; affiliated institution; and employees only under an appropriate understanding of confidentiality with such board or committee, affiliated institution, and employees.

This study will be covered by a federal Certificate of Confidentiality (CoC), which protects identifiable research information from forced disclosure. This protects participants against disclosure of sensitive information (e.g., drug use). The CoC allows the investigator and others who have access to research records to permanently refuse to disclose identifying information on research participation in any civil, criminal, administrative, legislative, or other proceeding, whether at the federal, state, or local level, excepting certain circumstances.

By protecting researchers and institutions from being compelled to disclose information that would identify research participants, the CoC helps achieve the research objectives and promote participation in studies by helping assure confidentiality and privacy to participants. Participant records will be held confidential by the use of study codes for identifying participants on CRFs, secure storage of any documents that have participant identifiers, secure transport of study documents while performing study visits at any off-site location, and secure computing procedures for entering and transferring electronic data.

11.7.1 Health Insurance Portability and Accountability Act (HIPAA)

Study sites may be required by their institutions to obtain authorization from participants for use of protected health information. Releases of participant identifying information that are permitted by the HIPAA regulations, but which are prohibited by other applicable federal regulations and/or state/Commonwealth law and regulation, are prohibited. Sites will be responsible for communicating with the IRB of record and obtaining the appropriate approvals or waivers to be in regulatory compliance.

11.8 Investigator Assurances

Each site must file (or have previously filed) a Federalwide Assurance with the Department of Health and Human Services (HHS) Office for Human Research Protection setting forth the commitment of the organization to establish appropriate policies and procedures for the protection of human research subjects in alignment with 45 CFR 46, Subpart A, with documentation sent to NIDA or its designee. Research covered by these regulations cannot proceed in any manner prior to NIDA receipt of certification that the research has been reviewed and approved by the IRB provided for in the assurance (45 CFR 46.103). Prior to initiating the study, the PI at each study site will sign a protocol signature page, providing assurances that the study will be performed according to the standards stipulated therein.

11.8.1 Financial Disclosure/Conflict of Interest

All investigators will comply with the requirements of 42 CFR Part 50, Subpart F to ensure that the design, conduct, and reporting of the research will not be biased by any conflicting financial interest. Everyone with decision-making responsibilities regarding the protocol will confirm to the sponsor annually that they have met their institutional financial disclosure requirements.

11.8.2 DEA Registration

All Drug Enforcement Agency (DEA) requirements must be met, including registration, inspection if required, and certification, as applicable. In order to receive shipments of study drug, sites must have a DEA registration (facility research registration or a practitioner registration) that has the address where study drug will be shipped on the registration. Additionally, dispensing any controlled substance requires a DEA registration unless exempt by federal or state law or pursuant to CFR Sections 1301.22-1301.26. Sites will follow local DEA guidance regarding the movement of controlled study medications between the registered site and off-site study visit locations.

11.8.3 Investigational New Drug (IND) Requirements

An IND application has been accepted by the FDA for this study (IND# 140724). Any subsequent amendments to this clinical trial submitted to the FDA for review will reflect awareness of and compliance with U.S. Code of Federal Regulations 21 CFR 312 and its subparts, as well as the International Council on Harmonisation GCP Guidelines (ICH E6 R2). This IND study will also be conducted in accordance with all applicable FDA regulations and will comply with all applicable laws and regulations at clinical research sites.

11.9 Quality Assurance Monitoring

In accordance with federal regulations, the study sponsor is responsible for ensuring proper monitoring of a clinical trial and ensuring that the trial is conducted in accordance with the protocol. Qualified monitors will oversee aspects of site conformity to make certain the site staff is operating within the confines of the protocol, and in accordance with GCP. This includes but

is not limited to protocol compliance, documentation auditing, monitoring of drug disposition, and evaluating whether the informed consent process is being correctly followed and documented. Non-conformity with protocol and federal regulations can be reported as a protocol deviation and submitted to the study sponsor and study IRB for further review.

Investigators will host periodic visits by NIDA contract monitors who will ensure all study procedures are conducted, site and study SOPs are followed, and that study data are generated, documented and reported in compliance with the protocol, GCP, and applicable regulations. These monitors will audit, at mutually agreed upon times, regulatory documents, CRFs, and corresponding source documents for each participant. Monitors will have the opportunity and ability to review any study-associated document or file.

NIDA-contracted monitors will assess whether submitted data are accurate and in agreement with source documentation and will also review regulatory/essential documents such as correspondence with the IRB. Areas of particular concern will be participant informed consent forms, protocol adherence, reported safety events and corresponding assessments, and PI oversight and involvement in the trial. Reports will be prepared following the visit and forwarded to the site PI, the Lead Investigator (LI), and NIDA Center for Clinical Trials Network (CCTN).

Qualified node personnel (Node Protocol Managers and/or Quality Assurance (QA) monitors) or other designated party(ies) will provide site management for each site during the trial. Node QA personnel or other designated party(ies) will audit source documentation, including informed consent forms and HIPAA forms. This will take place as specified by the local protocol team, node PI or lead team and will occur as often as needed to help prevent, detect, and correct problems at the study sites. Node QA personnel will verify that study procedures are properly followed and that site staffs are trained and able to conduct the protocol appropriately. If the node staff's review of study documentation indicates that additional training of study personnel is needed, node staff will undertake or arrange for that training. Details of the contract, node QA and data monitoring are found in the study QA monitoring plan.

11.10 Prisoner Certification

As per 45 CFR 46 Subpart C, there are additional protections pertaining to prisoners as study participants. A prisoner is defined as any individual involuntarily confined or detained in a penal institution. The term is intended to encompass individuals sentenced to such an institution under a criminal or civil statute, individuals detained in other facilities by virtue of statutes or commitment procedures which provide alternatives to criminal prosecution or incarceration in a penal institution, and individuals detained pending arraignment, trial, or sentencing. In order to meet these additional protections, the study team will obtain certification from the Office for Human Research Protections (OHRP) to follow-up with participants who become prisoners during the course of the study, as necessary. For individuals who are, or become during the course of their participation, prisoners, study participation will have no effect on the participant's criminal case, release or parole from jail or prison, or probation case.

11.11 Protections for Pregnant Women, Fetuses, and Neonates

As per 45 CFR 46 Subpart B, there are additional protections pertaining to pregnant women, fetuses, and neonates involved in research. Pregnancy encompasses the period of time from implantation until delivery. Fetus is defined as the product of conception from implantation until delivery. In order to meet these additional protections, study staff will abide by conditions outlined in 45 CFR 46.201-206. Potential participants will be fully informed regarding the reasonably foreseeable impact of the research on the fetus or neonate.

11.12 Records Retention and Requirements

Research records for all study participants (e.g., CRFs, source documents, signed consent forms, audio and video recordings, and regulatory files) are to be maintained by the investigator in a secure location for a minimum of 3 years after the study is completed and closed. These records are also to be maintained in compliance with IRB, state and federal requirements, whichever is longest. The sponsor and LI must be notified in writing and acknowledgment must be received by the site prior to the destruction or relocation of research records.

11.13 Reporting to Sponsor

The site PIs agree to submit accurate, complete, legible and timely reports to the Sponsor, as required. These include, but are not limited to, reports of any changes that significantly affect the conduct or outcome of the trial or increase risk to study participants. Safety reporting will occur as described in ***section 11.17*** and the ***safety appendix***. At the completion of the trial, the LI will provide a final report to the Sponsor.

11.14 Audits

The Sponsor has an obligation to ensure that this trial is conducted according to GCP guidelines and may perform quality assurance audits for protocol compliance. The LI and authorized staff from the Ohio Valley Node; the National Institute on Drug Abuse Clinical Trials Network (NIDA CTN, the study sponsor); NIDA's contracted agents, monitors or auditors; monitors from the site's local Node, and other agencies such as the HHS, the OHRP and the IRB of record or the FDA may inspect research records for verification of data, compliance with federal guidelines on human participant research, and to assess participant safety.

11.15 Study Documentation

Each participating site will maintain appropriate study documentation (including medical and research records) for this trial, in compliance with ICH E6 and regulatory and institutional requirements for the protection of confidentiality of participants. Study documentation includes all data-related forms, workbooks, source documents, monitoring logs and appointment schedules; sponsor-investigator correspondence, and signed protocol and amendments; IRB correspondence; and approved consent form and signed participant consent forms. As part of participating in a NIDA-sponsored study, each site will permit authorized representatives from NIDA, NIDA's contracted agents, monitors, or auditors, monitors from the site's local Node, and regulatory agencies to examine (and when permitted by law, to copy) clinical records for the purposes of quality assurance reviews, audits, and evaluation of the study safety, progress, and data validity.

Source documents include <u>all</u> recordings of observations or notations of clinical activities and all reports and records necessary for the evaluation and reconstruction of the clinical research study. Whenever possible, the original recording of an observation should be retained as the source document; however, a photocopy is acceptable provided that it is a clear, legible, and exact duplication of the original document. If the original recording of an observation is the electronic record, that will be considered the source.

11.16 Protocol Deviations

Any departure from protocol-specified procedures and requirements will be classified as either a major or minor protocol deviation. The difference between a major and minor protocol deviation has to do with the seriousness of the event and the corrective action required. A minor protocol

deviation is considered an action (or inaction) that by itself is not likely to affect the scientific soundness of the investigation or seriously affect the safety, rights, or welfare of a study participant. Major protocol deviations are departures that may compromise the participant safety, participant rights, inclusion/exclusion criteria, or the integrity of study data and could be cause for corrective actions if not rectified or prevented from re-occurrence. Sites will be responsible for developing corrective action plans for both major and minor deviations as appropriate. Those corrective action plans may be reviewed/approved by the Lead Node and the CCC with overall approval by the IRB of record. All protocol deviations will be monitored at each site for (1) significance, (2) frequency, and (3) impact on the study objectives, to ensure that site performance does not compromise the integrity of the trial. Departures from SOPs not detailed within the protocol will not be considered to be protocol deviations.

Protocol deviations will be recorded in the Electronic Data Capture (EDC) system via the Protocol Deviation CRF. The CCC, DSC, and the LI must be contacted immediately if an unqualified or ineligible participant is randomized into the study or if another major protocol deviation occurs.

Each site is responsible for reviewing the IRB of record's definition of a protocol deviation (minor deviation) or violation (major deviation) and understanding which events need to be reported to the IRB of record, and when reporting is to be done. Sites must recognize that the CTN and IRB definition of a reportable event may differ and act accordingly in following all reporting requirements for both entities.

11.17 Safety Monitoring

11.17.1 Data and Safety Monitoring Board (DSMB)

An independent CTN DSMB will examine accumulating data to assure protection of participants' safety while the study's scientific goals are being met. The CTN DSMB is responsible for conducting periodic reviews of accumulating safety and efficacy data. It will determine whether there is support for continuation of the trial, or evidence that study procedures should be changed, or if the trial should be halted, for reasons relating to the safety of the study participants, the efficacy of the treatment under study, or inadequate trial performance (e.g., poor recruitment).

11.17.2 Adverse Events (AEs)

The Site PI may appoint one or more Site Physicians/Medical Clinicians (MD, DO, or other medical clinician with DEA waiver or licensure to prescribe/dispense buprenorphine for treatment of opioid use disorder) for this study, who will review or provide consultation for each SAE, as needed. These reviews will include an assessment of the possible relatedness of the event to the study intervention or other study procedures. The Site Physicians/Medical Clinicians will also provide advice for decisions to exclude, refer, or withdraw participants as required. In addition, NIDA will assign a Medical Monitor to this protocol to independently review the safety data, present it to the DSMB for periodic review, and provide PIs a Safety Letter when necessary. The Medical Monitor will determine which safety events require expedited reporting to NIDA, the DSMB, pharmaceutical, and regulatory authorities. This will include events that are serious, related, and unexpected. The study staff will be trained to monitor for and report AEs and SAEs. Additionally, as applicable, sites will submit reporting of AEs/SAEs according to IRB requirements. Each of the sites has established practices for managing medical and psychiatric emergencies, and the study staff will continue to utilize these procedures. Treatment providers

at each site will be responsible for monitoring participants for possible clinical deterioration or other problems, and for implementing appropriate courses of action.

Safety for this study will be monitored through specific study assessments and medical record abstraction in addition to more standard AE/SAE reporting.

<u>The Hospital Anxiety and Depression Scale (HADS):</u> The Hospital Anxiety and Depression Scale (HADS)[117] will be used to assess for symptoms of depression and anxiety. Participants who score in the range for possible depression (total depression score of 8 or higher) or anxiety (total anxiety score of 8 or higher) will be assessed by a qualified clinician before leaving the visit as specified in the site clinical SOP. For visits held via telemedicine or at an external location, research staff will contact a qualified clinician via phone to coordinate an assessment via telemedicine as quickly as possible; ideally this assessment will occur while the staff and participant are together at the external location.

<u>Prior/Concomitant Medications</u>: All medications taken by the participant since the start of her pregnancy and during the active study will be documented on a Prior/Concomitant Medications assessment (see ***Table 3***).

<u>Fetal Outcomes</u>: Gestational age at outcome, spontaneous abortions/miscarriages, stillbirth, pregnancy terminations and indications for termination.

<u>Maternal Delivery Outcomes</u>: Unplanned cesarean section, abnormal fetal presentation during delivery, medical complications at delivery, and analgesic receipt during labor and delivery, postpartum, and upon discharge.

<u>Birth/Neonatal Outcomes</u>: Birth/neonatal outcomes will be abstracted from the medical record. These outcomes include: head circumference, weight and length at birth, gestational age at delivery, and 1-minute and 5-minute Apgar (activity, pulse, grimace, appearance, respiration) scores. Other outcomes will include: major birth defects, stillbirth, neonatal death, and need for resuscitation. Adverse birth outcomes will be characterized, including (if preterm), the reason for preterm birth (e.g., fetal distress, etc.). Other adverse neonatal outcomes will be captured including respiratory distress symptoms, need for respiratory support in the neonatal unit, feeding problems (e.g., need for nasogastric tube), seizures, and other co-morbidities.

<u>Injection Site Examination</u>: Participants will be asked to immediately report any injection site reactions to study staff for evaluation, monitoring, and possible referral, as needed. For weekly injections, the site of the last BUP-XR injection will be examined at the weekly Medication Check Visits. For monthly injections, the BUP-XR injection site will be examined at the weekly Medication Check Visit following the visit during which the injection was given. Injection Site Examinations must be performed in-person. Injection site reactions will be documented on the Injection Site Reaction Reporting form.

<u>Opioid Overdose Tracking</u>: During the study, the participant will complete a self-report assessment about opioid overdose as outlined in ***Table 3***. If a participant reports an overdose, research staff will refer her to the clinic staff who will follow up each case as specified in the site clinical SOP.

<u>Infant Sedation</u>: Infant sedation will be assessed via mother-report as outlined in ***Table 3***. The infant sedation assessment will be completed by participants who are feeding their infants with breastmilk and/or formula and will assess for signs of infant sedation (e.g., not waking for feeding, difficulty breathing, etc.).

<u>Bayley™-4</u>. The Bayley™-4[102] includes cognitive, language, fine motor, and gross motor subscales.

<u>Child Behavior Checklist (CBCL)</u>. The CBCL[174] is administered to caregivers and includes items that describe behavioral, emotional, and social problems that characterize preschool children.

Events related to withdrawal symptoms will be captured on the SOWS-Gossop and will not be duplicate-reported on an AE form. However, precipitated withdrawal during study medication induction will be reported as an AE. Events captured on study specific forms (e.g., HADS) will not be recorded separately as an AE, unless they meet the SAE definition. Any of these events that meet the definition of an SAE, during the reporting periods described below, will be reported on the AE/SAE form set.

Adverse Events and Serious Adverse Events:

For the purpose of this study and if not described above, these events are required to be reported as an AE/SAE:

- All deaths that occur in either the Mother or the Infant (SAE)
- Congenital anomaly observed (SAE)
- Other infant SAEs that occur in breastfed infants
- All maternal AEs/SAEs while on study, except as noted below in “Events that do not require AE/SAE reporting”
- Maternal hospital re-admission within 6 weeks of delivery (SAE)
- Any ICU admission during the Labor and delivery admission (SAE)
- An opioid overdose resulting in naloxone rescue (SAE)

Events that do not require AE/SAE reporting:

- Hospitalization for Labor and Delivery, unless the hospitalization results in an ICU admission
- Prolongation of the hospital stay for a delivery (unless greater than 4 days for vaginal delivery or 7 days for C-section delivery)
- Admission to a hospital for elective surgery or pre-scheduled diagnostic tests
- Opioid overdose, unless resulting in naloxone rescue, hospitalization, or death
- Infant AEs and SAEs, except infant deaths, congenital anomalies in all infants, , and other infant SAEs in breastfed infants

11.17.3 Medical Monitor

The CCC Safety Monitor/Medical Monitor is responsible for reviewing all adverse events and serious adverse events reported. All SAEs will be reviewed within one business day of being reported in Advantage eClinical. The Medical Monitor will also indicate concurrence or not with the details of the report provided by the site PI. Where further information is needed the Safety Monitor/Medical monitor will discuss the event with the site. Reviews of SAEs will be conducted in the Advantage eClinical data system and will be a part of the safety database. All AEs are reviewed on a regular basis to observe trends or unusual events. The CCC Safety Monitor/Medical Monitor will in turn report events to the sponsor and regulatory authorities if the

event meets the definition of an expedited event. Reports will be generated and presented for DSMB meetings.

Standard definitions for adverse events and serious adverse events, their identification, characterization regarding severity and relationship to therapy and processing are described in Appendix A.

11.17.4 Known Potential Toxicities of Study Medication/Intervention

Refer to the investigator’s brochure for CAM2038, and package inserts for buprenorphine, and buprenorphine/naloxone.

11.18 Training Requirements

A comprehensive Training Plan will be developed to incorporate general training, study-specific training, mechanisms for competency assessment as well as a detailed description of training, supervision, and fidelity monitoring procedures. The Investigative Team is responsible for the development of a comprehensive Training Plan, instructional material, and delivery of the training, with the team comprised of the Lead Node, CCC, DSC, as well as other participating nodes and subject matter experts, as applicable.

The CTN-0080 study staff will be trained as specified in the study Training Plan. Training will include Human Subjects Protection and GCP as well as protocol-specific training on assessments, medication management for pharmacological studies, study interventions, safety and safety event reporting, study visits and procedures, data management, quality assurance, laboratory procedures, etc. The Lead Node is primarily responsible for development and delivery of study-specific training related to the study intervention(s) and procedures. The CCC is responsible for the development and delivery of non-intervention training, including regulatory and laboratory procedures, safety and safety event reporting, quality assurance and monitoring, etc. The DSC is responsible for training related to data management, the electronic data capture system, and good data management practices. Other parties will contribute as needed based on the subject matter and material to be covered. The various sub-teams will collaborate to deliver quality instructional material designed to prepare research staff to fully perform study procedures based on the assigned research roles and responsibilities.

In addition to general and study-specific training, the Training Plan will include a description of the delivery methods to be used for each training module (e.g., via self-study, online, webcast, or teleconference). Study staff is required to complete institutionally-required training per their research site, IRB, and authorities with regulatory oversight. Tracking of training completion for individual staff as prescribed for assigned study role(s) will be documented, endorsed by the site PI and the Lead Node, and audited by the CCC. As changes occur in the prescribed training, the Training Plan and training documentation tracking forms will be amended to reflect these adjustments.

12.0 DATA MANAGEMENT AND PROCEDURES

12.1 Design and Development

This protocol will utilize a centralized Data and Statistics Center (DSC) for participant data. The DSC will be responsible for the development of the CRFs, development and validation of the clinical study database, ensuring data integrity, and training site and participating node staff on applicable data management procedures. Ideally, a web-based distributed data entry model will be implemented. This system will be developed to ensure that guidelines and regulations surrounding the use of computerized systems used in clinical trials are upheld. The remainder of this section provides an overview of the data management plan associated with this protocol.

12.1.1 Site Responsibilities

The data management responsibilities of each individual site will be specified by the Lead Node and the DSC.

12.1.2 Data Center Responsibilities

The DSC will: 1) develop and apply a data management plan and will conduct data management activities in accordance with that plan; 2) provide final guided source documents and eCRFs for the collection of all participant data required by the study; 3) develop data dictionaries for each eCRF that will comprehensively define each data element; 4) conduct ongoing data monitoring activities on study data from all participating sites; 5) monitor any preliminary analysis data cleaning activities as needed, and 6) rigorously monitor final study data cleaning.

12.1.3 Data Collection

The data collection process consists of data collected on source documents and entered by the site into eCRFs in Advantage eClinical, direct data entry at the time of visit into Advantage eClinical and upload of specimen results into Advantage eClinical. In the event that Advantage eClinical is not available, the DSC will provide the sites with a final set of guided source documents and completion instructions. Data entry into Advantage eClinical should be completed according to the instructions provided and project specific training. The investigator is responsible for maintaining accurate, complete, and up-to-date records, and for ensuring the completion of the eCRFs for each research participant.

12.2 Data Acquisition and Entry

Completed forms and electronic data should be entered into the data management system in accordance with the CRF Completion Guidelines established by the DSC. Only authorized individuals shall have access to electronic CRFs.

12.3 Data Editing

Data will be entered into the DSC automated data acquisition and management system. Dynamic reports listing missing values and forms are available to sites at all times in Advantage eClinical. These reports will be monitored regularly by the DSC. Additionally, if incomplete or inaccurate data are found, a query will be generated to the sites for a response. Sites will resolve data inconsistencies and errors and enter all corrections and changes into Advantage eClinical.

12.4 Database Transfer/Lock

The DSC will conduct final data quality assurance checks and "lock" the study participant database from further modification at the end of the 12-month postpartum phase and at the end of the INO sub-study. Additionally, the data may be soft locked or frozen at the completion of each of the screening and pregnancy phases. The final analysis dataset will be returned to NIDA, as requested, for storage and archive.

12.5 Data Sharing

Data will be transmitted by the DSC to the designated party for de-identification, posting, storing, and archiving on NIDA's Data Share website. Data Share is an online repository of data from studies funded by the NIDA and is located at: https://datashare.nida.nih.gov/.

12.6 Data Training

The training plan for site staff includes provisions for training on assessments, CRF completion guidelines, and computerized systems.

12.7 Data QA

To address the issue of data quality, the DSC will follow a standard data monitoring plan. An acceptable data quality level prior to any database lock will be given as part of the data management plan. Data quality summaries will be made available during the course of the study.

13.0 PUBLIC ACCESS AND DATA SHARING PLAN

This study will comply with the NIH Data Sharing Policy and Implementation Guidance (https://grants.nih.gov/grants/policy/data_sharing/data_sharing_guidance.htm) and the HEAL Public Access and Data Sharing Policy (https://www.nih.gov/research-training/medical-research-initiatives/heal-initiative/research/heal-public-access-data-sharing-policy). Investigators will also register and report results of the trial in ClinicalTrials.gov, consistent with the requirements of the Policy on the Dissemination of NIH-Funded Clinical Trial Information and the Clinical Trials Registration (https://grants.nih.gov/policy/clinical-trials/reporting/understanding/nih-policy.htm).

Primary data for this study will be available to the public in the NIDA data repository, per NIDA CTN policy. For more details on data sharing please visit https://datashare.nida.nih.gov/.

The primary outcome(s) publication will be included along with study underlying primary data in the data share repository, and it will also be deposited in PubMed Central http://www.pubmedcentral.nih.gov/ per NIH Policy (http://publicaccess.nih.gov/).

Every attempt will be made to publish results in peer-reviewed journals. The planning, preparation, and submission of publications will follow the policies of the Publications Committee of the CTN. Considerations for ensuring confidentiality of any shared data are described in ***section 11.7*****.**

14.0 PROTOCOL SIGNATURE PAGE

SPONSOR'S REPRESENTATIVE (CCTN SCIENTIFIC OFFICER OR DESIGNEE)

Printed Name	Signature	Date

ACKNOWLEDGEMENT BY INVESTIGATOR:

- I am in receipt of version 8.0 of the protocol and agree to conduct this clinical study in accordance with the design and provisions specified therein.
- I agree to follow the protocol as written except in cases where necessary to protect the safety, rights, or welfare of a participant, an alteration is required, and the sponsor and IRB have been notified prior to the action.
- I will ensure that the requirements relating to obtaining informed consent and institutional review board (IRB) review and approval in 45 CFR 46 are met.
- I agree to personally conduct or supervise this investigation at this site and to ensure that all site staff assisting in the conduct of this study are adequately and appropriately trained to implement this version of the protocol and that they are qualified to meet the responsibilities to which they have been assigned.
- I agree to comply with all the applicable federal, state, and local regulations regarding the obligations of clinical investigators as required by the Department of Health and Human Services (HHS), the state, and the IRB.

SITE'S PRINCIPAL INVESTIGATOR

Printed Name	Signature	Date

Clinical Site Name ______________________________

Node Affiliation ______________________________

15.0 REFERENCES

1. Hayes MJ, Brown MS. Epidemic of Prescription Opiate Abuse and Neonatal Abstinence. *Jama-Journal of the American Medical Association.* May 9 2012;307(18):1974-1975.

2. Jones HE, Heil SH, Baewert A, et al. Buprenorphine treatment of opioid-dependent pregnant women: a comprehensive review. *Addiction.* Nov 2012;107:5-27.

3. Maeda A, Bateman BT, Clancy CR, Creanga AA, Leffert LR. Opioid Abuse and Dependence during Pregnancy Temporal Trends and Obstetrical Outcomes. *Anesthesiology.* Dec 2014;121(6):1158-1165.

4. Patrick SW, Schiff DM, Committee On Substance USE, Prevention. A Public Health Response to Opioid Use in Pregnancy. *Pediatrics.* Mar 2017;139(3).

5. Patrick SW, Schumacher RE, Benneyworth BD, Krans EE, McAllister JM, Davis MM. Neonatal abstinence syndrome and associated health care expenditures: United States, 2000-2009. *JAMA.* May 09 2012;307(18):1934-1940.

6. Patrick SW, Davis MM, Lehmann CU, Cooper WO. Increasing incidence and geographic distribution of neonatal abstinence syndrome: United States 2009 to 2012. *J Perinatol.* 2015;35(8):650-655.

7. Kandall SR, Albin S, Gartner LM, Lee KS, Eidelman A, Lowinson J. The narcotic-dependent mother: fetal and neonatal consequences. *Early human development.* Oct 1977;1(2):159-169.

8. Hulse GK, Milne E, English DR, Holman CD. Assessing the relationship between maternal opiate use and antepartum haemorrhage. *Addiction.* Oct 1998;93(10):1553-1558.

9. Peles E, Schreiber S, Bloch M, Dollberg S, Adelson M. Duration of methadone maintenance treatment during pregnancy and pregnancy outcome parameters in women with opiate addiction. *J Addict Med.* Mar 2012;6(1):18-23.

10. Tolia VN, Patrick SW, Bennett MM, et al. Increasing incidence of the neonatal abstinence syndrome in U.S. neonatal ICUs. *N Engl J Med.* May 28 2015;372(22):2118-2126.

11. American College of Obstetricians and Gynecologists. Committee Opinion: Opioid Abuse, Dependence, and Addiction in Pregnancy. 2012.

12. World Health Organization. *Guidelines for identification and management of substance use and substance use disorders in pregnancy.* Geneva, Switzerland: World Health Organization Press; 2014.

13. Substance Abuse and Mental Health Services Administration. Clinical Guidance for Treating Pregnant and Parenting Women With Opioid Use Disorder and Their Infants. Rockville, MD: Department of Health and Human Services; 2018.

14. Reddy UM, Davis JM, Ren Z, Greene MF, Opioid Use in Pregnancy NAS, Childhood Outcomes Workshop Invited S. Opioid Use in Pregnancy, Neonatal Abstinence Syndrome, and Childhood Outcomes: Executive Summary of a Joint Workshop by the Eunice Kennedy Shriver National Institute of Child Health and Human Development, American College of Obstetricians and Gynecologists, American Academy of Pediatrics, Society for Maternal-Fetal Medicine, Centers for Disease Control and Prevention, and the March of Dimes Foundation. *Obstet Gynecol.* Jul 2017;130(1):10-28.

15. Lofwall MR, Walsh SL. A review of buprenorphine diversion and misuse: the current evidence base and experiences from around the world. *J Addict Med.* Sep-Oct 2014;8(5):315-326.

16. Hser YI, Saxon AJ, Huang D, et al. Treatment retention among patients randomized to buprenorphine/naloxone compared to methadone in a multi-site trial. *Addiction.* Jan 2014;109(1):79-87.

17. Pinto H, Maskrey V, Swift L, Rumball D, Wagle A, Holland R. The SUMMIT trial: a field comparison of buprenorphine versus methadone maintenance treatment. *J Subst Abuse Treat.* Dec 2010;39(4):340-352.

18. Gryczynski J, Mitchell SG, Jaffe JH, et al. Retention in methadone and buprenorphine treatment among African Americans. *J Subst Abuse Treat.* Sep 2013;45(3):287-292.

19. Greenwald MK, Comer SD, Fiellin DA. Buprenorphine maintenance and mu-opioid receptor availability in the treatment of opioid use disorder: implications for clinical use and policy. *Drug Alcohol Depend.* Nov 01 2014;144:1-11.

20. Wilder CM, Winhusen T. Pharmacological Management of Opioid Use Disorder in Pregnant Women. *CNS Drugs.* Aug 2015;29(8):625-636.

21. Rizzo RA, Neumann AM, King SO, Hoey RF, Finnell DS, Blondell RD. Parenting and concerns of pregnant women in buprenorphine treatment. *MCN Am J Matern Child Nurs.* Sep-Oct 2014;39(5):319-324.

22. Schiff DM, Nielsen T, Terplan M, et al. Fatal and Nonfatal Overdose Among Pregnant and Postpartum Women in Massachusetts. *Obstetrics and Gynecology.* Aug 2018;132(2):466-474.

23. Wilder C, Lewis D, Winhusen T. Medication assisted treatment discontinuation in pregnant and postpartum women with opioid use disorder. *Drug Alcohol Depend.* Apr 1 2015;149:225-231.

24. Jansson LM, Velez M, McConnell K, et al. Maternal buprenorphine treatment and fetal neurobehavioral development. *Am J Obstet Gynecol.* May 2017;216(5):529 e521-529 e528.

25. Concheiro M, Jones HE, Johnson RE, Choo R, Huestis MA. Preliminary Buprenorphine Sublingual Tablet Pharmacokinetic Data in Plasma, Oral Fluid, and Sweat During Treatment of Opioid-Dependent Pregnant Women. *Therapeutic Drug Monitoring.* Oct 2011;33(5):619-626.

26. Bastian JR, Chen H, Zhang H, et al. Dose-adjusted plasma concentrations of sublingual buprenorphine are lower during than after pregnancy. *Am J Obstet Gynecol.* Jan 2017;216(1):64 e61-64 e67.

27. Greenwald M, Johanson CE, Bueller J, et al. Buprenorphine duration of action: mu-opioid receptor availability and pharmacokinetic and behavioral indices. *Biol Psychiatry.* Jan 1 2007;61(1):101-110.

28. Nasser AF, Heidbreder C, Gomeni R, Fudala PJ, Zheng B, Greenwald MK. A population pharmacokinetic and pharmacodynamic modelling approach to support the clinical development of RBP-6000, a new, subcutaneously injectable, long-acting, sustained-release formulation of buprenorphine, for the treatment of opioid dependence. *Clin Pharmacokinet.* Sep 2014;53(9):813-824.

29. Walsh SL, Comer SD, Lofwall MR, et al. Effect of buprenorphine weekly depot (cam2038) and hydromorphone blockade in individuals with opioid use disorder: A randomized clinical trial. *JAMA Psychiatry.* 2017.

30. Coe MA, Nuzzo PA, Levy-Cooperman N, et al. Weekly CAM2038: Pharmacokinetics and Pharmacokinetic/Pharmacodynamic Evaluation of Opioid Blockade in Humans. *College on Problems of Drug Dependence 79th Annual Scientific Meeting*. Montréal; 2017.

31. Caritis SN, Bastian JR, Zhang H, et al. An evidence-based recommendation to increase the dosing frequency of buprenorphine during pregnancy. *Am J Obstet Gynecol.* Oct 2017;217(4):459 e451-459 e456.

32. Jansson LM, Velez ML, McConnell K, et al. Maternal buprenorphine treatment and infant outcome. *Drug Alcohol Depend.* Nov 1 2017;180:56-61.

33. Velez ML, McConnell K, Spencer N, Montoya L, Tuten M, Jansson LM. Prenatal buprenorphine exposure and neonatal neurobehavioral functioning. *Early human development.* Dec 7 2017;117:7-14.

34. Coleman CI, Limone B, Sobieraj DM, et al. Dosing frequency and medication adherence in chronic disease. *J Manag Care Pharm.* Sep 2012;18(7):527-539.

35. Srivastava K, Arora A, Kataria A, Cappelleri JC, Sadosky A, Peterson AM. Impact of reducing dosing frequency on adherence to oral therapies: a literature review and meta-analysis. *Patient Prefer Adherence.* 2013;7:419-434.

36. Compton WM, Volkow ND. Improving Outcomes for Persons With Opioid Use Disorders: Buprenorphine Implants to Improve Adherence and Access to Care. *JAMA : the journal of the American Medical Association.* Jul 19 2016;316(3):277-279.

37. Iglay K, Cao X, Mavros P, Joshi K, Yu S, Tunceli K. Systematic Literature Review and Meta-analysis of Medication Adherence With Once-weekly Versus Once-daily Therapy. *Clin Ther.* Aug 2015;37(8):1813-1821 e1811.

38. Li X, Shorter D, Kosten TR. Buprenorphine in the treatment of opioid addiction: opportunities, challenges and strategies. *Expert Opin Pharmacother.* Oct 2014;15(15):2263-2275.

39. Terplan M. Beyond the Treatment Box: Perspectives on the Federal Response to Opioid Use, Pregnancy, and Neonatal Abstinence Syndrome. *J Addict Med.* May/Jun 2017;11(3):176-177.

40. Terplan M, McNamara EJ, Chisolm MS. Pregnant and non-pregnant women with substance use disorders: the gap between treatment need and receipt. *J Addict Dis.* 2012;31(4):342-349.

41. Angelotta C, Weiss CJ, Angelotta JW, Friedman RA. A Moral or Medical Problem? The Relationship between Legal Penalties and Treatment Practices for Opioid Use Disorders in Pregnant Women. *Womens Health Issues.* Nov - Dec 2016;26(6):595-601.

42. Braeburn Pharmaceuticals Inc. Slides for the November 1, 2017 Joint Meeting of the Psychopharmacologic Drugs Advisory Committee and the Drug Safety and Risk Management Advisory Committee. https://www.fda.gov/AdvisoryCommittees/CommitteesMeetingMaterials/Drugs/PsychopharmacologicDrugsAdvisoryCommittee/ucm586722.htm. Accessed March 1, 2018.

43. Jones HE, Kaltenbach K, Heil SH, et al. Neonatal Abstinence Syndrome after Methadone or Buprenorphine Exposure. *New England Journal of Medicine.* Dec 9 2010;363(24):2320-2331.

44. Jones HE, Fischer G, Heil SH, et al. Maternal Opioid Treatment: Human Experimental Research (MOTHER)-approach, issues and lessons learned. *Addiction.* Nov 2012;107:28-35.

45. Ford I, Norrie J. Pragmatic Trials. *N Engl J Med.* Aug 4 2016;375(5):454-463.

46. Cheatham CL, Goldman BD, Fischer LM, da Costa KA, Reznick JS, Zeisel SH. Phosphatidylcholine supplementation in pregnant women consuming moderate-choline diets does not enhance infant cognitive function: a randomized, double-blind, placebo-controlled trial. *Am J Clin Nutr.* Dec 2012;96(6):1465-1472.

47. Institute of Medicine Standing Committee on the Scientific Evaluation of Dietary Reference Intakes. Choline. *Dietary Reference Intakes for Thiamin, Riboflavin, Niacin, Vitamin B6, Folate, Vitamin B12, Pantothenic Acid, Biotin, and Choline*. Washington, D.C.: National Academies Press; 1998:390-422.

48. American Medical Association. PolicyFinder: Choline Supplementation in Prenatal Vitamins H-420.951. https://policysearch.ama-assn.org/policyfinder. Accessed January 15, 2019.

49. Ferretti CA, Spotti ML, Di Cosimo JI. Diglyceride-rich oils from glycerolysis of edible vegetable oils. *Catalysis Today.* 2018;302:233-241.

50. Burd L, Blair J, Dropps K. Prenatal alcohol exposure, blood alcohol concentrations and alcohol elimination rates for the mother, fetus and newborn. *J Perinatol.* Sep 2012;32(9):652-659.

51. Kalinowski A, Humphreys K. Governmental standard drink definitions and low-risk alcohol consumption guidelines in 37 countries. *Addiction.* Jul 2016;111(7):1293-1298.

52. Centers for Disease Control and Prevention. Breastfeeding: Alcohol. https://www.cdc.gov/breastfeeding/breastfeeding-special-circumstances/vaccinations-medications-drugs/alcohol.html. Accessed November 13, 2018.

53. Nguyen L, Lander LR, O'Grady KE, et al. Treating women with opioid use disorder during pregnancy in Appalachia: Initial neonatal outcomes following buprenorphine + naloxone exposure. *Am J Addict.* Mar 2018;27(2):92-96.

54. Jumah NA, Edwards C, Balfour-Boehm J, et al. Observational study of the safety of buprenorphine plus naloxone in pregnancy in a rural and remote population. *Bmj Open.* 2016;6(10).

55. Krsak M, Trowbridge P, Regan N, Freedman KI. Buprenorphine with, or without, Naloxone for Pregnant Women?–Review of Current Evidence and Practice in Massachusetts. *Journal of Alcoholism & Drug Dependence.* 2017;05(03).

56. Debelak K, Morrone WR, O'Grady KE, Jones HE. Buprenorphine plus Naloxone in the Treatment of Opioid Dependence during Pregnancy-Initial Patient Care and Outcome Data. *American Journal on Addictions.* May-Jun 2013;22(3):252-254.

57. Dooley J, Gerber-Finn L, Antone I, et al. Buprenorphine-naloxone use in pregnancy for treatment of opioid dependence Retrospective cohort study of 30 patients. *Canadian Family Physician.* Apr 2016;62(4):E194-E200.

58. Wiegand SL, Stringer EM, Stuebe AM, Jones H, Seashore C, Thorp J. Buprenorphine and naloxone compared with methadone treatment in pregnancy. *Obstet Gynecol.* Feb 2015;125(2):363-368.

59. Lofwall M, Nunes E, Bailey G, et al. A Phase III outpatient randomized, double-blind, double-dummy controlled trial evaluating efficacy of CAM2038 (buprenorphine FluidCrystal® injection depot) for opioid use disorder (Manuscript under review.). *College on Problems of Drug Dependence*. Montreal, Canada; 2017.

60. Hall ES, Wexelblatt SL, Crowley M, et al. Implementation of a Neonatal Abstinence Syndrome Weaning Protocol: A Multicenter Cohort Study. *Pediatrics.* Oct 2015;136(4):e803-810.

61. Patrick SW, Schumacher RE, Horbar JD, et al. Improving Care for Neonatal Abstinence Syndrome. *Pediatrics.* May 2016;137(5).

62. Bogen DL, Whalen BL, Kair LR, Vining M, King BA. Wide Variation Found in Care of Opioid-Exposed Newborns. *Acad Pediatr.* May - Jun 2017;17(4):374-380.

63. MacMillan KDL, Rendon CP, Verma K, Riblet N, Washer DB, Volpe Holmes A. Association of Rooming-in With Outcomes for Neonatal Abstinence Syndrome: A Systematic Review and Meta-analysis. *JAMA Pediatr.* Apr 1 2018;172(4):345-351.

64. Grimm D, Pauly E, Poschl J, Linderkamp O, Skopp G. Buprenorphine and norbuprenorphine concentrations in human breast milk samples determined by liquid chromatography-tandem mass spectrometry. *Ther Drug Monit.* Aug 2005;27(4):526-530.

65. Lindemalm S, Nydert P, Svensson JO, Stahle L, Sarman I. Transfer of buprenorphine into breast milk and calculation of infant drug dose. *J Hum Lact.* May 2009;25(2):199-205.

66. Ilett KF, Hackett LP, Gower S, Doherty DA, Hamilton D, Bartu AE. Estimated dose exposure of the neonate to buprenorphine and its metabolite norbuprenorphine via breastmilk during maternal buprenorphine substitution treatment. *Breastfeed Med.* Aug 2012;7:269-274.

67. Jansson LM, Spencer N, McConnell K, et al. Maternal Buprenorphine Maintenance and Lactation. *J Hum Lact.* Nov 2016;32(4):675-681.

68. DiPietro JA, Costigan KA, Shupe AK, Pressman EK, Johnson TR. Fetal neurobehavioral development: associations with socioeconomic class and fetal sex. *Dev Psychobiol.* Jul 1998;33(1):79-91.

69. DiPietro JA, Costigan KA, Voegtline KM. Studies in Fetal Behavior: Revisited, Renewed, and Reimagined. *Monogr Soc Res Child Dev.* Sep 2015;80(3):vii;1-94.
70. McCarthy JJ, Leamon MH, Willits NH, Salo R. The effect of methadone dose regimen on neonatal abstinence syndrome. *J Addict Med.* Mar-Apr 2015;9(2):105-110.
71. Squires J, Bricker D. *Ages & Stages Questionnaires®, Third Edition (ASQ- 3TM). A parent-completed child-monitoring system*. Baltimore: Paul H. Brookes Publishing Co.; 2009.
72. Squires J, Bricker D, Potter L. Revision of a parent-completed development screening tool: Ages and Stages Questionnaires. *J Pediatr Psychol.* Jun 1997;22(3):313-328.
73. Singh A, Yeh CJ, Boone Blanchard S. Ages and Stages Questionnaire: a global screening scale. *Bol Med Hosp Infant Mex.* Jan - Feb 2017;74(1):5-12.
74. Siddiqui S, Fifer WP, Ordonez-Retamar M, Nugent JD, Williams IA. An antenatal marker of neurodevelopmental outcomes in infants with congenital heart disease. *Journal of perinatology : official journal of the California Perinatal Association.* Aug 2017;37(8):953-957.
75. DiPietro JA, Bornstein MH, Hahn CS, Costigan K, Achy-Brou A. Fetal heart rate and variability: stability and prediction to developmental outcomes in early childhood. *Child Dev.* Nov-Dec 2007;78(6):1788-1798.
76. Bornstein MH, DiPietro JA, Hahn CS, Painter K, Haynes OM, Costigan KA. Prenatal Cardiac Function and Postnatal Cognitive Development: An Exploratory Study. *Infancy.* 2002;3(4):475-494.
77. Figueiredo B, Pinto TM, Pacheco A, Field T. Fetal heart rate variability mediates prenatal depression effects on neonatal neurobehavioral maturity. *Biol Psychol.* Feb 2017;123:294-301.
78. Merhar SL, McAllister JM, Wedig-Stevie KE, Klein AC, Meinzen-Derr J, Poindexter BB. Retrospective review of neurodevelopmental outcomes in infants treated for neonatal abstinence syndrome. *Journal of perinatology : official journal of the California Perinatal Association.* Mar 7 2018.
79. McGlone L, Mactier H. Infants of opioid-dependent mothers: neurodevelopment at six months. *Early human development.* Jan 2015;91(1):19-21.
80. Desai RJ, Huybrechts KF, Hernandez-Diaz S, et al. Exposure to prescription opioid analgesics in utero and risk of neonatal abstinence syndrome: population based cohort study. *BMJ.* May 14 2015;350:h2102.
81. Kaltenbach K, Holbrook AM, Coyle MG, et al. Predicting treatment for neonatal abstinence syndrome in infants born to women maintained on opioid agonist medication. *Addiction.* Nov 2012;107:45-52.
82. Shah D, Brown S, Hagemeier N, et al. Predictors of neonatal abstinence syndrome in buprenorphine exposed newborn: can cord blood buprenorphine metabolite levels help? *Springerplus.* 2016;5(1):854.
83. Klaman SL, Isaacs K, Leopold A, et al. Treating Women Who Are Pregnant and Parenting for Opioid Use Disorder and the Concurrent Care of Their Infants and Children: Literature Review to Support National Guidance. *J Addict Med.* May/Jun 2017;11(3):178-190.
84. Lewis T, Dinh J, Leeder JS. Genetic determinants of fetal opiate exposure and risk of neonatal abstinence syndrome: Knowledge deficits and prospects for future research. *Clin Pharmacol Ther.* Sep 2015;98(3):309-320.
85. Hytinantti T, Kahila H, Renlund M, Jarvenpaa AL, Halmesmaki E, Kivitie-Kallio S. Neonatal outcome of 58 infants exposed to maternal buprenorphine in utero. *Acta Paediatr.* Aug 2008;97(8):1040-1044.
86. Braeburn Pharmaceuticals Inc. FDA Advisory Committee Meeting Briefing Document: CAM2038 (buprenorphine) subcutaneous injection.

https://www.fda.gov/downloads/AdvisoryCommittees/CommitteesMeetingMaterials/Drugs/PsychopharmacologicDrugsAdvisoryCommittee/UCM582594.pdf. Accessed April 17, 2018.

87. Wachman EM, Hayes MJ, Brown MS, et al. Association of OPRM1 and COMT Single-Nucleotide Polymorphisms With Hospital Length of Stay and Treatment of Neonatal Abstinence Syndrome. *Jama-Journal of the American Medical Association.* May 1 2013;309(17):1821-1827.

88. Wachman EM, Hayes MJ, Sherva R, et al. Variations in opioid receptor genes in neonatal abstinence syndrome. *Drug Alcohol Depend.* Oct 1 2015;155:253-259.

89. Cole FS, Wegner DJ, Davis JM. The Genomics of Neonatal Abstinence Syndrome. *Front Pediatr.* 2017;5:176.

90. Wittenberg E, Bray JW, Aden B, Gebremariam A, Nosyk B, Schackman BR. Measuring benefits of opioid misuse treatment for economic evaluation: health-related quality of life of opioid-dependent individuals and their spouses as assessed by a sample of the US population. *Addiction.* 2015.

91. Birnbaum HG, White AG, Schiller M, Waldman T, Cleveland JM, Roland CL. Societal costs of prescription opioid abuse, dependence, and misuse in the United States. *Pain medicine.* Apr 2011;12(4):657-667.

92. Mark TL, Woody GE, Juday T, Kleber HD. The economic costs of heroin addiction in the United States. *Drug and Alcohol Dependence.* Jan 1 2001;61(2):195-206.

93. The Council of Economic Advisers. *The Underestimated Cost of the Opioid Crisis*: The White House Office of the Press Secretary; 2017.

94. Murphy SM, Polsky D. Economic evaluations of opioid use disorder interventions: a systematic review. *PharmacoEconomics.* 2016;34(9):863-867.

95. Truven Health Analytics. Red Book. http://truvenhealth.com/products/micromedex/product-suites/clinical-knowledge/red-book. Accessed 06/28/2018.

96. Konijnenberg C, Melinder A. Prenatal exposure to methadone and buprenorphine: A review of the potential effects on cognitive development. *Child Neuropsychology.* 2011/09/01 2011;17(5):495-519.

97. Baldacchino A, Arbuckle K, Petrie DJ, McCowan C. Neurobehavioral consequences of chronic intrauterine opioid exposure in infants and preschool children: a systematic review and meta-analysis. *BMC Psychiatry.* Apr 8 2014;14:104.

98. Jones HE, O'Grady KE, Kaltenbach K. Reconsidering retrospective review of neurodevelopmental outcomes in infants treated for neonatal abstinence syndrome. *Journal of Perinatology.* Sep 2018;38(9):1280-1281.

99. Kaltenbach K, O'Grady KE, Heil SH, et al. Prenatal exposure to methadone or buprenorphine: Early childhood developmental outcomes. *Drug Alcohol Depend.* Apr 1 2018;185:40-49.

100. Jones HE, Heil S, O'Grady KE. Comment on: infants of opioid-dependent mothers: neurodevelopment at six months. *Early Hum Dev.* Mar 2015;91(3):243.

101. Veldhuizen S, Clinton J, Rodriguez C, Wade TJ, Cairney J. Concurrent validity of the Ages And Stages Questionnaires and Bayley Developmental Scales in a general population sample. *Acad Pediatr.* Mar-Apr 2015;15(2):231-237.

102. Bayley N, Aylward G. *Bayley Scales of Infant and Toddler Development–Fourth Edition*. Bloomington, MN: NCS Pearson; 2019.

103. American Society of Addiction Medicine. An Introduction to The ASAM Criteria for Patients and Families. Rockville, MD: American Society of Addiction Medicine; 2015.

104. Lofwall M, Walsh S, Nunes E, et al. Efficacy of weekly and monthly subcutaneous buprenorphine depots vs. daily sublingual buprenorphine with naloxone for outpatient treatment of opioid use disorder: A Randomized Clinical Trial. *JAMA Internal Medicine.* In press.

105. CLIAWaived Inc. Instant Drug Test Cup/Card II package insert. https://www.cliawaived.com/web/items/pdf/CWII-OTC-Multi-Drug%20Screen%20Test-Rev%201~3932file1.pdf. Accessed November 8, 2018.

106. CLIAWaived Inc. Multi-Drug Screen Test package insert. https://www.cliawaived.com/web/items/pdf/Multi-Drug%20Screen%20Test-Forensic%20Use-Rev8~4478file1.pdf. Accessed November 8, 2018.

107. Redwood Toxicology Laboratory. Confirmation Cutoff / LOQ Levels by Procedure. https://www.redwoodtoxicology.com/services/etg_testing. Accessed November 8, 2018.

108. Redwood Toxicology Laboratory. Confirmation Cutoff Levels By Procedure. https://www.redwoodtoxicology.com/services/comprehensive_screen-confirm. Accessed November 8, 2018.

109. American College of Obstetricians and Gynecologists. Committee Opinion No. 711: Opioid Use and Opioid Use Disorder in Pregnancy. *Obstetrics & Gynecology.* 2017;130(2):e81-e94.

110. Weiss RD, Potter JS, Griffin ML, et al. Long-term outcomes from the National Drug Abuse Treatment Clinical Trials Network Prescription Opioid Addiction Treatment Study. *Drug Alcohol Depend.* May 1 2015;150:112-119.

111. Hser YI, Evans E, Huang D, et al. Long-term outcomes after randomization to buprenorphine/naloxone versus methadone in a multi-site trial. *Addiction.* Apr 2016;111(4):695-705.

112. McHugh RK, Fitzmaurice GM, Carroll KM, et al. Assessing craving and its relationship to subsequent prescription opioid use among treatment-seeking prescription opioid dependent patients. *Drug Alcohol Depend.* Dec 1 2014;145:121-126.

113. Kotelchuck M. The Adequacy of Prenatal Care Utilization Index: its US distribution and association with low birthweight. *Am J Public Health.* Sep 1994;84(9):1486-1489.

114. VanderWeele TJ, Lantos JD, Siddique J, Lauderdale DS. A comparison of four prenatal care indices in birth outcome models: comparable results for predicting small-for-gestational-age outcome but different results for preterm birth or infant mortality. *J Clin Epidemiol.* Apr 2009;62(4):438-445.

115. Gossop M. The development of a Short Opiate Withdrawal Scale (SOWS). *Addict Behav.* 1990;15(5):487-490.

116. Vernon MK, Reinders S, Mannix S, Gullo K, Gorodetzky CW, Clinch T. Psychometric evaluation of the 10-item Short Opiate Withdrawal Scale-Gossop (SOWS-Gossop) in patients undergoing opioid detoxification. *Addict Behav.* Sep 2016;60:109-116.

117. Zigmond AS, Snaith RP. The hospital anxiety and depression scale. *Acta Psychiatr Scand.* Jun 1983;67(6):361-370.

118. Bjelland I, Dahl AA, Haug TT, Neckelmann D. The validity of the Hospital Anxiety and Depression Scale. An updated literature review. *J Psychosom Res.* Feb 2002;52(2):69-77.

119. Ito S. Opioids in Breast Milk: Pharmacokinetic Principles and Clinical Implications. *J Clin Pharmacol.* Oct 2018;58 Suppl 10:S151-S163.

120. Reece-Stremtan S, Marinelli KA. ABM clinical protocol #21: guidelines for breastfeeding and substance use or substance use disorder, revised 2015. *Breastfeed Med.* Apr 2015;10(3):135-141.

121. Winklbaur B, Baewert A, Jagsch R, et al. Association between prenatal tobacco exposure and outcome of neonates born to opioid-maintained mothers. Implications for treatment. *Eur Addict Res.* 2009;15(3):150-156.

122. Jones HE, Heil SH, Tuten M, et al. Cigarette smoking in opioid-dependent pregnant women: Neonatal and maternal outcomes. *Drug and Alcohol Dependence.* Aug 1 2013;131(3):271-277.

123. Jansson LM, Dipietro JA, Elko A, Velez M. Infant autonomic functioning and neonatal abstinence syndrome. *Drug Alcohol Depend.* Jun 1 2010;109(1-3):198-204.

124. Wachman EM, Warden AH, Thomas Z, et al. Impact of psychiatric medication co-exposure on Neonatal Abstinence Syndrome severity. *Drug Alcohol Depend.* Nov 1 2018;192:45-50.

125. Hall ES, Isemann BT, Wexelblatt SL, et al. A Cohort Comparison of Buprenorphine versus Methadone Treatment for Neonatal Abstinence Syndrome. *The Journal of pediatrics.* Mar 2016;170:39-44 e31.

126. Kraft WK, Adeniyi-Jones SC, Chervoneva I, et al. Buprenorphine for the Treatment of the Neonatal Abstinence Syndrome. *N Engl J Med.* Jun 15 2017;376(24):2341-2348.

127. Jansson LM, Choo R, Velez ML, et al. Methadone maintenance and breastfeeding in the neonatal period. *Pediatrics.* Jan 2008;121(1):106-114.

128. Bagley SM, Wachman EM, Holland E, Brogly SB. Review of the assessment and management of neonatal abstinence syndrome. *Addict Sci Clin Pract.* Sep 9 2014;9(1):19.

129. Grossman MR, Lipshaw MJ, Osborn RR, Berkwitt AK. A Novel Approach to Assessing Infants With Neonatal Abstinence Syndrome. *Hosp Pediatr.* Jan 2018;8(1):1-6.

130. Wachman EM, Grossman M, Schiff DM, et al. Quality improvement initiative to improve inpatient outcomes for Neonatal Abstinence Syndrome. *Journal of perinatology : official journal of the California Perinatal Association.* May 8 2018.

131. Baewert A, Jagsch R, Winklbaur B, et al. Influence of site differences between urban and rural American and Central European opioid-dependent pregnant women and neonatal outcome characteristics. *Eur Addict Res.* 2012;18(3):130-139.

132. Neumann PJ, Sanders GD, Russell LB, Siegel JE, Ganiats TG. *Cost-Effectiveness in Health and Medicine*. 2nd ed. New York, NY: Oxford University Press; 2017.

133. Braithwaite RS, Meltzer DO, King Jr JT, Leslie D, Roberts MS. What does the value of modern medicine say about the $50,000 per quality-adjusted life-year decision rule? *Medical care.* 2008;46(4):349-356.

134. Neumann PJ, Cohen JT, Weinstein MC. Updating cost-effectiveness—the curious resilience of the $50,000-per-QALY threshold. *New England Journal of Medicine.* 2014;371(9):796-797.

135. Bray JW, Aden B, Eggman AA, et al. Quality of life as an outcome of opioid use disorder treatment: A systematic review. *Journal of substance abuse treatment.* 2017;76:88-93.

136. U.S. Department of Health and Human Services. *Healthy People 2020.* Washington D.C. 2010.

137. Bhandari A, Wagner T. Self-reported utilization of health care services: improving measurement and accuracy. *Medical Care Research and Review.* 2006;63(2):217-235.

138. Murphy SM, Polsky D, Lee JD, et al. Cost-effectiveness of extended release naltrexone to prevent relapse among criminal-justice-involved persons with a history of opioid use disorder. *Addiction.* 2017;112(8):1440-1450.

139. Murphy SM, McDonell MG, McPherson S, et al. An economic evaluation of a contingency-management intervention for stimulant use among community mental health patients with serious mental illness. *Drug and Alcohol Dependence.* 2015.

140. Murphy SM, Campbell AN, Ghitza UE, et al. Cost-effectiveness of an internet-delivered treatment for substance abuse: data from a multisite randomized controlled trial. *Drug and Alcohol Dependence.* 2016.

141. Murphy SM, McCollister KE, Leff JA, et al. Cost-effectiveness of extended-release naltrexone versus buprenorphine-naloxone to prevent opioid relapse among individuals initiating treatment in an inpatient detoxification setting. Under review.

142. Cella D, Riley W, Stone A, et al. The Patient-Reported Outcomes Measurement Information System (PROMIS) developed and tested its first wave of adult self-reported health outcome item banks: 2005-2008. *J Clin Epidemiol.* Nov 2010;63(11):1179-1194.

143. HealthMeasures. PROMIS® (Patient-Reported Outcomes Measurement Information System). http://www.healthmeasures.net/explore-measurement-systems/promis. Accessed August 15, 2019.

144. EuroQol. EQ-5D. https://euroqol.org.

145. Health Utilities Inc. Health-Related Quality-of-Life. http://www.healthutilities.com. Accessed August 15, 2019.

146. Optum. SF Health Surveys. https://www.optum.com/solutions/life-sciences/answer-research/patient-insights/sf-health-surveys.html. Accessed August 15, 2019.

147. Kaplan RM, Anderson JP, Wu AW, Mathews WC, Kozin F, Orenstein D. The Quality of Well-being Scale. Applications in AIDS, cystic fibrosis, and arthritis. *Med Care.* Mar 1989;27(3 Suppl):S27-43.

148. Hanmer J, Dewitt B, Yu L, et al. Cross-sectional validation of the PROMIS-Preference scoring system. *PLoS One.* 2018;13(7):e0201093.

149. Hanmer J, Cella D, Feeny D, et al. Selection of key health domains from PROMIS((R)) for a generic preference-based scoring system. *Qual Life Res.* Dec 2017;26(12):3377-3385.

150. Dewitt B, Feeny D, Fischhoff B, et al. Estimation of a Preference-Based Summary Score for the Patient-Reported Outcomes Measurement Information System: The PROMIS((R))-Preference (PROPr) Scoring System. *Med Decis Making.* Aug 2018;38(6):683-698.

151. Polsky D, Glick HA, Yang J, Subramaniam GA, Poole SA, Woody GE. Cost-effectiveness of extended buprenorphine–naloxone treatment for opioid-dependent youth: data from a randomized trial. *Addiction.* 2010;105(9):1616-1624.

152. Hamilton CM, Strader LC, Pratt JG, et al. The PhenX Toolkit: get the most from your measures. *Am J Epidemiol.* Aug 1 2011;174(3):253-260.

153. Hasin DS, Trautman KD, Miele GM, Samet S, Smith M, Endicott J. Psychiatric Research Interview for Substance and Mental Disorders (PRISM): reliability for substance abusers. *Am J Psychiatry.* Sep 1996;153(9):1195-1201.

154. Rosenberg RE, Ahmed AS, Ahmed S, et al. Determining gestational age in a low-resource setting: validity of last menstrual period. *J Health Popul Nutr.* Jun 2009;27(3):332-338.

155. Hien DA, Wells EA, Jiang H, et al. Multisite randomized trial of behavioral interventions for women with co-occurring PTSD and substance use disorders. *J Consult Clin Psychol.* Aug 2009;77(4):607-619.

156. Carlson EB, Smith SR, Palmieri PA, et al. Development and validation of a brief self-report measure of trauma exposure: the Trauma History Screen. *Psychol Assess.* Jun 2011;23(2):463-477.

157. Beidas RS, Stewart RE, Walsh L, et al. Free, brief, and validated: Standardized instruments for low-resource mental health settings. *Cogn Behav Pract.* Feb 1 2015;22(1):5-19.

158. Warshaw C, Lyon E, Bland PJ, Phillips H, Hooper M. *Mental Health and Substance Use Coercion Surveys*: National Center on Domestic Violence, Trauma & Mental Health and the National Domestic Violence Hotline; 2014.

159. Blackwell TL, McDermott AN. Test Review: Patient Health Questionnaire–9 (PHQ-9). *Rehabilitation Counseling Bulletin.* 2014;57(4):246-248.

160. Akerman SC, Brunette MF, Green AI, Goodman DJ, Blunt HB, Heil SH. Treating tobacco use disorder in pregnant women in medication-assisted treatment for an opioid use disorder: a systematic review. *J Subst Abuse Treat.* May 2015;52:40-47.

161. Heatherton TF, Kozlowski LT, Frecker RC, Fagerstrom KO. The Fagerstrom Test for Nicotine Dependence: a revision of the Fagerstrom Tolerance Questionnaire. *Br J Addict.* Sep 1991;86(9):1119-1127.

162. Sobell LC, Toneatto T, Sobell MB, Leo GI, Johnson L. Alcohol abusers' perceptions of the accuracy of their self-reports of drinking: implications for treatment. *Addict Behav.* Sep-Oct 1992;17(5):507-511.

163. Fals-Stewart W, O'Farrell TJ, Freitas TT, McFarlin SK, Rutigliano P. The timeline followback reports of psychoactive substance use by drug-abusing patients: psychometric properties. *J Consult Clin Psychol.* Feb 2000;68(1):134-144.

164. Hall SM, Havassy BE, Wasserman DA. Effects of commitment to abstinence, positive moods, stress, and coping on relapse to cocaine use. *J Consult Clin Psychol.* Aug 1991;59(4):526-532.

165. Ondersma SJ, Winhusen T, Erickson SJ, Stine SM, Wang Y. Motivation Enhancement Therapy with pregnant substance-abusing women: does baseline motivation moderate efficacy? *Drug Alcohol Depend.* Apr 1 2009;101(1-2):74-79.

166. Winhusen T, Theobald J, Lewis D, Wilder CM, Lyons MS. Development and initial testing of a tailored telephone intervention delivered by peers to prevent recurring opioid-overdoses (TTIP-PRO). *Health Educ Res.* Apr 2016;31(2):146-160.

167. Cacciola JS, Alterman AI, Lynch KG, Martin JM, Beauchamp ML, McLellan AT. Initial reliability and validity studies of the revised Treatment Services Review (TSR-6). *Drug Alcohol Depend.* Jan 1 2008;92(1-3):37-47.

168. Chung EK, Siegel BS, Garg A, et al. Screening for Social Determinants of Health Among Children and Families Living in Poverty: A Guide for Clinicians. *Curr Probl Pediatr Adolesc Health Care.* May 2016;46(5):135-153.

169. Shonkoff JP, Garner AS, Committee on Psychosocial Aspects of C, et al. The lifelong effects of early childhood adversity and toxic stress. *Pediatrics.* Jan 2012;129(1):e232-246.

170. Jellinek M, Patel B, Froehle M, eds. *Bright Futures in Practice: Mental Health—Volume II. Tool Kit*. Arlington, VA: National Center for Education in Maternal and Child Health; 2002.

171. Kemper KJ. SELF-ADMINISTERED QUESTIONNAIRE FOR STRUCTURED PSYCHOSOCIAL SCREENING IN PEDIATRICS. *Pediatrics.* Mar 1992;89(3):433-436.

172. Salisbury AL, Coyle MG, O'Grady KE, et al. Fetal assessment before and after dosing with buprenorphine or methadone. *Addiction.* Nov 2012;107 Suppl 1:36-44.

173. Abdullah B, Muadz B, Norizal MN, Ismail N, Kornain NK, Kutty M. Pregnancy outcome and cord blood cotinine level: A cross-sectional comparative study between secondhand smokers and non-secondhand smokers. *European journal of obstetrics, gynecology, and reproductive biology.* Jul 2017;214:86-90.

174. Achenbach T, Rescorla L. *Manual for the ASEBA Preschool Forms & Profiles*. Burlington, VT: University of Vermont, Research Center for Children, Youth, & Families; 2000.

175. Frost M, Bailey G, Budilovsky-Kelley N, Kim S. Transitioning patients from sublingual to injectable weekly and monthly buprenorphine. *American Society of Addiction Medicine 49th Annual Conference*. San Diego, CA 2018.

176. FDA Center for Drug Evaluation and Research and Center for Biologics Evaluation and Research. *Non-Inferiority Clinical Trials to Establish Effectiveness: Guidance for Industry.* Silver Spring, MD: U.S. Department of Health and Human Services; 2016.

177. Socias ME, Ahamad K, Le Foll B, et al. The OPTIMA study, buprenorphine/naloxone and methadone models of care for the treatment of prescription opioid use disorder: Study design and rationale. *Contemp Clin Trials.* Jun 2018;69:21-27.

178. Bryan MA, Smid MC, Cheng M, et al. Addressing opioid use disorder among rural pregnant and postpartum women: a study protocol. *Addict Sci Clin Pract.* Oct 31 2020;15(1):33.

179. Andrilla CHA, Coulthard C, Larson EH. Barriers Rural Physicians Face Prescribing Buprenorphine for Opioid Use Disorder. *Ann Fam Med.* Jul 2017;15(4):359-362.

180. Andrilla CHA, Jones KC, Patterson DG. Prescribing Practices of Nurse Practitioners and Physician Assistants Waivered to Prescribe Buprenorphine and the Barriers They Experience Prescribing Buprenorphine. *J Rural Health.* Mar 2020;36(2):187-195.

181. Committee for Proprietary Medicinal Products. Points to Consider on Switching Between Superiority and Non-inferiority. London: European Agency for the Evaluation of Medicinal Products; 2000.

182. Fischer G, Ortner R, Rohrmeister K, et al. Methadone versus buprenorphine in pregnant addicts: a double-blind, double-dummy comparison study. *Addiction.* Feb 2006;101(2):275-281.

183. Lan KKG, Demets DL. Discrete sequential boundaries for clinical trials. *Biometrika.* 1983;70(3):659-663.

184. O'Brien PC, Fleming TR. A multiple testing procedure for clinical trials. *Biometrics.* Sep 1979;35(3):549-556.

185. Jennison C, Turnbull BW. *Group sequential methods with applications to clinical trials*. New York: Chapman & Hall/CRC; 2000.

186. Kline RB. *Principles and practice of structural equation modeling, 4th ed*. New York, NY, US: Guilford Press; 2016.

187. Drummond MF, Schulpher MJ, Claxton K, Stoddart GL, Torrance GW. *Methods for the Economic Evaluation of Health Care Programmes*. Fourth ed: Oxford university press; 2015.

188. Glick HA, Doshi JA, Sonnad SS, Polsky D. *Economic Evaluation in Clinical Trials*: Oxford University Press; 2014.

189. Drummond MF, Davies L. Economic analysis alongside clinical trials. *Int J Technol Assess Health Care.* 1991;7(4):561-573.

190. Brady T, Robinson B. *Medicare Hospital Prospective Payment System: How DRG Rates Are Calculated and Updated*: Health and Human Services Office of Inspector General; 2001.

191. Matthews JNS, Altman DG, Campbell MJ, Royston P. Analysis of serial measurements in medical research. *British Medical Journal.* Jan 27 1990;300(6719):230-235.

192. Little RJA. Missing data. In: Everitt BS, Howell DC, eds. *Encyclopedia of Statistics in Behavioral Science*. Chichester, England: Wiley; 2005:1234-1238.

193. Enders CK. *Applied Missing Data Analysis*: Guilford Press; 2010.

16.0 APPENDIX A: ADVERSE EVENT REPORTING AND PROCEDURES

Each participating site's Principal Investigator is responsible for study oversight, including ensuring human research subject protection by designating appropriately qualified and trained study personnel to assess, report, and monitor adverse events.

Definition of Adverse Events and Serious Adverse Events

An **adverse event** (AE) is any untoward medical occurrence in humans, whether or not considered study medication related which occurs during the conduct of a clinical trial. Any change from baseline in clinical status, ECGs, lab results, x-rays, physical examinations, etc., that is considered clinically significant by the study medical clinician are considered AEs.

Suspected adverse reaction is any adverse event for which there is a reasonable possibility that the study medication caused the adverse event. A reasonable possibility implies that there is evidence that the study medication caused the event.

Adverse reaction is any adverse event caused by the study medication.

An **adverse event, suspected adverse reaction,** or **adverse reaction** is considered "**serious**" (i.e., a serious adverse event, serious suspected adverse reaction or serious adverse reaction) if, in the view of either the study medical clinician or sponsor, it:

1. Results in death: A death occurring during the study or which comes to the attention of the study staff during the protocol-defined follow-up period, whether or not considered caused by the study medication, must be reported.
2. Is life-threatening: Life-threatening means that the study participant was, in the opinion of the medical clinician or sponsor, at immediate risk of death from the reaction as it occurred and required immediate intervention.
3. Requires inpatient hospitalization or prolongation of existing hospitalization.
4. Results in persistent or significant incapacity or substantial disruption of the ability to conduct normal life functions.
5. Is a congenital abnormality or birth defect.
6. Is an important medical event that may not result in one of the above outcomes, but may jeopardize the health of the study participant or require medical or surgical intervention to prevent one of the outcomes listed in the above definition of serious event.

Definition of Expectedness

Any adverse event is considered "unexpected" if it is not listed in the investigator's brochure or the package insert or is not listed at the specificity or severity that has been observed. If neither is available, then the protocol and consent are used to determine an unexpected adverse event.

Medical and Psychiatric History

A thorough medical and psychiatric history during the baseline phase should record any chronic, acute, or intermittent preexisting or current illnesses, diseases, symptoms, or laboratory signs of the participant, to avoid reporting pre-existing conditions as new AEs and to assist in the assessment of worsening in intensity or severity of these conditions that would indicate an AE. Stable chronic conditions, such as arthritis, which are present prior to clinical trial entry and do not worsen are not considered AEs.

Site's Role in Eliciting and Reporting Adverse Events

Appropriately qualified and trained personnel will elicit participant reporting of AEs and SAEs at each study visit designated to collect AEs. Adverse events (medical and/or psychiatric) assessment will initiate with participant consent and follow-up of reported AEs will continue through 30 days post last study visit. Study personnel will obtain as much information as possible about the reported AE/SAE to complete the AE/SAE forms and will consult as warranted.

Standard reporting, within 7 days of the site becoming aware of the event, is required for reportable AEs. Expedited reporting (within 24 hours of their occurrence and/or site's knowledge of the event) is required for reportable SAEs (including death and life-threatening events). Local sites are responsible for reporting SAEs to the IRB of record, per the IRB of record's guidelines.

Sites are required to enter reportable AEs and SAEs in the Advantage eClinical system. The AE form is used to capture reportable AEs and SAEs (as defined in the protocol). Additional information may need to be gathered to evaluate SAEs and to complete the appropriate CRFs and the summary. This process may include obtaining hospital discharge reports, medical records, autopsy records or any other type records or information necessary to provide a complete and clear picture of the serious event and events preceding and following the event. If the SAE is not resolved or stable at the time of the initial report or if new information becomes available after the initial report, follow-up information must be submitted as soon as possible.

Reportable adverse events will be followed until resolution, stabilization or study end. Any serious adverse reactions will be followed until resolution or stabilization even beyond the end of the study.

Site's Role in Assessing Severity and Causality of Adverse Events

Appropriately qualified and trained study personnel will conduct an initial assessment of seriousness, severity, and causality when eliciting participant reporting of adverse events. A study medical clinician will review reportable AEs for seriousness, severity, and causality on at least a weekly basis.

Guidelines for Assessing Severity

The severity of an adverse event refers to the intensity of the event:

Grade 1	Mild	Transient or mild discomfort (typically <48 hours), no or minimal medical intervention/therapy required, hospitalization not necessary (non-prescription or single-use prescription therapy may be employed to relieve symptoms, e.g., aspirin for simple headache, acetaminophen for post-surgical pain)
Grade 2	Moderate	Mild to moderate limitation in activity, some assistance may be needed; no or minimal intervention/therapy required, hospitalization possible.
Grade 3	Severe	Marked limitation in activity, some assistance usually required; medical intervention/ therapy required, hospitalization possible.

Guidelines for Determining Causality

The study medical clinician will use the following question when assessing causality of an adverse event to study medication where an affirmative answer designates the event as a suspected adverse reaction:

Is there a reasonable possibility that the study medication caused the event?

Site's Role in Monitoring Adverse Events

Local quality assurance monitors will review study sites and respective study data on a regular basis and will promptly advise sites to report any previously unreported safety issues and ensure that the reportable safety-related events are being followed to resolution and reported appropriately. Staff education, re-training or appropriate corrective action plan will be implemented at the participating site when unreported or unidentified reportable AEs or serious events are discovered, to ensure future identification and timely reporting by the site.

Sponsor's Role in Safety Management Procedures of AEs/SAEs

A NIDA-assigned Medical Monitor is responsible for reviewing all serious adverse event reports. All reported SAEs will generate an e-mail notification to the Medical Monitor, Lead Investigator, and designees. All SAEs will be reviewed by the Medical Monitor in Advantage eClinical and, if needed, additional information will be requested. The Medical Monitor will also report events to the sponsor, pharmaceutical company (Braeburn), and the DSMB. The DSMB will receive summary reports of all adverse events annually, at a minimum. The DSMB or the NIDA assigned Medical Monitor may also request additional and updated information. Details regarding specific adverse events, their treatment and resolution, will be summarized by the Medical Monitor in writing for review by the sponsor and DSMB. Subsequent review by the Medical Monitor, DSMB, FDA and ethics review committee or IRB, the sponsor, or relevant local regulatory authorities may also suspend further trial treatment at a site. The study sponsor,

DSMB and FDA retain the authority to suspend additional enrollment and treatments for the entire study as applicable.

Reporting to the Data and Safety Monitoring Board

The DSMB will receive listings of AEs and summary reports of all SAEs at a frequency requested by the DSMB, but at least annually. Furthermore, the DSMB will be informed of expedited reports of SAEs.

Regulatory Reporting for an IND study

All serious and unexpected suspected adverse reactions are reported by the medical monitor on behalf of the sponsor to the FDA in writing within 15 calendar days of notification. Suspected adverse reactions that are unexpected and meet the criteria for death or immediately life-threatening also require notification of the FDA as soon as possible but no later than 7 calendar days of notification of the event, with a follow-up written report within 15 calendar days of notification of the event. The medical monitor will prepare an expedited report (MedWatch Form 3500A or similar) for the FDA and other regulatory authorities, DSMB and copies will be distributed to all sites. Expedited reports will be placed in the site regulatory files upon receipt. A copy of all expedited reports will be forwarded to the site's local IRB, as required.

Participant Withdrawal

The study medical clinician must apply his/her clinical judgment to determine whether or not an adverse event is of sufficient severity to require that the participant be withdrawn from further study medication administration. The study medical clinician should consult with the site Principal Investigator, the Lead Investigator and/or Medical Monitor as needed. If necessary, a study medical clinician may suspend any trial treatments and institute the necessary medical therapy to protect a participant from any immediate danger. A participant may also voluntarily withdraw from treatment due to what he/she perceives as an intolerable adverse event or for any other reason. If voluntary withdrawal is requested, the participant will be asked to complete an end-of-medication visit to assure safety and to document end-of-medication outcomes and will be given recommendations for medical care and/or referrals to treatment, as necessary.

Adverse Event Reporting (Chart)

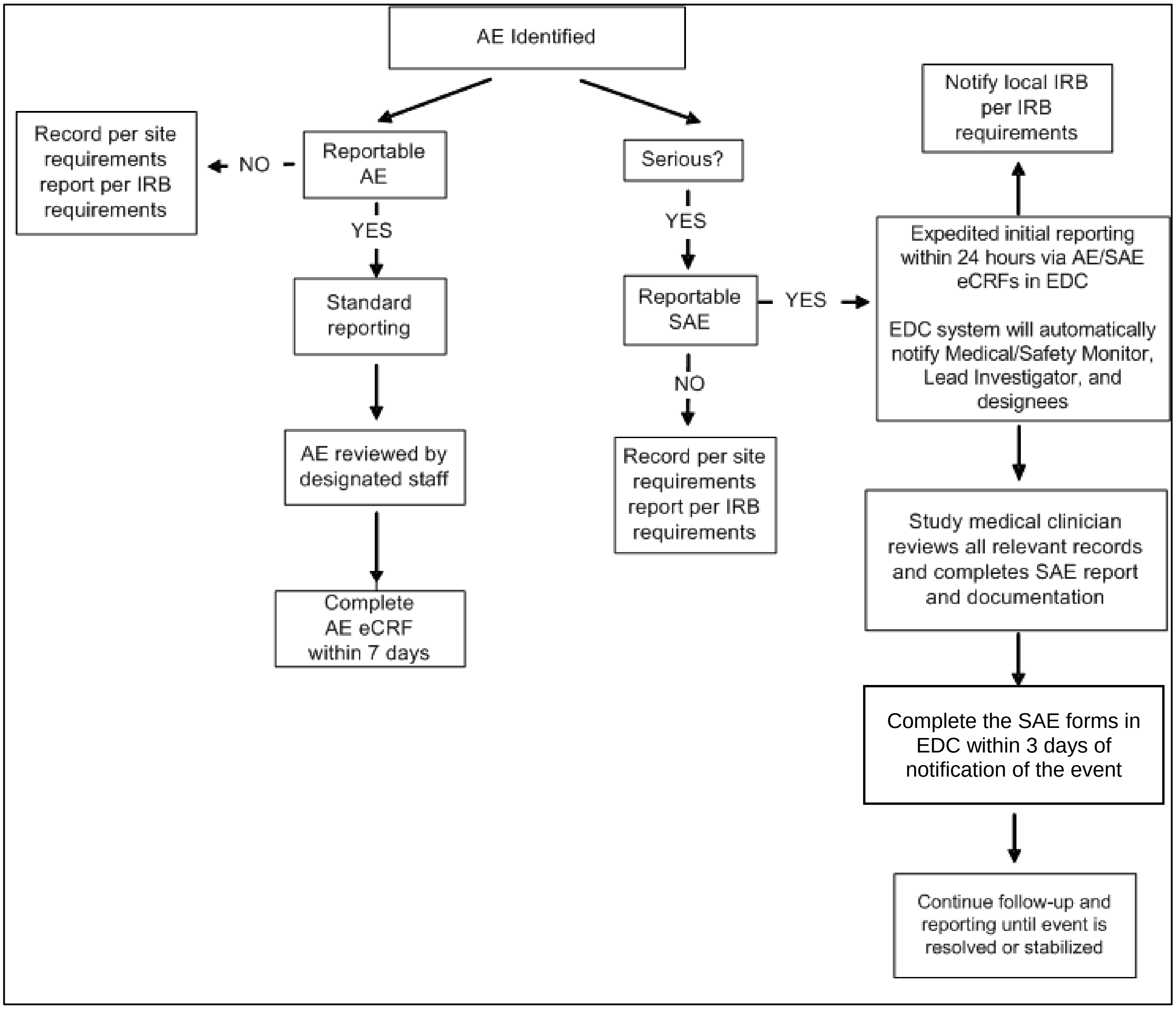

17.0 APPENDIX B: DATA AND SAFETY MONITORING PLAN

17.1 Brief Study Overview

17.1.1 Protocol Description

The primary objective of this trial is to evaluate the impact of treating opioid use disorder (OUD) in pregnant women with extended-release (XR) buprenorphine (BUP), compared to sublingual (SL) BUP, on mother and infant outcomes. It is hypothesized that the BUP-XR, relative to the BUP-SL, group will: 1) not have greater illicit opioid use during pregnancy (primary, non-inferiority); 2) have lower infant neonatal opioid withdrawal syndrome (NOWS) severity (key secondary, superiority); and 3) not have greater postpartum illicit opioid use (key secondary, non-inferiority). Testing conceptual models of the mechanisms by which BUP-XR may improve mother-infant outcomes, relative to BUP-SL, is a secondary objective. Determining the economic value of BUP-XR, compared to BUP-SL, to treat pregnant women is a tertiary objective. A final objective is evaluating the impact of BUP-XR, relative to BUP-SL, on infant neurodevelopment.

17.1.2 Key outcome measures

Mother key outcomes include illicit opioid abstinence during pregnancy and postpartum, assessed by urine drug screens (UDSs). Total days of infant opioid treatment, derived from the infant's medical record, is the infant key outcome. The Conceptual Model Assessment (CMA) sub-study includes assessments of: 1) maternal trough BUP plasma concentrations at study weeks 3 and 5; 2) fetal non-stress test and biophysical profile at ~36 weeks EGA at maternal peak BUP plasma level; 3) maternal peak and trough BUP plasma concentrations at ~36 weeks EGA; and 4) cord plasma BUP/BUP-metabolite levels. The main economic outcome will be the incremental cost-effectiveness ratio (ICER). The main outcome for the INO sub-study is the score on the cognitive subscale of the Bayley™-4 at 24 months of age.

17.1.3 Inclusion/Exclusion Criteria:

All potential participants will be recruited from participating sites. Eligible participants will meet all inclusion, and no exclusion criteria:

Inclusion Criteria:

Potential participants must:

1. be 18-41 years of age;
2. be pregnant with an EGA of 6 - 30 weeks at randomization, have evidence of a viable intrauterine pregnancy if EGA <12 weeks, and are not planning to terminate the pregnancy;
3. have a single fetus pregnancy (can be based on self-report if an objective assessment is unavailable);
4. meet DSM-5 criteria for moderate/severe OUD and be a good candidate for BUP maintenance and/or be currently prescribed BUP for the treatment of OUD;
5. be willing to be randomized to BUP-XR or BUP-SL and to comply with study procedures, including weekly Medication Check Visits;

6. be planning to deliver at one of the hospitals for which the BORN survey was completed and that: a) has a written protocol for the management of NAS/NOWS, b) offers rooming-in while infants are being observed for NAS/NOWS; and c) does not send infants home on opioids for the treatment of NAS/NOWS;
7. be enrolled in outpatient addiction treatment at a participating site (e.g., have completed intake);
8. be able to understand the study, and having understood, provide written informed consent in English.

Exclusion Criteria:

Potential participants must not:

1. have a physiological dependence on alcohol or sedatives requiring medical detoxification;
2. have a psychiatric condition that, in the judgment of the site medical clinician (MC), would make study participation unsafe or which would make treatment compliance difficult;

   Examples include:

   - Suicidal or homicidal ideation requiring immediate attention
   - Severe, inadequately-treated mental health disorder (e.g., active psychosis, uncontrolled bipolar disorder)
3. have a medical condition that, in the judgment of the site MC, would make study participation unsafe or which would make treatment compliance difficult. Medical conditions that may compromise participant safety or study conduct include, but are not limited to, allergy/sensitivity to study medications and the following based on clinical labs:
   - AST/ALT greater than 5X upper limit of normal
   - serum creatinine greater than 1.5X upper limit of normal
   - total bilirubin greater than 1.5X upper limit of normal
4. be currently in jail, prison or any inpatient overnight facility as required by court of law or have pending legal action or other situation (e.g., unstable living arrangements) that, in the judgement of the site investigator, could prevent participation in the study or in any study activities;
5. be currently receiving methadone or naltrexone for treatment;
6. be enrolled in or planning to enroll in treatment beyond the level of 3.1 (clinically managed low-intensity residential services) of the American Society of Addiction Medicine criteria;[103]
7. be enrolled in or planning to enroll in: a) a trial testing medication for managing OUD during pregnancy; b) research testing an intervention for substance use disorder or NOWS in their infant unless they are willing to provide a release for the research records.

17.1.4 Sample Size

Approximately 200 participants, recruited from approximately 10 sites, will be randomized into the trial.

17.1.5 Overview of Protocol Monitoring

Four separate entities will be involved in the monitoring of this study — the participating CTN Nodes, the Lead Node, the Data and Statistics Center (DSC - Emmes), and the Clinical Coordinating Center (CCC - Emmes). All aspects of the study will be carefully monitored with respect to current good clinical practices. A study-specific QA plan will be developed to include standard reporting templates for use by local monitoring teams. The following includes a summary of the responsibilities for each team.

The CTN nodes will be responsible for completing monitoring activities in accordance with the study QA plan during the pre-initiation, recruitment, enrollment, follow up, and close-out phases. These activities will be conducted by local QA monitors located at each Node and aim to provide management support to the site research team in order to ensure adherence to the protocol, SOPs, and regulatory requirements and accurate data entry. Qualified node personnel will provide site management for each site during the trial. This will take place as often as needed to help prevent, detect, and correct problems at the study sites.

The Lead Node will provide on-going monitoring of study progress through review of weekly enrollment reports, as well as by reviewing monitoring reports from both local and CCC/Emmes monitors and regular data reports from the DSC/Emmes. The Lead Node will hold regular study management meetings to monitor any emergent problems or ongoing problematic trends, and may additionally hold individual meetings with site staff in order to assist in resolving any site-specific problems that impact the study. The Data and Statistics Center (DSC/Emmes) will conduct on-going monitoring of data completeness, accuracy, and quality through the use of reports within Advantage eClinical and regularly generated trial progress and data status reports. The Clinical Coordinating Center (CCC/Emmes) will focus on providing study monitoring to specifically address areas of regulatory compliance, safety compliance, source document verification and specific study related monitoring addressed below in the project detail. The four monitoring entities will aim to communicate and work together in scheduling visits (in order to avoid overlapping visits) and to cover a broad sample of study participants by auditing different participants to the extent possible.

17.2 Oversight of Clinical Responsibilities

17.2.1 Site Principal Investigator (PI)

Each participating site's PI is responsible for study oversight, including ensuring maintenance of secure study medication supply and human research subject protection by designating appropriately qualified, trained research staff and medical clinicians to assess, report, and monitor adverse events.

All adverse events (AEs) occurring during the course of the clinical trial will be collected, documented, and reported by the investigator or sub-investigators according to the Protocol.

The occurrence of AEs and serious adverse events (SAEs) will be assessed at each clinic visit during the study. SAEs will be followed until resolved or considered stable.

Reportable AEs are required to be entered into the data system within 7 days of the site staff becoming aware of the event. Reportable SAEs (including death and life-threatening events) are required to be entered into data system within 24 hours of site's knowledge of the event).

17.2.2 CCC Medical Monitor

The NIDA CTN Clinical Coordinating Center’s (CCC) Medical Monitor or designee is responsible for reviewing all adverse events and serious adverse events reported. The CCC Medical Monitor is alerted via email each time an SAE is reported in the EDC. All SAEs will be reviewed at the time they are reported in the EDC. The Medical Monitor or designee will also indicate concurrence or not with the details of the report provided by the site PI. Where further information is needed, the Medical Monitor or designee will discuss the event with the site staff. Reviews of SAEs by the CCC Medical Monitor or designee will be documented in the Advantage eClinical data system and will be a part of the safety database. All AEs are reviewed on a weekly basis to observe trends or unusual events.

17.2.3 Mandatory Regulatory Reporting in IND Trials

For trials conducted under IND, the CCC Medical Monitor or designee will report events to the regulatory authorities if the event meets the definition of an expedited event (21CFR312.32). All SAEs that meet expedited reporting will be reported to the FDA/Regulatory Authorities in writing within 15 calendar days of notification of the CCC. If the SAE also meets the criteria for death or immediately life-threatening, the CCC will notify the FDA/Regulatory Authorities electronically, by phone or by fax as soon as possible but no later than 7 calendar days of notification of the CCC pharmacovigilance team, with a follow-up written report within 15 calendar days of notification of the CCC. The CCC pharmacovigilance team will prepare an expedited report (MedWatch Form 3500A or similar) for the FDA/Regulatory authorities and copies will be distributed to all participating site investigators.

17.3 Data and Safety Monitoring Board (DSMB)

The NIDA CTN DSMB affiliated with this trial will be responsible for conducting periodic reviews of accumulating safety, trial performance, and outcome data. Reports will be generated and presented for Data and Safety Monitoring Board (DSMB) meetings. The DSMB will receive listings of reportable AEs and summary reports of all SAEs at a frequency requested by the DSMB, but at least annually. Furthermore, the DSMB will be informed of expedited reports of SAEs. The DSMB will make recommendations to NIDA CCTN as to whether there is sufficient support for continuation of the trial, evidence that study procedures should be changed, or evidence that the trial (or a specific site) should be halted for reasons relating to safety of the study participants or inadequate trial performance (e.g., poor recruitment).

Following each DSMB meeting, the NIDA CCTN will communicate the outcomes of the meeting, based on DSMB recommendations, in writing to the study Lead Investigator. This communication summarizing study safety information will be submitted to participating sites and the study sIRB.

17.4 Quality Assurance (QA) Monitoring

The Lead Team has developed a study-specific QA Plan for the guidance of QA activities in this study. The monitoring of the study site(s) will be conducted on a regular basis using a combination of NIDA CCTN CCC monitors and the local Node QA Monitors. Investigators will host periodic visits for the NIDA CCTN CCC monitors and local Node QA Monitors. The purpose of these visits is to assess compliance with the protocol, GCP requirements, and other

applicable regulatory requirements, as well as to document the integrity of the trial progress. The investigative site will provide direct access to all trial related sites (e.g., pharmacy, research office), source data/documentation, and reports for the purpose of monitoring and auditing by the CCC and local Node monitors, as well as inspection by local and regulatory authorities. Areas of particular concern will be the review of inclusion/exclusion criteria, participant Informed Consent Forms, protocol adherence, safety monitoring, IRB reviews and approvals, regulatory documents, participant records, study drug accountability, and Principal Investigator supervision and involvement in the trial. The monitors will interact with the site staff to identify issues and re-train the site as needed to enhance research quality.

Both the Local Node monitors and the NIDA CCC monitors will complete written QA visit reports following their respective site visits. These reports will be sent to the site Principal Investigator, the study Lead Node, the CCC Protocol Specialist, and NIDA CCTN.

17.5 Management of Risks to Participants

17.5.1 Confidentiality

Confidentiality of participant records will be secured by the use of study codes for identifying participants on CRFs, secure transportation of documents while performing study visits at any off-site location, and secure storage of any documents that have participant identifiers on site, as well as secure computing procedures for entering and transferring electronic data. The documents or logs linking the study codes with the study participant on site will be kept locked separately from the study files and the medical records. No identifying information will be disclosed in reports, publications or presentations.

17.5.2 Information That Meets Reporting Requirements

The consent forms will specifically state the types of information that are required for reporting and that the information will be reported as required. These include suspected or known sexual or physical abuse of a child or elders, or threatened violence to self and/or others.

17.5.3 Participant Protection

The site’s Physician/Medical Clinician will evaluate all pertinent screening and baseline assessments prior to participant randomization to ensure that the participant is eligible and safe to enter the study. AEs and concomitant medications will be assessed and documented at each study visit. Individuals who experience an AE that compromises safe participation in the study, or who voluntarily wish to be discontinued from the study medication, will be safely and humanely discontinued from further study medication administration and provided referrals for other treatment or to specialized care. Study personnel will complete early termination of the study medication on a Medication Discontinuation form. In addition, study personnel will offer the participant an opportunity to continue study visits without medication; if the participant declines to continue study participation, study personnel will attempt to complete a final study visit to assure safety and to document end of treatment outcomes.

17.6 Pregnancy and Pregnancy Outcomes

Pregnancy outcomes will be captured as part of regular study data collection using information abstracted from the medical records for the participant and her neonate (if any). Pregnancy initiated in the 12-month post-partum period will not be considered an AE or SAE, but will be captured on a Pregnancy form.

17.7 Study Specific Risks

Both BUP-SL and BUP-XR are comprised of buprenorphine, which is an opioid medication. As such, withdrawal reactions may occur if the medication is discontinued abruptly. Prolonged use of opioids during pregnancy for medical or non-medical reasons can result in neonatal opioid withdrawal syndrome which may be life-threatening if not recognized and treated; however, research supports lower NAS/NOWS severity in the infants of mothers with opioid use disorder who are medically treated with BUP. Participants in this study will be provided with a medication information card that will notify clinicians that they are receiving BUP as part of this study.

17.8 Collection and management of AEs and SAEs

All adverse events (AEs) occurring during the course of the clinical trial will be collected, documented, and reported by the investigator or sub-investigators according to the Protocol.

The occurrence of AEs and serious adverse events (SAEs) will be assessed at each study visit following the initial screening visit. Serious adverse events will be followed until resolved or considered stable.

AEs are required to be entered into the data system within 7 days of the site staff becoming aware of the event. Reportable SAEs (including death and life-threatening events) are required to be entered into data system within 24 hours of site's knowledge of the event).

Serious Adverse Events (SAEs) will also be initially captured on the Adverse Events CRF. An SAE is defined as any event that occurs during the 'active' phase of treatment, or the follow-up periods, and either: (1) results in death, or (2) requires inpatient hospitalization (EXCEPT for the instances specified below) or a prolongation of existing hospitalization, or (3) is a congenital anomaly/birth defect, or (4) results in persistent or significant disability / incapacity, or (5) is life-threatening (EXCEPT for an opioid overdose not resulting in hospitalization or death), or (6) requires intervention to prevent one of the above outcomes.

For the purposes of this study, several exceptions to an episode of inpatient hospitalization will be specified and will not be reported as a Serious Adverse Event. These Exceptions are:

(A) hospitalization for labor and delivery unless the hospitalization results in an ICU admission.

(B) prolongation of the hospital stay for a delivery (unless greater than 4 days for vaginal delivery or 7 days for C-section delivery)

(C) admission to a hospital for elective surgery or pre-scheduled diagnostic tests.

After capture on the AE CRF, the SAE form will be initiated by the research assistant (RA), and the following individuals will be notified within 24 hours of the site's initial receipt of the information: (1) the Investigator at the site (e.g., the Executive or Clinical Director, etc.), who will direct the appropriate review, then complete and sign the follow-up SAE Form, (2) the Node/Lead Node Regulatory staff person, who will notify the IRB according to their procedures, (3) the Project Coordinator, (4) the Site Physician/Medical Clinician, who will review the event for relatedness to the study, and (5) the NIDA Medical Safety Officer, who will independently review each SAE for relatedness and expectedness.

Following the initial 24-hour SAE report, additional information will be gathered to enable an assessment of the event for relatedness. For example, psychiatric history, baseline severity of illness, treatment compliance, and verbal or objective information about drug use at the time of

the event are pertinent. The site Investigator will attach copies of source documents to the follow-up SAE form, which will be forwarded to the NIDA Medical Safety Officer within 2 weeks of the initial SAE report. The NIDA Medical Safety Officer will accumulate individual SAE reports from all sites involved in the study and summarize them in a table of SAEs. The cumulative SAEs will be sent to the DSMB each quarter, to review for possible study-related toxicities. Recommendations from the DSMB will be communicated via NIDA in a summary letter to the LI; it is the LI's responsibility to forward this letter to the Node PIs, who in turn will convey it to their appropriate IRB(s).

17.9 Data Oversight

This protocol will utilize a centralized Data and Statistics Center (DSC). A web-based distributed data entry model will be implemented. This electronic data capture system (Advantage eClinical) will be developed to ensure that guidelines and regulations surrounding the use of computerized systems in clinical trials are upheld.

17.9.1 Data and Statistics Center Responsibility

The DSC will: 1) develop and apply data management procedures to ensure the collection of accurate and good-quality data, 2) provide source documents and electronic Case Report Forms (eCRFs) for the collection of all participant data required by the study, 3) develop data dictionaries for each eCRF that will comprehensively define each data element, 4) prepare instructions for the use of Advantage eClinical and for the completion of eCRFs, 5) conduct ongoing monitoring activities on study data collected from all participating sites, and 6) perform data cleaning activities prior to the final study database lock.

17.9.2 Data Collection and Entry

Data will be collected at the study visits on source documents and entered by the site into eCRFs in Advantage eClinical or will be collected via direct entry into the eCRF or will be uploaded from a central lab into Advantage eClinical. In the event that Advantage eClinical is not available, the DSC will provide the sites with paper source documents and completion instructions. Data will be entered into Advantage eClinical in accordance with the instructions provided during protocol-specific training and guidelines established by the DSC. Data entry into the eCRFs is performed by authorized individuals. Selected eCRFs may also require the investigator's electronic signature. In some situations, data collected on source documents will not be entered into Advantage eClinical, but when it is entered, it will follow the guidelines stated above.

The investigator at the site is responsible for maintaining accurate, complete and up-to-date research records. In addition, the investigator is responsible for ensuring the timely completion of eCRFs for each research participant.

17.9.3 Data Monitoring, Cleaning, and Editing

eCRFs will be monitored for completeness and accuracy throughout the study. Dynamic reports listing missing values and forms are available to sites at all times in Advantage eClinical. These reports will be monitored regularly by the DSC. In addition, the DSC will identify inconsistencies within eCRFs and between eCRFs and post queries in Advantage eClinical on a scheduled basis. Sites will resolve data queries by entering all corrections and changes directly into Advantage eClinical or verifying the data are correct as is.

As described above, the CCC will conduct regular site visits, during which audits comparing source documents to the data entered on the eCRF will be performed. Any discrepancies identified between the source document and the eCRF will be corrected by the site.

Trial progress and data status reports, which provide information on recruitment, availability of primary outcome, treatment exposure, attendance at long term follow-up visits, regulatory status, and data quality, will be generated daily and posted to a secure website. These reports are available to the site, the corresponding Node, the lead investigator, the coordinating centers, and NIDA CCTN, to monitor the sites' progress on the study.

17.9.4 Database Lock and Transfer

At the conclusion of data collection for the study, the DSC will perform final data cleaning activities and will "lock" the study database from further modification. The final analysis dataset will be transferred to the Lead Investigator or designee. De-identified versions of these datasets will also be provided to the NIDA CCTN-designated parties for posting on Datashare, as well as storage and archiving. Results of the study will also be entered into www.clinicaltrials.gov by the Lead Investigative Team.

Reference: http://grants.nih.gov/grants/guide/notice-files/not98-084.html

18.0 APPENDIX C: REGULATORY

18.1 Single IRB

All study sites and corresponding IRBs not already part of the University of Cincinnati must agree to adhere to all policies outlined by The National Drug Abuse Treatment Clinical Trials Network (UG1) as indicated in RFA DA 15 008. UC has already agreed to adhere to this policy. Prior to confirmation of site selection, each site's IRB must provide, in writing, an agreement that they will be willing to rely on the UC IRB as the single IRB of record for this study, pending whatever local context reviews they perform.

18.2 Study Discontinuation

Individual study participants will be informed of their right to discontinue study participation at any time during the study. The PI may discontinue a participant from the trial if deemed clinically appropriate. The DSMB may recommend study termination based on review of site performance or safety and efficacy data. NIDA has the right to discontinue the investigation at any time.

Statistical Analysis Plan for
The Primary Outcome Paper for
NIDA Protocol CTN-0080

Medication treatment for Opioid use disorder in expectant Mothers (MOMs): a pragmatic randomized trial comparing extended-release and daily buprenorphine formulations (MOMS trial)

Lead Investigator: T. John Winhusen, PhD

Sub-Investigator: Michelle Lofwall MD

July 18, 2025

Prepared by: NIDA CTN Data and Statistics Center

And Daniel Lewis, University of Cincinnati

Contents

1.0 Introduction 1
1.1 Table of Abbreviations 1
1.2 Scope of This Document 2
1.3 Changes from the Original SAP 2
1.4 Overview of Statistical Analyses 2
1.4.1 General Overview 2
1.4.2 Significance Testing and Multiplicity 2
1.4.3 The Pregnancy and Postpartum Study Phases 3
1.4.4 Overview of Regression Analyses 3
1.4.5 Table shells 3
1.4.6 Medication Characterization 3
2.0 Summary of Baseline Characteristics 4
2.1 Definition of Baseline Characteristics 4
2.2 Analysis of Baseline Characteristics 4
3.0 Primary and Key Secondary Outcomes 6
3.1 Definition of the Primary Outcome Measure 6
3.2 Analysis of the Primary Outcome Measure 6
3.3 Supportive Analyses of the Primary Outcome Measure 7
3.3.1 BUP-XR Disruption 7
3.3.2 Model Fit 7
3.3.3 Inclusion of Covariates 7
3.4 Definition of Mother Key Secondary Outcome Measure 7
3.5 Analysis of the Mother Key Secondary Outcome Measure 8
3.6 Supportive Analyses of the Mother Key Secondary Outcome Measure 8
3.7 Definition of Infant Key Secondary Outcome Measures 8
3.7.1 Use of opioid medication for NOWS 8
3.7.2 NOWS Severity 8
3.8 Analyses of Infant Key Secondary Outcome Measures 8
3.8.1 Use of opioid medication for NOWS 8
3.8.2 NOWS Severity 8
3.9 Supportive Analyses of the Infant Key Secondary Outcome Measures 9
3.9.1 Inclusion of all BUP-XR infants 9
3.9.2 Inclusion of covariates 9
4.0 Secondary Outcomes 10
4.1 Definition of the Secondary Outcome Measures 10
4.1.1 Mother Secondary Outcomes 10
4.1.2 Infant Secondary Outcomes 11
4.2 Analyses of the Secondary Outcome Measures 12
4.3 Supportive Analyses of Secondary Outcome Measures 12
4.3.1 Medication Disruption 12
4.3.2 Inclusion of Covariates 12
5.0 Safety Outcomes 13

5.1 Adverse Events 13
5.2 Serious Adverse Events 13
5.3 Concomitant Medication 14
5.4 Opioid Overdoses 14
5.5 Injection Site Reactions 14
5.6 Hospital Anxiety and Depression Scale 14
5.7 Other Mother Safety Outcomes 15
5.8 Infant Sedation 15
5.9 Other Infant Safety Outcomes 16
5.10 Analyses of the Safety Measures 17
5.10.1 Overview 17
5.10.2 The Analysis of Safety Outcomes 17
5.11 Supportive Analyses of the Safety Measures 17
5.11.1 Medication Disruption 17
6.0 Appendix 1: Changes to Infant Secondary Outcomes 18
7.0 Appendix 2: Table Shells 19
8.0 Appendix 3: Supplemental Tables 24

1.0 Introduction

1.1 Table of Abbreviations

Abbreviation	Definition
AE	Adverse Event
APNCU	Adequacy of prenatal care utilization
AIC-C	Corrected Akaike Information Criterium
ASQ-3	Ages and Stages Questionnaire, Third Edition
BUP-SL	Sublingual buprenorphine; refers to both with and without naloxone
BUP-XR	Refers to weekly CAM2038 (Brixadi®) during pregnancy and both weekly and monthly CAM2038 (Brixadi®) after delivery unless otherwise specified
CTN	Clinical Trials Network
EGA	Estimated gestational age
HADS	Hospital Anxiety and Depression Scale
ITT	Intent-to-Treat
NAS	Neonatal Abstinence Syndrome
NIDA	National Institute on Drug Abuse
NOWS	Neonatal Opioid Withdrawal Syndrome
SAE	Serious Adverse Event
SOWS	Short Opiate Withdrawal Scale
SUD	Substance Use Disorder
TLFB	Timeline Followback
UDS	Urine drug screen

1.2 Scope of This Document

This document is a detailed plan for the analyses to be reported in the primary outcome paper for CTN-0080. The analyses are grouped as definition and summary of:

- Baseline characteristics
- The primary outcome
- The three key secondary outcomes
- The fifteen secondary outcomes
- Safety Outcomes

1.3 Changes from the Original SAP

This Statistical Analysis Plan (SAP) represents a change from the SAP created pre-database lock. This revised SAP was finalized for every updated analysis before the statistical testing was performed. The key changes to the SAP from the pre-database-lock version are:

1. Specifying the use of Bonferroni correction to correct for multiple comparisons.
2. Adjusting the analytic approach to the infant key secondary outcome measure due to zero inflation. The original outcome was days of opioid treatment calculated including all infants. However, most infants (71.8%) did not require opioid treatment resulting in 71.8% of values being zero. To address the issue, the outcome was divided into two outcomes: a) treated with opioids (binary-yes/no) and b) for the infants getting opioid treatment, the number of days of opioid treatment.
3. Adjustments to several of the infant secondary outcomes based on data limitations. These changes are described in Appendix 1.

1.4 Overview of Statistical Analyses

1.4.1 General Overview

Unless otherwise stated, all analyses in this document compare treatment cohorts (BUP-XR vs. BUP-SL) using the available data from all randomized (ITT) participants. The percent of opioid-negative UDSs during pregnancy (primary outcome) and during postpartum (a key secondary outcome) will be tested for the non-inferiority of BUP-XR relative to BUP-SL as described in the study protocol. All other statistical tests will be two-tailed tests for a significant difference between treatment cohorts. Calculations for all summary statistics and statistical tests were performed using SAS version 9.4.

1.4.2 Significance Testing and Multiplicity

Bonferroni's principle was used to set the family-wise alpha level. The three key secondary analyses each had an alpha of $0.05/3 \approx 0.0167$, and the fifteen secondary analyses were each tested at an alpha of $0.05/15 \approx 0.0033$. The safety analyses were interpreted with an alpha of 0.05 to allow the detection of medication differences for adverse outcomes.

.

1.4.3 The Pregnancy and Postpartum Study Phases

Unless stated otherwise, for the purposes of analysis, a participant mother's pregnancy phase begins the day after randomization has occurred and ends the day before delivery. Unless stated otherwise, a participant mother's postpartum phase begins the day after her hospital delivery discharge due to the limited ability to collect data while participants were in the hospital for delivery and ends 365 days after delivery. These definitions were defined a priori.

1.4.4 Overview of Regression Analyses

Regressions will be mixed-model regressions with treatment cohort being the covariate of interest and random variables including site and whether the estimated expected gestation age (EGA) was less than 19 weeks (yes / no) unless the data will not support the model (e.g., the G matrix is not positive definite, etc.). If the data do not support the model, then site and EGA will be treated as fixed effects. The same regression approach (i.e., treating site and EGA as random vs fixed) will be utilized for both non-inferiority analyses. To standardize the approach for the other outcomes, treating site and EGA as fixed effects will be used for all outcomes if the data fail to support a random effects model for any of the outcomes. Fixed nuisance covariates will include the outcome's baseline value if it has one. For all regressions except the non-inferiority regressions, if either of the random/fixed covariates is not significant (with alpha of 0.05), the regression is rerun with the nonsignificant covariate(s) removed.

As indicated in the sections below, some regressions will include discretionary fixed covariates selected from a list using the Corrected Akaike Information Criterion (AIC-C). This automated selection process involves running separate regressions with every possible set of discretionary covariates and choosing the set with the optimal AIC-C value. For mixed model regressions, the possible sets of covariates are tested treating all covariates as fixed because using the complete mixed model on all possible sets of discretionary variables requires more computing power than we have available.

Each binary outcome will be regressed using a logistic mixed model regression; each ordered categorical outcome will be regressed using a cumulative logistic mixed model regression; each count outcome will be regressed using a mixed model negative-binomial regression*, and all other outcomes will be regressed using mixed model linear regressions.

* The response variable in negative binomial regression must have a minimum of zero. If a count outcome has a minimum of one, the response variable used in the regression will be the outcome minus one.

1.4.5 Table shells

Shells for tables to be included in the main body of the primary outcomes paper are provided in Appendix 2.

1.4.6 Medication Characterization

Medication dosing (e.g., dose, formulation, etc.) will be characterized during the pregnancy and postpartum phases (see Table S1 in Appendix 3). In addition, a table characterizing medication dosing for participants impacted by the BUP-XR medication supply disruption will be generated (see Table S2 in Appendix 3).

2.0 Summary of Baseline Characteristics

2.1 Definition of Baseline Characteristics

The data for the baseline characteristics came from:

PhenX Tier 1: The PhenX Tier 1 of the Substance Abuse and Addiction core was used to collect information on demographic characteristics (e.g., sex, age, ethnicity, etc.) and information on recent and lifetime use of tobacco, alcohol, and other substances.

The DSM-5 Checklist: The DSM-5 Checklist is a semi-structured, interviewer-administered instrument that was used to assess for DSM-5 substance use disorders including: opioid, alcohol, amphetamine, cocaine, cannabis, and sedative. It will be completed during screening/ baseline.

2.2 Analysis of Baseline Characteristics

Analysis results will include a baseline characteristics table which summarizes the following characteristics taken from the study's screening survey:

- Age in years (numeric measure)
- Hispanic (yes / no) (binary measure)
- Race (black, white or other) (categorical measure))
  (black, white, or other – give details on other)
- Estimated gestational age (numeric measure)
- High school diploma, GED or less (yes /no) (binary measure)
- Employed (yes / no) (binary measure)
- Married (yes / no) (binary measure)
- The UDS at screening pos. for nicotine (yes / no) (binary measure)
  (the UDS is positive if total Cotinine metabolites ≥ 50 ng/ml.)
- The UDS at screening positive (yes / no) for each of the following (binary measure):
  - Amphetamine (500 ML)
  - Barbiturates (300 ML)
  - Buprenorphine (10 ML)
  - Benzodiazepines (300 ML)
  - Cocaine (150 ML)
  - MDMA (500 ML)
  - Methamphetamine (500 ML)
  - Methadone (300 ML)
  - Opiates (300 ML)
  - Oxycodone (100 ML)

- Phencyclidine (25 ML)
- THC (50 ML)
- Cotinine (50 ML)
- Ethyl glucuronide (500 ML)
- Fentanyl (20 ML)

Note that only substances with at least 5% of randomized participants are positive are included. The cutoffs for a positive UDS are noted in parentheses.

- Taking prescribed BUP-SL at screening (yes / no) (binary measure)

- Any self-report of injection illicit opioid use within the past 30 days per Timeline Followback at baseline (yes / no) (binary measure)

- Past 12-month DSM-5 SUD diagnosis (yes / no) for each of the following (binary measure):

  - alcohol
  - amphetamine (including methamphetamine)
  - cannabis
  - cocaine
  - sedative/hypnotic/anxiolytic

For each binary and categorical measure, the table will contain counts and percents over all randomized participants, counts and percents by treatment cohort, and the result of a Fisher's Exact test for cohort difference. For each numeric measure, the table will contain the mean with standard deviation over all randomized participants, the mean with standard deviation for each treatment cohort and the results of a Wilcoxon test for cohort difference. Where the estimated cohort variances differ, the Wilcoxon will be replaced with a Student t. All tests will have an alpha of 0.05.

3.0 Primary and Key Secondary Outcomes

3.1 Definition of the Primary Outcome Measure

The primary outcome was the proportion of illicit opioid-negative urine samples during the pregnancy phase, assessed by urine drug screens (UDSs). Opioids tested for by the UDSs were fentanyl, opiates, methadone, and oxycodone. In the case that a participant reported a prescribed medication which would cause the UDS to indicate a positive test, the urine sample was considered negative for illicit opioids for the prescribed opioid(s).

For the main analysis of the primary outcome, missing urine screening results were imputed as positive for illicit opioids. The number of UDSs expected for each participant differed based on the length of the pregnancy, thus the number of potentially imputed UDSs also differed for each participant.

3.2 Analysis of the Primary Outcome Measure

The primary outcome measure was analyzed for the ITT population.

As noted previously, this primary outcome measure was based on urine testing. It was operationalized as the percent of expected UDSs that are negative for illicit opioids. One UDS was expected at the time of each weekly Medication Check Visit between randomization and delivery – a missing UDS was imputed as positive. The number of expected UDSs was dependent on the length of time between randomization and delivery, thus we operationalized this primary outcome measure as the percent of expected UDSs that are illicit opioid-negative. The non-inferiority design results in the following hypotheses where μ_A is the mean in arm A and Δ the non-inferiority margin:

$$H_0(BUP\text{-}XR \text{ } is \text{ } inferior \text{ } to \text{ } BUP\text{-}SL): \mu_{XR} - \mu_{SL} \leq -\Delta$$

$$H_1(BUP\text{-}XR \text{ } is \text{ } non\text{-}inferior \text{ } to \text{ } BUP\text{-}SL): \mu_{XR} - \mu_{SL} > -\Delta$$

To evaluate non-inferiority at the 2.5% significance level, the two-sided 95% confidence interval for the treatment effect, $\mu_{XR} - \mu_{SL}$, was calculated using a mixed effects model where treatment arm was a fixed effect and two randomization stratification factors (site, EGA) were random effects. If the lower limit of the confidence interval is greater than -0.15 (i.e., Δ=0.15), we would reject the null hypothesis and conclude non-inferiority of BUP-XR to BUP-SL. SAS code to run this model is as follows:

```
proc mixed data = prim;
    class treat (ref = '0') site EGA;
    model outcome = treat;
    random site EGA / type = VC;
    lsmeans treat / diff cl;
run;
```

where `prim` is the primary analysis dataset; `site`, *EGA*, are the stratification factors; `outcome` is the percent of expected UDSs during pregnancy which are negative for illicit opioids which takes on values [0, 1] and `treat` is the treatment group with `treat` = 1 for BUP-XR and `treat` = 0 for BUP-SL. The effect of interest is estimated using the `lsmeans` statement and the treatment group indicator, `treat`.

If the null hypothesis was rejected, superiority of BUP-XR to BUP-SL would be considered. This involves examining the two-sided 95% confidence interval for the treatment effect, $\mu_{XR} - \mu_{SL}$. If the lower limit is above zero, then we would conclude that the extended-release formulation is superior to the sublingual formulation. With this planned order of testing, there was no need to adjust for multiplicity.

BUP-XR (BUP-XR) Medication Disruption

Participants assigned to BUP-XR who were affected by the BUP-XR disruption (i.e., when BUP-XR supply was temporarily not available) had the option to transition to study-provided BUP-SL or non-study medication (e.g., Sublocade® or non-study BUP-SL). To account for this medication disruption in the primary outcome analysis, we truncated use of UDS results for BUP-XR-randomized participants at 7 days after the participant's last weekly BUP-XR dose because each weekly dose is expected to cover an average of 7 days. That is, no UDS results would be included in the primary outcome pregnancy UDS analysis after participants were no longer covered by the weekly study medication. If a participant enrolled after the supply disruption ended or was enrolled but was in the postpartum phase prior to the disruption, all their pregnancy phase data was included in this primary outcome analysis. All pregnancy phase data from the participants randomized to BUP-SL were included in this analysis because there was no disruption in the BUP-SL medication supply.

Sensitivity analyses are detailed in the next section

3.3 Supportive Analyses of the Primary Outcome Measure

3.3.1 BUP-XR Disruption

As noted in the definition of the primary outcome measure (Section 3.1), for the main analysis of the primary outcome, missing urine results were imputed as positive for illicit opioids. Two sensitivity analyses were performed for BUP-XR supply disruption. In the first, the primary outcome analysis was completed with all data regardless of BUP-XR disruption and missing data were imputed as positive. In the second, the UDS results collected post-disruption for participants impacted by the BUP-XR disruption were included but UDS samples missing post-disruption were not imputed as being positive for illicit opioids.

3.3.2 Model Fit

If the distributional assumptions involved in the mixed effects model were not met, alternative non-parametric methods were considered for this modelling such as quantile regression.

3.3.3 Inclusion of Covariates

For the primary outcome, covariates for secondary modeling in addition to site will be explored including 1) the use of other substances at baseline (i.e., amphetamines, barbiturates, benzodiazepines, cocaine, MDMA, methamphetamine, phencyclidine, THC, cotinine, and ethyl glucuronide), which can impact both treatment adherence and illicit opioid use; and 2) route of illicit opioid use (any intravenous vs. no intravenous). Use of other substances will be determined from the baseline UDS results. Intravenous illicit opioid use will be determined from the Timeline Followback (TLFB) form collected at screening. If any intravenous illicit opioid use was noted during the 30 days of collected TLFB, the variable will be coded as yes, otherwise it will be coded as no.

3.4 Definition of Mother Key Secondary Outcome Measure

The mother key secondary outcome was the proportion of illicit opioid-negative urine samples during the postpartum phase, assessed by UDSs. Illicit opioid abstinence postpartum was determined in a similar fashion to illicit opioid abstinence during pregnancy (see Section 3.1). It should be noted that urine samples collected at the first expected visit postpartum were not included in this analysis as opioids may be prescribed post-delivery. Missing urine samples were imputed as positive for illicit opioids, with the exception of the first expected visit postpartum.

3.5 Analysis of the Mother Key Secondary Outcome Measure

The analytic approach followed the methods for testing the primary outcome. If a participant was affected by the BUP-XR medication supply disruption in the pregnancy phase, they were excluded from the postpartum analysis because all urine drug screen results were excluded after BUP-XR study disruption. If the disruption started during the postpartum phase, we truncated BUP-XR participants at either 7 days (for those receiving weekly BUP-XR) or 28 days (for those receiving BUP-XR monthly) after the medication disruption began for the participant. If a participant was not randomized until after the disruption ended, all their postpartum phase data were included in the analysis. All postpartum phase data from the participants randomized to BUP-SL were included in this analysis because there was no disruption in the BUP-SL medication supply.

3.6 Supportive Analyses of the Mother Key Secondary Outcome Measure

Two sensitivity analyses were performed for BUP-XR supply disruption. In the first, the key secondary outcome analysis was completed with all the data regardless of BUP-XR disruption with missing urine results counted as positive. In the second, the UDS results collected post-disruption for participants impacted by the BUP-XR disruption were included but UDS samples missing post-disruption were not imputed as being positive for illicit opioids.

3.7 Definition of Infant Key Secondary Outcome Measures

As explained in section 1.3, the analytic approach to the infant key secondary outcome measure was adjusted due to zero inflation. To address the issue, the outcome was divided into two outcomes: a) treated with opioids (binary-yes/no) and b) for the infants getting opioid treatment, the number of days of opioid treatment.

3.7.1 Use of opioid medication for NOWS

The use of opioid medication to treat NOWS (no/yes) was abstracted from the medical record.

3.7.2 NOWS Severity

For infants requiring opioid medication to treat symptoms, NOWS severity was assessed as total days of opioid treatment during the hospital stay which is a definition that has been used in past research. This outcome was abstracted from the medical record.

3.8 Analyses of Infant Key Secondary Outcome Measures

3.8.1 Use of opioid medication for NOWS

Opioid treatment for NOWS (yes /no) will be regressed as a binary outcome using a mixed model logistic regression as described in the Overview of Regression Analyses in section 1.4.4. To account for the medication disruption, infants in the BUP-XR group were excluded if their mothers missed any BUP-XR medication in the pregnancy phase due to the medication supply disruption.

3.8.2 NOWS Severity

Number of opioid treatment days will be regressed as a count variable using a mixed model negative binomial regression as described in the Overview of Regression Analyses in section 1.4.4. To account for the medication disruption, infants in the BUP-XR group were excluded if their mothers missed any BUP-XR medication in the pregnancy phase due to the medication disruption.

3.9 Supportive Analyses of the Infant Key Secondary Outcome Measures

3.9.1 Inclusion of all BUP-XR infants

As a sensitivity analysis, the outcomes will be re-analyzed to include all BUP-XR infants. The statistical methods described in Section 3.8 were followed.

3.9.2 Inclusion of covariates

The regressions for both outcomes were repeated but modified to include fixed nuisance covariates that were using AIC-C. The covariates considered included:

- Mother-related covariates:
  - % nicotine (i.e., cotinine)-positive UDS's
    (Include all UDS's before end-of-pregnancy including the screening UDS.)
  - % non-nicotine-substance-positive UDS's
    (Include all UDS's before end-of-pregnancy including the screening UDS.)
  - Exposure to psychiatric medications (yes / no)

- Infant-related covariates:
  - Breastfeeding while in hospital (yes / no)
  - Finnegan for scoring NOWS (yes / no)
  - Eat-Sleep-Console (yes / no)

- Hospital-related covariates:
  - Minimum days of observation for NOWS / NAS

The procedure for selecting nuisance covariates using AIC-C is explained in the Overview of Regression Analyses in section 1.4.4.

4.0 Secondary Outcomes

4.1 Definition of the Secondary Outcome Measures

4.1.1 Mother Secondary Outcomes

Note: All Mother secondary outcomes, except for Kotelchuck's Adequacy of Prenatal Care Utilization (APNCU), were analyzed separately for the pregnancy and postpartum phases.

1. BUP Medication Adherence (numeric)

Adherence to BUP treatment during pregnancy was computed as the number of days of adherence divided by the number of expected days of treatment. Expected days of treatment during pregnancy are the days between randomization and date of admission for labor and delivery for BUP-SL participants and the days between randomization and date of admission for labor and delivery that were not affected by the medication disruption for the BUP-XR participants. Postpartum adherence to BUP treatment was computed as the number of days of adherence post-hospital-discharge divided by the number of expected days of treatment during postpartum. The expected days of treatment during postpartum were post-hospital-discharge days in the 12-month postpartum phase for BUP-SL participants and the number of post-hospital-discharge days that were not affected by the medication disruption in the 12-month postpartum phase for the BUP-XR participants.

- Study BUP-XR: will be based on study records. For BUP-XR, the receipt of a weekly injection will be counted as 7 days of adherence. If a particular day is covered by two injection windows (e.g., injections on study day 7 and study day 13, where study day 13 overlaps the two seven-day windows), the overlapping adherent day will only be counted once. For monthly injections, a participant will be considered as adherent for 28 days and overlapping intervals are handled similarly as for the weekly injections.
- Study BUP-SL: will be based on 1) study records showing that BUP-SL was dispensed to the participant (i.e., BUP-SL Dispensing documentation); 2) self-reported adherence will be assessed at the weekly Medication Check Visits; and 3) UDSs positive for buprenorphine/norbuprenorphine. A participant will be considered adherent if 1) BUP-SL was dispensed, and 2) they self-report adherence unless there is a urine result that is negative for buprenorphine/norbuprenorphine in which case the day on which the urine sample was taken and the six days preceding that day were scored as not adherent.

In cases where participants discontinued study medication to transfer to an alternative treatment (e.g., methadone, extended-release naltrexone), a score of 0 days of adherence was given for the time period post discontinuation. The rationale for not "crediting" receipt of methadone or extended-release naltrexone as adherence is that a switch to an alternative medication is an indicator that assigned BUP-XR / BUP-SL was, in some way, ineffective for the participant and the score of 0 is consistent with the lack of effectiveness. In cases where participants discontinue study medication to transfer to BUP from an alternative provider or were provided non-study BUP during hospital stays or incarceration, a release of information was obtained, and adherence was based on clinic/pharmacy records (Sublocade®) or, for BUP-SL, on clinic/pharmacy records along with participant self-report and study UDSs if available. For the case where clinic/pharmacy records could not be obtained for any reason, the participant was considered non-adherent. If participants dropped out of the study prematurely, they were considered non-adherent for all days after drop-out. In the case of intermittent missing UDSs (e.g., UDSs not collected for any reason but the participant did not drop out), adherence was based on study/clinic/pharmacy records and self-report.

2. Drug and Alcohol Abstinence (numeric)

Drug and alcohol abstinence during pregnancy and postpartum are secondary outcomes and will be assessed in a fashion similar to the assessment of illicit opioid abstinence, but rather than being restricted to illicit opioid

use, will include alcohol and other drugs of abuse (but exclude cotinine). Missing urine samples were imputed as positive.

3. The Opioid Craving Scale (numeric)

Craving was assessed with the Opioid Craving Scale (OCS), which was utilized in CTN-0030 and shown to have predictive validity. The total score was calculated by averaging the scores from three visual analogue scales which assess craving, cue-induced craving, and likelihood of using. If any of the three components were missing, the total score was missing. The total score can range from 0 to 10 with higher scores indicating greater craving.

4. Adequacy of Prenatal Care Utilization (ordered categorical)

Kotelchuck's Adequacy of Prenatal Care Utilization (APNCU) index, a well-established index, was used to assess the adequacy of prenatal care. The APNCU includes two indices that are combined to obtain a total score:

- Timing of prenatal care initiation scored in 4 categories [EGA months: 1) 1 and 2; 2) 3 and 4; 3) 5 and 6; and 4) 7 to 9].
- Ratio of observed to expected visits based on the length of time between the first prenatal care visit and delivery, scored in 4 categories: 1) Inadequate (received less than 50% of expected visits); 2) Intermediate (50%-79%); 3) Adequate (80%-109%); 4) Adequate Plus (110% or more). The information for the APNCU will be derived from medical records.

The % of expected visits attended prior to study randomization was included as a baseline covariate in analyses.

5. The Short Opiate Withdrawal Scale (SOWS)-Gossop (numeric)

The SOWS-Gossop was used to measure opioid withdrawal symptoms. The SOWS-Gossop, a self-administered 10-item scale rated from 0 (none) to 3 (severe), is a validated instrument with good reliability. The total score can range from 0-30, with higher scores indicating greater withdrawal symptoms

4.1.2 Infant Secondary Outcomes

Other NOWS-related Outcomes

Three additional NOWS-related outcomes were abstracted from the medical record:

1. Infant hospital length of stay defined as the infant's age, in days, at discharge (count)
2. For infants for whom Finnegan-Modified scoring was used, peak score (numeric)
3. For infants treated with morphine who had total dose data, total mg of morphine administered for NOWS (numeric)

Discharge Outcomes

Discharge outcomes abstracted from the medical record included the binary outcomes of: infant discharged in maternal custody (yes/no) and an open case with child protective services (yes/no).

Infant Development

The 12-month version of the Ages and Stages Questionnaire, third edition (ASQ-3) was used to screen for developmental issues in the infants. The ASQ-3 is a validated, parent-administered screen used throughout the world and deemed appropriate for assessing infants exposed to opioids in utero. The outcome of interest was the binary outcome of whether the infant scores below the cut-off (yes / no) for the 12-month ASQ-3.

4.2 Analyses of the Secondary Outcome Measures

The main analyses for the secondary outcome measures include all data (i.e., ignoring medication disruption). Each binary outcome will be regressed using a logistic mixed model regression; each ordered categorical outcome will be regressed using a cumulative logistic mixed model regression; each count outcome will be regressed using a mixed model negative-binomial regression, and all other outcomes will be regressed using mixed model linear regression. The Overview of Regression Analyses in section 1.4.4 explains in full how these regressions will be performed.

4.3 Supportive Analyses of Secondary Outcome Measures

4.3.1 Medication Disruption

For mother secondary outcome measures, sensitivity analyses excluding data based on medication disruption will only be conducted if significant differences are found between the main and medication-disruption-related sensitivity analyses for the primary and mother key secondary outcome. For infant secondary outcome measures, sensitivity analyses excluding data based on medication disruption will only be conducted if significant differences are found between the main and medication-disruption-related sensitivity analyses for the infant key secondary outcomes.

4.3.2 Inclusion of Covariates

The regressions for NOWS-related outcomes were repeated but modified to include fixed nuisance covariates that were using AIC-C. The potential covariates included:

- Mother-related covariates:
    - % nicotine (i.e., cotinine)-positive UDSs
    - % non-nicotine-substance-positive UDSs
    - Exposure to psychiatric medications (yes / no)

- Infant-related covariates:
    - Any breastfeeding while in hospital (yes / no
    - Finnegan for scoring NOWS (yes / no)
    - Eat-Sleep-Console (yes / no)

- Hospital-related covariates:
    - Minimum days of observation for NOWS / NAS

The procedure for selecting nuisance covariates using AIC-C is explained in the Overview of Regression Analyses in section 1.4.4.

5.0 Safety Outcomes

Safety outcomes include adverse events, serious adverse events, injection site abnormalities, opioid-involved overdoses, fetal outcomes, maternal delivery safety outcomes, birth and neonatal outcomes, infant sedation, medical complications, and the Hospital Anxiety and Depression Scale (HADS).

5.1 Adverse Events

Adverse events (AEs) are any untoward medical occurrence in humans, whether or not considered study medication related which occurs during the conduct of a clinical trial. Any change from baseline in clinical status, ECGs, lab results, x-rays, physical examinations, etc., that is considered clinically significant by the study medical clinician are considered AEs. A suspected adverse reaction is any adverse event for which there is a reasonable possibility that the study medication caused the adverse event. A reasonable possibility implies that there is evidence that the study medication caused the event. Infant AEs are not collected, except for deaths, major congenital anomalies, and SAEs in breastfed infants. Adverse reaction is any adverse event caused by the study medication.

All adverse events will be summarized by system organ class (SOC) and preferred term (PT) using MedDRA® dictionary version 27.1. Adverse events will be presented as the number and proportion of participants experiencing at least one incidence of each event and will be presented overall and by randomized treatment group. The number of participants experiencing each type of AE will be compared between treatment arms. The AE tables will include separate sections for the pregnancy phase and the postpartum phase.

The AEs to be analyzed are listed in Table 4 of Appendix 2.

5.2 Serious Adverse Events

An event is a serious adverse event, serious suspected adverse reaction or serious adverse reaction if, in the view of either the study medical clinician or sponsor, it:

1. Results in death: A death occurring during the study or which comes to the attention of the study staff during the protocol-defined follow-up period, whether or not considered caused by the study medication, must be reported.
2. Is life-threatening: Life-threatening means that the study participant was, in the opinion of the medical clinician or sponsor, at immediate risk of death from the reaction as it occurred and required immediate intervention.
3. Requires inpatient hospitalization or prolongation of existing hospitalization.
4. Results in persistent or significant incapacity or substantial disruption of the ability to conduct normal life functions.
5. Is a congenital abnormality or birth defect.
6. Is an important medical event that may not result in one of the above outcomes but may jeopardize the health of the study participant or require medical or surgical intervention to prevent one of the outcomes listed in the above definition of serious event.

In this trial, events that did not require SAE reporting for mothers included: 1) Hospitalization for labor & delivery unless resulting in ICU admission; 2) Prolongation of the hospital stay for delivery unless greater than 4 days for vaginal and 7 days for C-section deliveries; 3) Admission to a hospital for elective surgery or pre-scheduled diagnostic tests. A summary of treatment emergent serious adverse events using proportion of participants will be provided by treatment arm for both phases. Proportions will be calculated at the MedDRA®

preferred term level, at the MedDRA® SOC level, and for participants with at least one treatment emergent adverse event. The number of participants experiencing each type of SAE will be compared between treatment arms. Tables will include sections for the pregnancy phase and the postpartum phase.

Infant death, congenital anomalies in all infants and other infant SAEs in breastfed infants are reportable and will be summarized as noted for the SAEs. No sensitivity analyses will be performed.

During the course of the trial, SAE reporting was modified to include overdoses requiring naloxone rescue at the request of Braeburn.

The SAEs to be analyzed are listed in Table 4 of Appendix 2.

5.3 Concomitant Medication

Concomitant medications will be coded using the World Health Organization (WHO) Drug Dictionary (version GLOBALC3Sep24). Summaries by treatment arm will be presented by Anatomical Therapeutic Chemical (ATC) Class level 1 and ATC Class level 2 and will include the number and percentage of participants receiving each drug class during the safety window. The safety window begins at the first dose date and ends either 7 days after the last BUP-SL dose, 7 days after the last weekly BUP-XR dose, or 28 days after the last monthly BUP-XR dose. The concomitant medications to be analyzed are listed in Table S3 of Appendix 3.

5.4 Opioid Overdoses

Participant self-report of opioid overdose was collected during the pregnancy and postpartum phases. As in past research, naloxone and/or emergency medical care (i.e., ED visit, hospitalization) to intervene is required for a reported overdose to be scored as an overdose. Occurrence of an overdose (yes/no) during the pregnancy and postpartum phases will be analyzed for treatment group differences.

5.5 Injection Site Reactions

Participants were asked to immediately report any injection site reactions to study staff for evaluation, monitoring, and possible referral, as needed. For weekly injections, the site of the last BUP-XR injection was examined at the weekly Medication Check Visits. For monthly injections, the BUP-XR injection site was examined at the weekly Medication Check Visit following the visit during which the injection was given. Injection site abnormalities will be summarized by presenting the number of abnormalities, number of participants experiencing abnormalities, type of abnormality, and the severity of the abnormality. The table shell is provided in Appendix 3 (Table S4).

5.6 Hospital Anxiety and Depression Scale

The HADS was collected at Screening, Week 3, and then monthly during the pregnancy phase. It was collected at Months 1, 3, 6, 9, and 12 during the postpartum phase. There are separate scores for anxiety and depression each of which range from 0 to 21. The outcomes of interest are:

- Average HADS total scores during pregnancy phase (numeric):
    - HADS Anxiety score

- HADS Depression score

- Average HADS total scores during postpartum phase (numeric):
  - HADS Anxiety score
  - HADS Depression score

5.7 Other Mother Safety Outcomes

Other mother safety outcomes extracted from the medical record included:

- Primary cesarian (yes / no) (binary)

- Abnormal fetal presentation at delivery (yes / no) (binary)

- Medical complications during labor (yes / no) (binary)

- Analgesic receipt during labor or recovery (yes/no) (binary):
  - Opioid analgesic
  - Non-opioid analgesic
  - Benzodiazepines
  - Other

- Analgesic receipt during postpartum (yes/no) (binary):
  - Opioid analgesic
  - Non-opioid analgesic
  - Benzodiazepines
  - Other

- Analgesic receipt prescribed at discharge (yes/no) (binary):
  - Opioid analgesic
  - Non-opioid analgesic
  - Benzodiazepines
  - Other

5.8 Infant Sedation

Infant sedation was assessed by mother self-report on a weekly basis during the 12-month postpartum phase. Infant sedation in infants receiving breastmilk will be analyzed for three measures of sedation:

- Did not wake up for feeding (yes/no) (binary)
- Any difficulty breathing (yes/no) (binary)
- Felt limp when held (yes/no) (binary)

5.9 Other Infant Safety Outcomes

Other infant safety outcomes extracted from the medical record included:

- Live birth (yes/no) (binary)
- Estimated gestational age (EGA) in wks. at delivery (numeric)
- Head circumference in cm (numeric)
- Weight at birth in gm (numeric)
- Length at birth in cm (numeric)
- Apgar score at 1 minute (numeric)
- Apgar score at 5 minutes (numeric)
- Abnormal conditions (yes/no) (binary)
- Interventions for abnormal conditions (yes/no) (binary)
- Abnormal Conditions during hospitalization
    - Cardiac failure (yes/no) (binary)
    - Respiratory distress (yes/no) (binary)
    - Feeding problems (yes/no) (binary)
    - Sepsis (yes/no) (binary)
    - Seizure or serious neurologic dysfunction (yes/no) (binary)
    - Significant birth injury (yes/no) (binary)
    - Other (yes/no) (binary)
- Interventions for abnormal conditions
    - Resuscitation required immediately following delivery (yes/no) (binary)
    - Assisted ventilation required immediately following delivery (yes/no) (binary)
    - Assisted ventilation required for more than six hours (yes/no) (binary)
    - Newborn given surfactant replacement therapy (yes/no) (binary)
    - NICU admission (yes/no) (binary)
    - Antibiotics for suspected sepsis (binary)
    - Feeding (NG) tube inserted (binary)
- Preterm (EGA < 37 wks.) (yes / no) (binary)
- Reasons for pre-term birth
    - Fetal distress (yes/no) (binary)
    - Premature placental abruption (yes/no) (binary)
    - Maternal trauma (yes/no) (binary)
    - Other (yes/no) (binary)
- Infant discharged alive (yes / no) (binary)

5.10 Analyses of the Safety Measures

5.10.1 Overview

The main analyses for the safety measures will include all data (i.e., ignoring medication disruption). All statistical tests will have an alpha of 0.05.

5.10.2 The Analysis of Safety Outcomes

All regressions will be performed in the same manner as the main analyses for the secondary outcomes. (See section 4.2.)

5.11 Supportive Analyses of the Safety Measures

5.11.1 Medication Disruption

For mother safety measures, sensitivity analyses excluding data based on medication disruption will only be conducted if significant differences are found between the main and medication-disruption-related sensitivity analyses for the primary and mother key secondary outcome. For infant safety measures, sensitivity analyses excluding data based on medication disruption will only be conducted if significant differences are found between the main and medication-disruption-related sensitivity analyses for the infant key secondary outcomes.

6.0 Appendix 1: Changes to Infant Secondary Outcomes

The following outcomes were dropped due to data limitations:

- Use of adjunct medications (e.g., phenobarbital, clonidine) during hospitalization was dropped because the sample size was insufficient with only 10 infants having adjunct medications (3 in the BUP-XR arm and 7 in the BUP-SL arm).
- ICD-10 coding indicative of NOWS (yes/no). Infants were scored as "yes" if the medical record included an ICD-10 code of P96.1 (neonatal withdrawal symptoms from maternal use of drugs of addiction) and/or P96.2 (withdrawal symptoms from therapeutic use of drugs in newborn) within the first 10 days of life. This outcome was deemed to be unreliable because only 62% of the infants who received opioid treatment were given an ICD-10 code indicating under-coding of NOWS in infant hospital medical records.
- Medications at discharge (e.g., phenobarbital, clonidine) from the hospital was dropped because the sample size was insufficient with only 7 infants having medications at discharge (1 in the BUP-XR arm and 6 in the BUP-SL arm).

One infant outcome had its definition changed. The original outcome was defined as: "NOWS scoring assessment used and peak score". The NOWS scoring assessments used were as follows:

Method for evaluating NOWS	BUP-SL N=69	BUP-XR N=66	Total N= 135
Finnegan-Original	1 (1.4%)	5 (7.6%)	6 (4.4%)
Finnegan-Modified	27 (39.1%)	27 (40.9%)	54 (40.0%)
Eat-Sleep-Console	18 (26.1%)	12 (18.2%)	30 (22.2%)
Neonatal Withdrawal Inventory	4 (5.8%)	6 (9.1%)	10 (7.4%)
MOTHER NAS Scale	3 (4.3%)	0 (0.0%)	3 (2.2%)
Missing	15 (21.7%)	15 (22.7%)	30 (22.2%)
Other - Have formal system, not certain of name	1 (1.4%)	0 (0.0%)	1 (0.7%)
Other - Unknown	0 (0.0%)	1 (1.5%)	1 (0.7%)

In addition to the low rates with which some scoring methods were used there were many missing data points. For example, very few scores were provided for Eat-Sleep-Console. The only scoring approach with enough data to allow analysis of potential group differences was the Finnegan-Modified method; this method was used for 27 infants in each arm with a peak score provided for 22 BUP-SL infants and 19 BUP-XR infants. Thus, the definition of this outcome was modified to NOWS peak score as defined by the Finnegan-Modified assessment.

7.0 Appendix 2: Table Shells

Table 1. Baseline Characteristics of the Participants in the Intent-to-Treat Population

	BUP-XR (N=69)	BUP-SL (N=71)	Total (N=140)	P Value
Age (yr)				
Hispanic or Latina ethnicity, no. (%)				
Race, no. (%)				
White				
Black				
Other[+]				
Estimated gestational age of fetus (wk)				
High school diploma, GED, or lower education level, no. (%)[++]				
Employed, no. (%)				
Married, no. (%)				
Cotinine metabolites ≥ 50 ng/mL				
Substances for which UDS was positive for ≥ 5% of participants				
Substance 1				
Substance 2 etc.				
Taking BUP-SL at randomization				
Injection illicit opioid use at baseline				
Other DSM-5 SUDs (past 12 months), n (%)				
Alcohol				
Amphetamine				
Cannabis				
Cocaine				
Sedatives				

[+]Race and ethnic group were reported by the participants. The “other” category included [list]

[++]Of 136 participants reporting

Table 2 – Maternal Primary, Secondary, and Safety Outcomes as a function of Treatment

Outcome	BUP-XR (N=69)	BUP-SL (N=71)	Odds Ratio CI [1]	P Value (for testing)
**Primary outcome-Pregnancy**				
Illicit opioid-negative urine samples[2]	0.84 (0.22)	0.74 (0.28)		0.05
**Key secondary outcome-Postpartum**				
Illicit opioid-negative urine samples[3]	0.61 (0.33)	0.60 (0.32)		0.05/3
**Other secondary outcomes**				
BUP Adherence-pregnancy				0.05/15
BUP Adherence-postpartum				0.05/15
Drug and alcohol abstinence[4]-pregnancy				0.05/15
Drug and alcohol abstinence[4] -postpartum				0.05/15
Opioid craving scale-pregnancy				0.05/15
Opioid craving scale-postpartum				0.05/15
Adequacy of Prenatal Care Utilization[5]				0.05/15
Inadequate				
Intermediate				
Adequate				
Adequate Plus				
Short Opiate Withdrawal Scale -pregnancy				0.05/15
Short Opiate Withdrawal Scale -postpartum				0.05/15
**Mother safety outcomes**				
Primary cesarean– no. (%)				0.05
Abnormal fetal presentation during delivery – no. (%)				0.05
Medical complications during labor-no. (%)				0.05
Analgesic receipt				
During labor or delivery				
Opioid analgesic				0.05
Non-opioid analgesic				0.05
Benzodiazepines				0.05
Other				0.05
During postpartum				
Opioid analgesic				0.05
Non-opioid analgesic				0.05
Benzodiazepines				0.05
Other				0.05
Prescribed at time of discharge				
Opioid analgesic				0.05
Non-opioid analgesic				0.05
Benzodiazepines				0.05
Other				0.05
Pregnancy Phase				
Opioid Overdose – no. (%)				0.05
HADS Depression total score				0.05
HADS Anxiety total score				0.05
Postpartum Phase				
Opioid Overdose – no. (%)				0.05
HADS Depression total score				0.05
HADS Anxiety total score				0.05

[1] CI matches the alpha for statistical testing (e.g., 95% CI for α =.05; 98.3% for α =0.017, etc.);[2] Data is truncated at 7 days (weekly dosing) after the medication disruption began for the 2 participants affected by the BUP-XR disruption; [3] Data is truncated at 7 days (weekly dosing) or 28 days (monthly dosing) after the medication disruption began for the 16 participants affected by the BUP-XR disruption; [4]Excluding cotinine; [5]Sample sizes

Table 3 – Infant Secondary and Safety Outcomes as a function of Treatment

Outcome	BUP-XR (N=66)	BUP-SL (N=69)	Odds Ratio CI[1]	P Value (for testing)
**Key secondary outcomes**				
Infants treated with opioids- no (%)[2]				0.05/3
Neonatal: Days of opioid treatment[3]				0.05/3
**Other secondary outcomes**				
Total amount of morphine for NOWS-mg[4]				0.05/15
Hospital length of stay				0.05/15
Finnegan-Modified Peak Score[5]				0.05/15
Maternal custody – no. (%)				0.05/15
Case with child protective services- no. (%)				0.05/15
Developmental issues at 12 months-no. (%)				0.05/15
**Infant safety outcomes**				
Live birth[6]– no. (%)				0.05
Head circumference[7]-cm				0.05
Weight at birth[8] - g				0.05
Length at birth[9] - cm				0.05
Gestational age at delivery – wk				0.05
Apgar score[10]				
1 min				0.05
5 min				0.05
Congenital abnormalities – no. (%)				0.05
Abnormal conditions-no. (%)				
Cardiac failure				0.05
Respiratory distress				0.05
Feeding problems				0.05
Sepsis				0.05
Seizure or serious neurologic dysfunction				0.05
Significant birth injury				0.05
Other				0.05
Interventions for abnormal conditions-no. (%)				
Resuscitation required immediately following delivery (including CPR)				0.05
Assisted ventilation required immediately following delivery				0.05
Assisted ventilation required for more than six hours				0.05
Newborn given surfactant replacement therapy				0.05
NICU admission				0.05
Antibiotics for suspected neonatal sepsis				0.05
Feeding (NG) tube inserted				0.05
Preterm, <37 wk – no. (%)				0.05
Reasons for preterm birth – no. (%)				
Fetal distress				0.05
Premature placental abruption				0.05
Maternal trauma				0.05
Other				0.05
Infant discharged alive				0.05
Infant Sedation [x] -no. (%)				
Did not wake up for feeding				0.05
Any difficulty breathing				0.05
Felt limp when held				0.05

[1] CI matches the alpha for statistical testing (e.g., 95% CI for α =.05; 98.3% for α =0.017, etc.);[2] Excludes the 2 infants from mothers impacted by medication disruption during pregnancy; [3]For infants receiving opioid treatment (BUP-XR n=19, BUP-SL n=18);

[4]Includes neonates treated with morphine who had total dose data (BUP-XR n=11; BUP-SL n=13); [5]Includes neonates scored using the Finnegan original or modified versions (BUP-XR n=28; BUP-SL n=32); [6] BUP-XR n=67; BUP-SL n=70; [7]BUP-XR n=61; BUP-SL n=67; [8]BUP-XR n=65; BUP-SL n=69; [9]BUP-XR n=63; BUP-SL n=67; [10]BUP-XR n=64; BUP-SL n=69; [x]In infants receiving breastmilk (BUP-XR n=53; BUP-SL n=55)

Table 4. Participants with Serious and Nonserious Treatment Emergent Adverse Events (TEAEs) Summarized by Treatment Arm and Study Phase

	**Mother (Pregnancy)**		**Mother (Postpartum)**		**Infant**	
	**BUP-XR** (N=69)	**BUP-SL** (N=71)	**BUP-XR** (N=67)	**BUP-SL** (N=70)	**BUP-XR** (N=67)	**BUP-SL** (N=70)
**Participants with At Least One Serious TEAE**						
Any Serious TEAE						
Medication Related						
Organized by MedDRA v27.1 System Organ Class:						
Congenital/genetic disorders						
Gastrointestinal disorders						
General disorders						
Hepatobiliary disorders						
Infections and infestations						
Injury, poisoning and procedural complications						
Investigations						
Metabolism and nutrition disorders						
Musculoskeletal and connective tissue disorders						
Nervous system disorders						
Pregnancy, puerperium and perinatal conditions						
Psychiatric disorders						
Renal and urinary disorders						
Respiratory, thoracic and mediastinal disorders						
Surgical and medical procedures						
Organized by Type:						
Congenital anomaly or birth defect						
Persistent or significant disability or incapacity						
Death						
Initial or prolonged hospitalization						
Life threatening						
Important Medical Event						

	Mother (Pregnancy)		Mother (Postpartum)		Infant	
	**BUP-XR** (N=69)	**BUP-SL** (N=71)	**BUP-XR** (N=67)	**BUP-SL** (N=70)	**BUP-XR** (N=67)	**BUP-SL** (N=70)
**Participants with At Least One Nonserious TEAE**					Not tracked in infants	
Any Nonserious TEAE						
Medication related						
Organized by Severity:<br>(Percents relate to participants with nonserious TEAE)						
Mild						
Moderate						
Severe						
Organized by MedDRA v27.1 System Organ Class:<br>(only includes classes affecting 5% or more participants)						
Gastrointestinal disorders						
General disorders and administration site conditions						
Infections and infestations						
Injury, poisoning and procedural complications						
Investigations						
Musculoskeletal and connective tissue disorders						
Nervous system disorders						
Pregnancy, puerperium and perinatal conditions						
Psychiatric disorders						
Reproductive system and breast disorders						
Respiratory, thoracic and mediastinal disorders						
Skin and subcutaneous tissue disorders						
Vascular disorders						

8.0 Appendix 3: Supplemental Tables

Table S1. Study Medication Dosing as a function of phase and treatment arm

	**Pregnancy [1,2]**	**Postpartum [3,4]**
**BUP-XR**		
**Weekly medication-no (%)**		
8 mg dose		
16 mg dose		
24 mg[5] dose		
32 mg dose		
**Monthly medication-no (%)**		
64 mg dose		
96 mg[5] dose		
128 mg dose		
**BUP-SL**		
Mono product[6] -no. (%)		
Combination product[7]-no. (%)		
Total daily dose mean (SD)		
Total daily dose-no. (%)		
< 8 mg		
8 - 11 mg		
12- 16 mg dose		
>16 mg – 24 mg dose		
> 24 mg dose		
Dosing frequency-no. (%)		
Daily		
Twice daily		
Three times or more daily		

[1]Dosing reflects the dosing closest to delivery or last dose before medication disruption for impacted participants; [2] BUP-XR n=69, BUP-SL n=71; [3] Dosing reflects the last postpartum dose or last dose before medication disruption for impacted participants; [4] BUP-XR n=59, BUP-SL n=65; [5]equivalent to 12-16 mg of BUP-SL; [6]buprenorphine only; [7]buprenorphine and naloxone

Table S2. Summary of Medication Dosing for Participants Impacted by Medication Disruption

	Pregnancy (N=2)	Postpartum (N=14)
BUP-XR participants (% impacted)		
Days BUP-XR provided pre-disruption, mean (SD)		
**Weekly medication dose at disruption-no. (%)**		
8 mg dose		
16 mg dose		
24 mg dose		
32 mg dose		
**Monthly medication dose at disruption-no. (%)**		
64 mg dose		
96 mg dose		
128 mg dose		
**Replacement medication started-no. (%)**		
BUP-SL Mono product[1] -no. (%)		
BUP-SL Combination product[2]-no. (%)		
BUP-SL starting dose, mean (SD)		
BUP-SL ending dose, mean (SD)		
Sublocade- no. (%)		
100 mg starting dose– no. (%)		
300 mg starting dose– no. (%)		
100 mg ending dose– no. (%)		
300 mg ending dose– no. (%)		
Days on replacement medication, mean (SD)		
**BUP-XR restarted-no. (%)**		
Restarted before the end of impacted phase-no. (%)		
Restarted weekly BUP-XR (%)		
Restarted monthly BUP-XR (%)		
**Weekly medication dose at restart-no. (%)**		
8 mg dose		
16 mg dose		
24 mg dose		
32 mg dose		
**Monthly medication dose at restart-no. (%)**		
64 mg dose		
96 mg dose		
128 mg dose		

[1]buprenorphine only; [2]buprenorphine and naloxone

Table S3. Concomitant Medications

	Treatment Arm		
**Anatomic Therapeutic Chemical Class 1/Anatomic Therapeutic Chemical Class 2**	**BUP-XR**	**BUP-SL**	**Total**
Number of participants randomized			
Participants with at least one concomitant medication -no. (%)			
**ALIMENTARY TRACT AND METABOLISM**			
ANTIDIARRHEALS, INTESTINAL ANTIINFLAMMATORY/ANTIINFECTIVE AGENTS			
ANTIEMETICS AND ANTINAUSEANTS			
ANTIOBESITY PREPARATIONS, EXCL. DIET PRODUCTS			
APPETITE STIMULANTS			
BILE AND LIVER THERAPY			
DRUGS FOR ACID RELATED DISORDERS			
DRUGS FOR CONSTIPATION			
DRUGS FOR FUNCTIONAL GASTROINTESTINAL DISORDERS			
DRUGS USED IN DIABETES			
MINERAL SUPPLEMENTS			
STOMATOLOGICAL PREPARATIONS			
VITAMINS			
**ANTIINFECTIVES FOR SYSTEMIC USE**			
ANTIBACTERIALS FOR SYSTEMIC USE			
ANTIMYCOBACTERIALS			
ANTIMYCOTICS FOR SYSTEMIC USE			
ANTIVIRALS FOR SYSTEMIC USE			
IMMUNE SERA AND IMMUNOGLOBULINS			
VACCINES			
**ANTINEOPLASTIC AND IMMUNOMODULATING AGENTS**			
IMMUNOSUPPRESSANTS			
**BLOOD AND BLOOD FORMING ORGANS**			
ANTIANEMIC PREPARATIONS			
ANTITHROMBOTIC AGENTS			
BLOOD SUBSTITUTES AND PERFUSION SOLUTIONS			
**CARDIOVASCULAR SYSTEM**			
AGENTS ACTING ON THE RENIN-ANGIOTENSIN SYSTEM			
ANTIHYPERTENSIVES			
BETA BLOCKING AGENTS			
CALCIUM CHANNEL BLOCKERS			
CARDIAC THERAPY			
DIURETICS			

	Treatment Arm		
**Anatomic Therapeutic Chemical Class 1/Anatomic Therapeutic Chemical Class 2**	**BUP-XR**	**BUP-SL**	**Total**
LIPID MODIFYING AGENTS			
VASOPROTECTIVES			
**DERMATOLOGICALS**			
ANTI-ACNE PREPARATIONS			
ANTIBIOTICS AND CHEMOTHERAPEUTICS FOR DERMATOLOGICAL USE			
ANTIFUNGALS FOR DERMATOLOGICAL USE			
ANTIPRURITICS, INCL. ANTIHISTAMINES, ANESTHETICS, ETC.			
CORTICOSTEROIDS, DERMATOLOGICAL PREPARATIONS			
EMOLLIENTS AND PROTECTIVES			
OTHER DERMATOLOGICAL PREPARATIONS			
**GENITO URINARY SYSTEM AND SEX HORMONES**			
GYNECOLOGICAL ANTIINFECTIVES AND ANTISEPTICS			
OTHER GYNECOLOGICALS			
SEX HORMONES AND MODULATORS OF THE GENITAL SYSTEM			
UROLOGICALS			
**MUSCULO-SKELETAL SYSTEM**			
ANTIINFLAMMATORY AND ANTIRHEUMATIC PRODUCTS			
MUSCLE RELAXANTS			
TOPICAL PRODUCTS FOR JOINT AND MUSCULAR PAIN			
**NERVOUS SYSTEM**			
ANALGESICS			
ANESTHETICS			
ANTIEPILEPTICS			
OTHER NERVOUS SYSTEM DRUGS			
PSYCHOANALEPTICS			
PSYCHOLEPTICS			
**RESPIRATORY SYSTEM**			
ANTIHISTAMINES FOR SYSTEMIC USE			
COUGH AND COLD PREPARATIONS			
DRUGS FOR OBSTRUCTIVE AIRWAY DISEASES			
NASAL PREPARATIONS			
**SENSORY ORGANS**			
OPHTHALMOLOGICALS			
OTOLOGICALS			
**SYSTEMIC HORMONAL PREPARATIONS, EXCL. SEX HORMONES AND INSULINS**			
CORTICOSTEROIDS FOR SYSTEMIC USE			

	Treatment Arm		
**Anatomic Therapeutic Chemical Class 1/Anatomic Therapeutic Chemical Class 2**	**BUP-XR**	**BUP-SL**	**Total**
PITUITARY AND HYPOTHALAMIC HORMONES AND ANALOGUES			
THYROID THERAPY			
**VARIOUS**			
ALL OTHER NON-THERAPEUTIC PRODUCTS			
ALL OTHER THERAPEUTIC PRODUCTS			
CONTRAST MEDIA			
GENERAL NUTRIENTS			
UNSPECIFIED HERBAL AND TRADITIONAL MEDICINE			

Table S4. Summary of Study Injection Site Examinations

	Grade 1 - Mild	Grade 2 - Moderate	Grade 3 - Severe	Total
Number of injections				
Injection site examinations no. (%)				
Number of abnormal injection sites- no. (%)				
Symptom experienced -no.				
Pain -no. (%)				
Itching-no. (%)				
Discharge-no. (%)				
Tenderness-no. (%)				
Erythema/Redness-no. (%)				
Swelling-no. (%)				
Induration-no. (%)				
Abscess-no. (%)				
Ulceration-no. (%)				
Necrosis-no. (%)				
Other*-no. (%)				

*Other included:
