# Supplementary material for: Extended-Release vs Sublingual Buprenorphine in Pregnancy Through 12 Months Post Partum: A Randomized Clinical Trial
Source: JAMA Intern Med. 2026 Mar 16;186(5):533–43. doi: 10.1001/jamainternmed.2026.0057 (PMC12993732; doi:10.1001/jamainternmed.2026.0057)
Supplement: Supplement 2. — eTable 1. Summary of medication dosing for participants impacted by medication disruption eTable 2. Study medication dosing as a function of phase and treatment group eTable 3. Listing of initial or prolonged hospitalizations in infants as a function of treatment group eTable 4. Listing of congenital anomalies in infants as a function of treatment group eTable 5. Study medication-related adverse events during pregnancy as a function of treatment group eTable 6. Serious and nonserious adverse events occurring during the study by MedDRA System Organ Class eTable 7. Summary of study injection site examinations eTable 8. Injection site reactions by severity and time to resolution eTable 9. Other maternal safety outcomes as a function of phase and treatment group eTable 10. Other infant safety outcomes as a function of phase and treatment group [file jamainternmed-e260057-s002.pdf]

Supplemental Online Content

Winhusen TJ, Lofwall MR, Kropp F, et al. Extended-release vs sublingual buprenorphine in pregnancy through 12 months post partum: a randomized clinical trial. *JAMA Intern Med.* Published online March 16, 2026. doi:10.1001/jamainternmed.2026.0057

eTable 1. Summary of medication dosing for participants impacted by medication disruption

eTable 2. Study medication dosing as a function of phase and treatment group

eTable 3. Listing of initial or prolonged hospitalizations in infants as a function of treatment group

eTable 4. Listing of congenital anomalies in infants as a function of treatment group

eTable 5. Study medication-related adverse events during pregnancy as a function of treatment group

eTable 6. Serious and nonserious adverse events occurring during the study by MedDRA System Organ Class

eTable 7. Summary of study injection site examinations

eTable 8. Injection site reactions by severity and time to resolution

eTable 9. Other maternal safety outcomes as a function of phase and treatment group

eTable 10. Other infant safety outcomes as a function of phase and treatment group

This supplemental material has been provided by the authors to give readers additional information about their work.

© 2026 Winhusen TJ et al. *JAMA Internal Medicine*.

eTable 1. Summary of medication dosing for participants impacted by medication disruption

	Pregnancy (N=2)	Postpartum (N=14)
Extended-release participants (% impacted)	2.9%	20.9%
Days extended-release provided pre-disruption, mean (SD)	121.5 (13.4)	300.5 (130.5)
**Weekly medication dose at disruption-no. (%)**		
8 mg dose	0 (0.0%)	2 (14.3%)
16 mg dose	0 (0.0%)	1 (7.1%)
24 mg dose	2 (100.0%)	2 (14.3%)
32 mg dose	0 (0.0%)	1 (7.1%)
**Monthly medication dose at disruption-no. (%)**		
64 mg dose		1 (7.1%)
96 mg dose		1 (7.1%)
128 mg dose		6 (42.9%)
**Replacement medication started-no. (%)**	2 (100.0%)	11[1] (78.6%)
Sublingual Mono product[2] -no. (%)	2 (100.0%)	8 (100%)
Sublingual Combination product[3]-no. (%)	0 (0.0%)	0 (0.0%)
Sublingual starting dose, mean (SD)	10.0 (2.8)	15.5 (8.7)
Sublingual ending dose, mean (SD)	12.0 (5.7)	17.0 (9.0)
Sublocade- no. (%)		4 (28.6%)
100 mg starting dose– no. (%)		1 (25.0%)
300 mg starting dose– no. (%)		3 (75.0%)
100 mg ending dose– no. (%)		3 (75.0%)
300 mg ending dose– no. (%)		1 (25.0%)
Days on replacement medication, mean (SD)	74.0 (19.8)	39.7 (37.6)
**Extended-release restarted-no. (%)**	2 (100.0%)	9 (64.3%)
Restarted before the end of impacted phase	0 (0.0%)	9 (100.0%)
Restarted weekly extended-release	1 (50.0%)	1 (11.1%)
Restarted monthly extended-release	1 (50.0%)	8 (88.9%)
Active in trial, restarted extended-release buprenorphine	2 (100%)	9 (90%)
**Weekly medication dose at restart-no. (%)**		
8 mg dose	0 (0.0%)	
16 mg dose	0 (0.0%)	
24 mg dose	1 (50.0%)	1 (11.1%)
32 mg dose	0 (0.0%)	
**Monthly medication dose at restart-no. (%)**		
64 mg dose	0 (0.0%)	1 (11.1%)
96 mg dose	1 (50.0%)	
128 mg dose	0 (0.0%)	7 (77.8%)

[1]One participant used both Sublocade® and sublingual buprenorphine during the replacement period; [2]buprenorphine only; [3]buprenorphine and naloxone

© 2026 Winhusen TJ et al. *JAMA Internal Medicine*.

eTable 2. Study medication dosing as a function of phase and treatment group

	Pregnancy [1]	Postpartum [2]
**Extended-release**	**N=69**	**N=59**
**Weekly medication-no (%)**		25 (42.4%)
8 mg dose	2 (2.9%)	4 (16.0%)
16 mg dose	8 (11.6%)	2 (8.0%)
24 mg[3] dose	18 (26.1%)	8 (32.0%)
32 mg dose	41 (59.4%)	11 (44.0%)
**Monthly medication-no (%)**		34 (57.6%)
64 mg dose		3 (8.8%)
96 mg[3] dose		9 (26.5%)
128 mg dose		22 (64.7%)
**Medication Discontinued-no (%)**	**8 (11.6%)**	**25 (37.3%)**
**Sublingual**	**N=71**	**N=65**
Mono product[4] -no. (%)	49 (69.0%)	28 (43.1%)
Combination product[5]-no. (%)	22 (31.0%)	37 (56.9%)
Total daily dose mean (SD)	18.8 (6.7)	18.2 (6.8)
**Total daily dose-no. (%)**		
< 8 mg	4 (5.6%)	5 (7.7%)
8 - 11 mg	5 (7.0%)	4 (6.2%)
12- 16 mg dose	23 (32.4%)	26 (40.0%)
>16 mg – 24 mg dose	34 (47.9%)	26 (40.0%)
> 24 mg dose	5 (7.0%)	4 (6.2%)
**Dosing frequency-no. (%)**		
Daily	10 (14.1%)	11 (16.9%)
Twice daily	34 (47.9%)	35 (53.8%)
Three times or more daily	27 (38.0%)	19 (29.2%)
**Medication Discontinued-no (%)**	**6 (8.5%)**	**23 (32.9%)**

[1]Dosing reflects the dosing closest to delivery or last dose before medication disruption for impacted participants;
[2] Dosing reflects the last postpartum dose or last dose before medication disruption for impacted participants;
[3]Equivalent to 12-16 mg of sublingual buprenorphine; [4]Buprenorphine only; [5]Buprenorphine and naloxone

© 2026 Winhusen TJ et al. *JAMA Internal Medicine*.

eTable 3. Listing of initial or prolonged hospitalizations in infants as a function of treatment group

Treatment Arm=BUP-XR

Day of Birth[1]	Onset Day[1]	Resolution / Death Day[1]	AE Description	Severity of AE	Relatedness to Study Drug	Outcome	SAE Associated With	Preferred Term	System Organ Class
86	120	122	Failure to gain weight	Grade 2 - Moderate	No	Recovered/ resolved	Initial or prolonged hospitalization	Weight gain poor	Metabolism and nutrition disorders
121	126	133	Hyperbilirubinemia	Grade 3 - Severe	No	Recovered/ resolved	Initial or prolonged hospitalization	Hyperbilirubin aemia	Hepatobiliary disorders
107	114	115	Poor Weight Gain	Grade 3 - Severe	No	Recovered/ resolved	Initial or prolonged hospitalization	Weight gain poor	Metabolism and nutrition disorders
62	67	69	hyperbilirubinemia of newborn	Grade 2 - Moderate	No	Recovered/ resolved	Initial or prolonged hospitalization	Hyperbilirubin aemia neonatal	Hepatobiliary disorders
50	64	74	Bacteremia/sepsis - Klebsiella	Grade 2 - Moderate	No	Recovered/ resolved	Initial or prolonged hospitalization	Klebsiella sepsis	Infections and infestations
50	79	94	Abnormal movements/spasms	Grade 2 - Moderate	No	Recovered/ resolved	Initial or prolonged hospitalization	Muscle spasms	Musculoskeletal and connective tissue disorders
50	79	82	choking episode	Grade 2 - Moderate	No	Recovered/ resolved	Initial or prolonged hospitalization	Choking	Respiratory, thoracic and mediastinal disorders
50	162	171	tongue laceration of baby	Grade 3 - Severe	No	Recovered/ resolved	Initial or prolonged hospitalization	Tongue injury	Injury, poisoning and procedural complications
112	114	121	urinary tract infection	Grade 3 - Severe	No	Recovered/ resolved	Initial or prolonged hospitalization	Urinary tract infection	Infections and infestations

Treatment Arm=BUP-SL

Day of Birth[1]	Onset Day[1]	Resolution / Death Day[1]	AE Description	Severity of AE	Relatedness to Study Drug	Outcome	SAE Associated With	Preferred Term	System Organ Class
138	179	182	Neonatal Abstinence Syndrome	Grade 3 - Severe	Yes	Recovered/resolved	Initial or prolonged hospitalization	Drug withdrawal syndrome neonatal	General disorders and administration site conditions

© 2026 Winhusen TJ et al. *JAMA Internal Medicine*.

Treatment Arm=BUP-XR									
Day of Birth[1]	Onset Day[1]	Resolution / Death Day[1]	AE Description	Severity of AE	Relatedness to Study Drug	Outcome	SAE Associated With	Preferred Term	System Organ Class
91	141	144	Pneumonia	Grade 3 - Severe	No	Recovered/resolved	Initial or prolonged hospitalization	Pneumonia	Infections and infestations
206	253	265	infant covid hospitalization	Grade 3 - Severe	No	Recovered/resolved	Initial or prolonged hospitalization	COVID-19	Infections and infestations
34	34	40	Urinary Retention	Grade 2 - Moderate	No	Recovered/resolved	Initial or prolonged hospitalization	Urinary retention	Renal and urinary disorders
197	205	207	Neonatal weight loss	Grade 3 - Severe	No	Recovered/resolved	Initial or prolonged hospitalization	Weight decrease neonatal	Pregnancy, puerperium and perinatal conditions
99	104	109	Hypothermia	Grade 2 - Moderate	No	Recovered/resolved	Initial or prolonged hospitalization	Hypothermia	General disorders and administration site conditions

[1] Study Day is relative to first dose (Day 1 = first dose date)

© 2026 Winhusen TJ et al. *JAMA Internal Medicine*.

eTable 4. Listing of congenital anomalies in infants as a function of treatment group*

	Maternal age	EGA @ Random-izatio,	EGA @ birth, type of delivery, infant sex	Day of Birth[1]	Onset Day[1]	Resolution / Death Day[1]	AE Description, Severity, and Relatedness to Study Drug	Preferred Term	System Organ Class	Outcome	Maternal Obstetric/Med History/Study Med Dose Exposures/Misc
**Treatment Arm=BUP SL**											
	30 years	17 weeks (w)	32w2days<br>C-section<br>Female	107	108	209	Biliary Atresia<br>Grade 3 – Severe<br>Not related	Congenital absence of bile ducts	Congenital, familial and genetic disorders<br>(CFAGD)	Recovering/ resolved with sequelae	G4P2113<br>Infant delivered early due to maternal COVID infection with respiratory failure requiring maternal intubation/hospitalization.<br>Substance History: mild amphetamine use disorder, use of alcohol, Ambien, Xanax, Valium, stimulants (cocaine, Ritalin, Adderall, cannabis, hallucinogens (ecstasy, MDMA) and tobacco.<br>Psychiatric History: Major Depressive Disorder and anxiety<br>Maintained on 4-6mg of sublingual buprenorphine through pregnancy
	30 years	27 w	38w<br>Vaginal delivery<br>Female	74	74	99	Spina bifida<br>Grade 3 – Severe<br>Not related	Spina bifida	CAFGD	Recovered/ resolved	G4P4004<br>Spina bifida diagnosed prior to delivery at 29 weeks EGA, 13 days after randomization and noted to not have prenatal vitamins or folate listed as ongoing/prior concomitant medications at the time. After delivery, infant noted to have bilateral club feet and echocardiogram showed patent foramen ovale (note: these were consider minor anomalies so were not reported as infant SAE).<br>Substance History: severe cocaine use disorder, use of Xanax, stimulants (Adderall and cocaine), cannabis, and tobacco<br>Maintained on 16 mg daily sublingual buprenorphine throughout pregnancy
	30 years	30 w	40w<br>Vaginal delivery<br>Male	56	98	-	Renal Pelviectasis<br>Grade 2 – Moderate<br>Not related	Pyelo-caliectasis	Renal and urinary disorders	Not recovered/ not resolved<br>[Infant was still receiving pediatric urology follow-	G1P0001<br>Substance history: Severe amphetamine disorder, hallucinogens (ecstasy, mushrooms, PCP), tobacco, cocaine, cannabis,

© 2026 Winhusen TJ et al. *JAMA Internal Medicine*.

	Maternal age	EGA @ Random-izatio,	EGA @ birth, type of delivery, infant sex	Day of Birth[1]	Onset Day[1]	Resolutio n / Death Day[1]	AE Description, Severity, and Relatedness to Study Drug	Preferred Term	System Organ Class	Outcome	Maternal Obstetric/Med History/Study Med Dose Exposures/Misc
										up at 11 months of age]	Maintained on 16-24 mg sublingual buprenorphine throughout pregnancy
	32 years	27w	39w<br>Vaginal delivery<br>Male	86	86	338	Midshaft Hypospadias<br>Grade 2 – Moderate<br>Not related	Hypospadia s	CFAGD	Recovered/ resolved	G4P2204<br>Substance History: Methamphetamine and tobacco use<br>Maintained on 24 mg daily sublingual buprenorphine throughout pregnancy
**Treatment Arm=BUP XR**											
	33 years	21 weeks (w)	40w, vaginal delivery,<br>Male	142	184	185	Pyloric Stenosis<br>Grade 3- Severe<br>Not related	Pyloric stenosis	Congenital familial and genetic disorders (CFAGD)	Recovered/ resolved	G2P2002<br>Tobacco (discontinued during pregnancy), Generalized Anxiety Disorder, hx of post-partum depression.<br>Started on 24 mg XR BUP, which was increased to 32 mg XR BUP at study week 2 and remained on this through pregnancy.
	32 years	28 w	37w5d,<br>C-section<br>Female	67	67	188	VACTERAL association<br>Grade 3- Severe<br>Not related	VACTERL syndrome	CFAGD	Recovered/ resolved	G31021<br>Prenatally diagnosed with Tetralogy of Fallot and club feet before randomization. At delivery diagnosed with patent foramen ovale. imperforate anus with vaginal fistula.<br>Medical and Substance History: Mother with hep C with high viral load, was on methadone until month 4 of pregnancy when had a return to use of fentanyl lasting 2 months, and moderate cannabis use disorder, severe sedative use (Klonopin, Xanax) disorder and use of stimulants (Adderall, cocaine) and hallucinogens (ecstasy, mushrooms, acid).<br>Started on 24 mg XR BUP, which was increased to 32 mg XR BUP at study week 2 and remained on this through pregnancy.

© 2026 Winhusen TJ et al. *JAMA Internal Medicine*.

	28 years	14 w	39w,<br>Vaginal delivery,<br>Male	175	176	-	Abnormal newborn ECHO cardiogram Grade 2- Moderate<br>Not related	Echocardio gram abnormal	Investigatio ns	Not recovered/ not resolved	G2P1102<br>Echocardiogram: No coarctation seen but cannot be ruled out. Patent ductus arteriosus and bidirectional shunting, patent foramen ovale small shunt with left to right shunting. Ventricular septal position is flattened throughout cardiac cycle. Normal right ventricular size and systolic function.<br>Substance History: Includes use of tranquilizer (Klonopin), stimulants (Adderall),cannabis (THC, hash), alcohol, IV methamphetamine, cocaine, hallucinogens (mushrooms, acid, DMT, ecstasy), inhalants (Dust-off), and tobacco. Psych hx: Generalized anxiety disorder, ADHD, bulimia, major depressive disorder<br>Mother received extended-release 24 mg for 2 weeks. She declined extended-release starting in week 3 of study and remained on non-study sublingual buprenorphine (8-16 mg daily) thereafter.
	35 years	29 w	36w,<br>C-section,<br>Male	51	51	162	Ankyloglossia/<br>congenital maxillary lip tie<br>Grade 2- Moderate<br>Not related		CFAGD	Recovered/ resolved	See below (same mother-infant)
	35 years	29 w	36w,<br>C-section,<br>Male	51	51	393	Laryngo-malacia<br>Grade 1- Mild<br>Not related		CFAGD	Recovered/ resolved	G5P4105<br>Maternal Substance History: moderate alcohol use disorder, cocaine/crack, hallucinogens, and tobacco. Also history of polydactyly, ADHD, bipolar 1 disorder.<br>Started on 24 mg XR BUP, which was increased to 32 mg XR BUP at study week 3 and remained on this through pregnancy

* Only major congenital anomalies and birth defects were reported as serious adverse events (SAEs) in this trial. Minor anomalies and birth defects were not reported. There were four major congenital anomalies reported among four infants in the sublingual buprenorphine group and five congenital anomalies reported among four infants in the extended-release group. All anomalies in both treatment groups were determined by the study site clinicians to *not* be related to study

© 2026 Winhusen TJ et al. *JAMA Internal Medicine*.

medication. This finding is supported by the fact that the mothers of these eight infants all started study medication after the first trimester, defined as the time from last menstrual period to week 13 of pregnancy, which is typically when congenital anomalies and birth defects begin. In fact, when studies look for evidence of teratogenic effects of medication exposures, they often limit exposures to the first trimester, the period devoted to organogenesis with rapid growth and development of major organ systems, as was done by a recent population-based study evaluating first trimester use of buprenorphine or methadone and risk of congenital malformations. In addition, the infant with VACTERL syndrome had evidence of multiple birth defects before randomization (VACTERL syndrome). The infant with spina bifida was diagnosed at 29 weeks EGA, two weeks after randomization in a mother without prenatal vitamins and folate; inadequate folate is a known risk factor for spina bifida. The infant with abnormal echocardiogram was exposed to study drug (extended-release buprenorphine) for only two weeks. Importantly, none of these babies died. Six of the eight affected infants recovered. The baby with biliary atresia was recovering after successful surgery including a Roux-en-Y hepaticojejunostomy, and the baby with abnormal echocardiogram had cardiology follow-up recommended at 18 months of age for further evaluation (categorized as 'not recovered, not resolved'). Severe congenital malformations may also result in stillbirth or spontaneous abortion or if diagnosed in utero may result in elective termination. There were no stillbirths or elective or spontaneous abortions in the study. There was one infant death and there was no evidence that this infant had any congenital anomaly. Overall, the sum of data support that these major anomalies are not due to study sublingual buprenorphine or extended-release exposure.

[1] Study Day is relative to first dose (Day 1 = first dose date)

© 2026 Winhusen TJ et al. *JAMA Internal Medicine*.

eTable 5. Study medication-related adverse events during pregnancy as a function of treatment group

Row Labels	Grade 1 - Mild	Grade 2 - Moderate	Grand Total
**Treatment Arm=BUP XR**			
**Gastrointestinal disorders**	**11**	**3**	**14**
Constipation	1	2	3
Nausea	6		6
Vomiting	4	1	5
**General disorders and administration site conditions**	**2**		**2**
Drug withdrawal syndrome	1		1
Fatigue	1		1
**Nervous system disorders**	**4**	**1**	**5**
Dizziness	2		2
Headache	2		2
Sedation		1	1
**Psychiatric disorders**	**3**		**3**
Insomnia	1		1
Sleep disorder	1		1
Sleep terror	1		1
**Skin and subcutaneous tissue disorders**	**2**	**2**	**4**
Alopecia		1	1
Hyperhidrosis	1		1
Night sweats	1		1
Pruritus		1	1
**Grand Total**	**22**	**6**	**28**
**Treatment Arm=BUP SL**			
**Gastrointestinal disorders**	**5**	**1**	**6**
Constipation	1	1	2
Nausea	2		2
Vomiting	2		2
**Psychiatric disorders**	**1**	**1**	**2**
Drug dependence	1	1	2
**Grand Total**	**6**	**2**	**8**

© 2026 Winhusen TJ et al. *JAMA Internal Medicine*.

eTable 6. Serious and nonserious adverse events occurring during the study by MedDRA System Organ Class*

	Maternal (Pregnancy)		Maternal (Postpartum)		Infant	
**Participants with ≥ 1 Serious Event**	Extended (N=69)	Sublingual (N=71)	Extended (N=67)	Sublingual (N=70)	Extended (N=67)	Sublingual (N=70)
Any Serious Event	**6 (8.7%)** [1]	**19 (26.8%)** [1]	**4 (6.0%)** [2]	**13 (18.6%)** [2]	9 (13.4%)	11 (15.7%)
Medication Related	0 (0.0%)	1 (1.4%)	0 (0.0%)	0 (0.0%)	0 (0.0%)	1 (1.4%)
Organized by MedDRA v27.1 System Organ Class:						
Congenital/genetic disorders	0 (0.0%)	0 (0.0%)	0 (0.0%)	0 (0.0%)	3 (4.5%)	3 (4.3%)
Gastrointestinal disorders	1 (1.4%)	0 (0.0%)	1 (1.5%)	0 (0.0%)	0 (0.0%)	0 (0.0%)
General disorders	0 (0.0%)	0 (0.0%)	0 (0.0%)	0 (0.0%)	0 (0.0%)	3 (4.3%)
Hepatobiliary disorders	0 (0.0%)	1 (1.4%)	0 (0.0%)	1 (1.4%)	2 (3.0%)	0 (0.0%)
Infections and infestations	2 (2.9%)	3 (4.2%)	1 (1.5%)	2 (2.9%)	1 (1.5%)	2 (2.9%)
Injury, poisoning and procedural complications	0 (0.0%)	2 (2.8%)	0 (0.0%)	1 (1.4%)	0 (0.0%)	0 (0.0%)
Investigations	0 (0.0%)	1 (1.4%)	0 (0.0%)	0 (0.0%)	1 (1.5%)	0 (0.0%)
Metabolism and nutrition disorders	0 (0.0%)	0 (0.0%)	0 (0.0%)	1 (1.4%)	2 (3.0%)	0 (0.0%)
Musculoskeletal and connective tissue disorders	1 (1.4%)	0 (0.0%)	0 (0.0%)	0 (0.0%)	0 (0.0%)	0 (0.0%)
Nervous system disorders	0 (0.0%)	0 (0.0%)	0 (0.0%)	1 (1.4%)	0 (0.0%)	0 (0.0%)
Pregnancy, puerperium and perinatal conditions	4 (5.8%)	8 (11.3%)	0 (0.0%)	4 (5.7%)	0 (0.0%)	1 (1.4%)
Psychiatric disorders	1 (1.4%)	6 (8.5%)	3 (4.5%)	4 (5.7%)	0 (0.0%)	0 (0.0%)
Renal and urinary disorders	0 (0.0%)	0 (0.0%)	0 (0.0%)	0 (0.0%)	0 (0.0%)	2 (2.9%)
Respiratory, thoracic and mediastinal disorders	0 (0.0%)	1 (1.4%)	0 (0.0%)	1 (1.4%)	0 (0.0%)	0 (0.0%)
Surgical and medical procedures	0 (0.0%)	3 (4.2%)	0 (0.0%)	1 (1.4%)	0 (0.0%)	0 (0.0%)
**Participants with At Least One Nonserious TEAE**					Not tracked in infants	
Any Nonserious TEAE	44 (63.8%)	47 (66.2%)	37 (55.2%)	45 (64.3%)		
Organized by MedDRA v27.1 System Organ Class[3]:						
Gastrointestinal disorders	21 (30.4%)	16 (22.5%)	11 (16.4%)	13 (18.6%)		
General disorders and administration site conditions	7 (10.1%)	3 (4.2%)	6 (9.0%)	2 (2.9%)		
Infections and infestations	13 (18.8%)	20 (28.2%)	22 (32.8%)	27 (38.6%)		
Injury, poisoning and procedural complications	4 (5.8%)	4 (5.6%)	6 (9.0%)	7 (10.0%)		
Investigations	1 (1.4%)	4 (5.6%)	1 (1.5%)	1 (1.4%)		
Musculoskeletal and connective tissue disorders	1 (1.4%)	5 (7.0%)	1 (1.5%)	3 (4.3%)		
Nervous system disorders	8 (11.6%)	6 (8.5%)	6 (9.0%)	9 (12.9%)		
Pregnancy, puerperium and perinatal conditions	11 (15.9%)	20 (28.2%)	1 (1.5%)	5 (7.1%)		
Psychiatric disorders	5 (7.2%)	7 (9.9%)	10 (14.9%)	10 (14.3%)		
Reproductive system and breast disorders	1 (1.4%)	4 (5.6%)	4 (6.0%)	7 (10.0%)		
Respiratory, thoracic and mediastinal disorders	2 (2.9%)	6 (8.5%)	4 (6.0%)	4 (5.7%)		

© 2026 Winhusen TJ et al. *JAMA Internal Medicine*.

	Maternal (Pregnancy)		Maternal (Postpartum)		Infant	
	Extended	Sublingual	Extended	Sublingual	Extended	Sublingual
Skin and subcutaneous tissue disorders	8 (11.6%)	4 (5.6%)	2 (3.0%)	4 (5.7%)		
Vascular disorders	2 (2.9%)	1 (1.4%)	4 (6.0%)	2 (2.9%)		

*Extended denotes extended-release buprenorphine; Sublingual denotes sublingual buprenorphine, Maternal adverse and serious adverse events occurred in the safety window, which began at the first dose date and ended either 7 days after the last BUP-SL dose, 7 days after the last weekly extended-release dose, or 28 days after the last monthly extended-release dose; An alpha level of 0.05 was selected for each test of significance. [1] p<0.01; [2]p<0.05; [3]The adverse events reported here are events that occurred in 5% or more of participants.

© 2026 Winhusen TJ et al. *JAMA Internal Medicine*.

eTable 7. Summary of study injection site examinations

	Grade 1 – Mild	Grade 2 – Moderate	Grade 3 - Severe	Total
Number of injections				2476
Number of injection site examinations[1]				2384/2476 (96.3%)
Number of abnormal injection sites[2]				349/2384 (14.6%)
Symptom experienced	569	152	5	726
Pain	68/569 (12.0%)	12/152 (7.9%)	1/5 (20.0%)	81/726 (11.2%)
Itching	98/569 (17.2%)	20/152 (13.2%)	2/5 (40.0%)	120/726 (16.5%)
Discharge	0/569 (0%)	0/152 (0%)	0/5 (0%)	0/726 (0%)
Tenderness	99/569 (17.4%)	18/152 (11.8%)	1/5 (20.0%)	118/726 (16.3%)
Erythema/Redness	56/569 (9.8%)	46/152 (30.3%)	0/5 (0%)	102/726 (14.0%)
Swelling	47/569 (8.3%)	21/152 (13.8%)	1/5 (20.0%)	69/726 (9.5%)
Induration	152/569 (26.7%)	25/152 (16.4%)	0/5 (0%)	177/726 (24.4%)
Abscess	0/569 (0%)	0/152 (0%)	0/5 (0%)	0/726 (0%)
Ulceration	0/569 (0%)	0/152 (0%)	0/5 (0%)	0/726 (0%)
Necrosis	0/569 (0%)	0/152 (0%)	0/5 (0%)	0/726 (0%)
Other	49/569 (8.6%)	10/152 (6.6%)	0/5 (0%)	59/726 (8.1%)

[1] The numerator is the number of injection site examinations that were conducted. The denominator is the number of injections.

[2] The numerator is the number of injection site reactions. The denominator is the number of injection site examinations.

© 2026 Winhusen TJ et al. *JAMA Internal Medicine*.

eTable 8. Injection site reactions by severity and time to resolution

	Grade 1-Mild					Grade 2 - Moderate					Grade 3 - Severe					Grand Total
	Weeks to Resolve[1]				Total	Weeks to Resolve[1]				Total	Weeks to Resolve[1]				Total	
**Row Labels**	**1 week**	**2 weeks**	**3 weeks**	**4+ weeks**		**1 week**	**2 weeks**	**3 weeks**	**4+ weeks**		**1 week**	**2 weeks**	**3 weeks**	**4+ weeks**		
Erythema/Redness	27	11	10	8	56	23	9	9	5	46						102
Induration	40	54	23	35	152	6	2	7	10	25						177
Itching	71	12	9	6	98	9	7	2	2	20	1			1	2	120
Other: Bruise	3	1			4											4
Other: Bruise/ Discoloration		1			1											1
Other: Bruised		1			1											1
Other: bruising	8	7	6	2	23	1	2		2	5						28
Other: burning	1				1											1
Other: Ecchymosis	1	3		2	6	1	1			2						8
Other: ecchymosis (bruise)	1				1											1
Other: eccymosis									1	1						1
Other: hyperpigmentation	1				1											1
Other: lump									1	1						1
Other: Nodule									1	1						1
Other: petechiae		1			1											1
Other: rash	1				1											1
Other: Stinging	7	2			9											9
Pain	43	12	9	4	68	8	2	1	1	12				1	1	81
Swelling	34	8	3	2	47	8	8	3	2	21	1				1	69
Tenderness	61	16	13	9	99	9	5	2	2	18	1				1	118
**Grand Total**	**299**	**129**	**73**	**68**	**569**	**65**	**36**	**24**	**27**	**152**	**3**			**2**	**5**	**726**

[1]Injection site reaction resolved within the listed number of weeks

© 2026 Winhusen TJ et al. *JAMA Internal Medicine*.

eTable 9. Other maternal safety outcomes as a function of phase and treatment group*

	Study Treatment Group		Effect Size[1]	P
	Buprenorphine Extended-Release	Buprenorphine Sublingual		
Primary cesarean[1], %	22.7% ± 5.2%	20.0% ± 4.8%	RR = 1.1 (0.6, 2.0)	0.70
Cesarean[1], %	40.9% +- 6.1%	44.3% +- 5.9%	RR = 0.9 (0.6, 1.3)	0.69
Abnormal fetal presentation during delivery[2], %	15.4% ± 4.5%	18.6% ± 4.6%	RR = 0.8 (0.4, 1.7)	0.62
Medical complications during labor[1], %	22.7% ± 5.2%	32.9% ± 5.6%	RR = 0.7 (0.4, 1.2)	0.19
Anesthesia during labor or delivery receipt[3], %	100.0% +- 0.6%	100.0% +- 0.7%	RR = 1.0 (1.0, 1.0)	0.96
Local anesthesia	0.0% +- 2.6%	0.0% +- 3.1%	RR = 0.8 (0.2, 3.2)	0.79
Epidural	82.9% +- 232.3%	76.9% +- 163.2%	RR = 1.0 (0.8, 1.2)	0.74
Spinal block	17.3% +- 234.2%	21.6% +- 155.7%	RR = 1.0 (0.5, 1.8)	0.93
General anesthesia	0.7% +- 73.3%	0.5% +- 48.7%	RR = 1.4 (0.5, 4.3)	0.51
Pain management receipt, %				
During labor or delivery[4]	53.1% ± 6.2%	55.4% ± 6.2%	RR = 1.0 (0.7, 1.3)	0.80
Opioid	46.9% ± 6.2%	49.2% ± 6.2%	RR = 1.0 (0.6, 1.3)	0.79
Non-opioid	23.4% ± 5.3%	27.7% ± 5.6%	RR = 0.8 (0.4, 1.5)	0.58
Other[5]	0.0% ± 0.0%	3.1% ± 2.1%	NA[6]	
During postpartum hospital stay[7]				
Non-opioid	82.5% ± 4.8%	92.4% ± 3.3%	RR = 0.9 (0.7, 1.0)	0.10
Other[8]	12.7% ± 4.2%	6.1% ± 2.9%	RR = 2.1 (0.7, 5.6)	0.20
Prescribed at time of discharge[7]	85.7% ± 4.4%	90.9% ± 3.5%	RR = 0.9 (0.7, 1.0)	0.36
Opioid	20.6% ± 5.1%	25.8% ± 5.4%	RR = 0.8 (0.4, 1.5)	0.49
Non-opioid	82.5% ± 4.8%	83.3% ± 4.6%	RR = 1.0 (0.8, 1.1)	0.90
Other[9]	9.5% ± 3.7%	4.5% ± 2.6%	RR = 2.1 (0.5, 6.8)	0.28
Pregnancy Phase:				
Opioid Overdose[10], %	0.0% ± 0.0%	1.4% ± 1.4%	NA[6]	
HADS Depression total score[7]	4.1 ± 0.3	5.0 ± 0.3	-0.6 (-1.6, 0.3)	0.19
HADS Anxiety total score[7]	7.0 ± 0.4	7.3 ± 0.3	-0.4 (-1.3, 0.6)	0.47
Postpartum Phase:				
Opioid Overdose[11], %	4.5% ± 2.6%	0.0% ± 0.0%	NA[6]	
HADS Depression total score[11]	3.5 ± 0.3	4.3 ± 0.3	-0.8 (-1.6, 0.1)	0.09

© 2026 Winhusen TJ et al. *JAMA Internal Medicine*.

* Plus–minus values are estimated means ±SE. [1]Effect size denotes difference in estimated means; OR=Odds Ratio; RR=Relative Risk; Confidence interval matches the alpha for statistical testing (e.g., 95% CI for α =.05; 98.33% for α =0.0167, etc.); [2] Data is truncated after medication disruption for impacted extended-release buprenorphine participants; [1]Extended-release n=66; Sublingual n=70; [2]Extended-release n=65; Sublingual n=70; [3]Extended-release n=63; Sublingual n=65; [4]Extended-release n=64; Sublingual n=65; [5]Other=Flexeril and lidocaine-epinephrine; [6]Data insufficient to support analysis; [7]Extended-release n=63; Sublingual n=66; [8]Other=Acetaminophen, celecoxib, cyclobenzaprine, dibucaine, gabapentin, ibuprofen, ketamine, pregabalin; [9]Other=Acetaminophen, celecoxib, cyclobenzaprine, dibucaine, gabapentin, ibuprofen, ketamine, lyrica; acetaminophen, dibucaine, flexeril, gabapentin, ibuprofen, pregabalin; [10]Extended-release n=67; Sublingual n=70; [11]Extended-release n=66; Sublingual n=67

© 2026 Winhusen TJ et al. *JAMA Internal Medicine*.

eTable 10. Other infant safety outcomes as a function of phase and treatment group*

	Study Treatment Group		Effect Size[1]	P
	Buprenorphine Extended-Release (N=66)	Buprenorphine Sublingual (N=69)		
Live birth, %	100% ± 0%±	100% ± 0%	NA[2]	
Weight at birth[3], g.	3163.4 ± 71.1	3005.7 ± 69.0	157.7 (-38.3, 353.6)	0.11
Length at birth[4], cm.	49.4 ± 0.4	48.9 ± 0.3	0.4 (-0.6, 1.4)	0.39
Gestational age at delivery, weeks	38.2 ± 0.2	37.7 ± 0.2	0.5 (-0.2, 1.2)	0.13
Apgar score[5]				
1 min	7.5 ± 0.2	7.7 ± 0.2	-0.3 (-0.8, 0.2)	0.28
5 min	8.5 ± 0.1	8.7 ± 0.1	-0.2 (-0.5, 0.1)	0.20
Abnormal conditions[3]-, %	47.7% ± 6.2%	39.1% ± 5.9%	RR = 1.2 (0.8, 1.7)	0.32
Respiratory distress	29.2% ± 5.6%	24.6% ± 5.2%	RR = 1.2 (0.7, 1.9)	0.55
Feeding problems	17.3% ± 4.8%	12.0% ± 4.0%	RR = 1.4 (0.6, 2.9)	0.43
Sepsis	4.6% ± 2.6%	0.0% ± 0.0%	NA[2]	
Seizure or serious neurologic dysfunction	0.0% ± 0.0%	1.4% ± 1.4%	NA[2]	
Significant birth injury	3.1% ± 2.1%	0.0% ± 0.0%	NA[2]	
Other	24.6% ± 5.3%	15.9% ± 4.4%	RR = 1.5 (0.8, 2.7)	0.21
Interventions for abnormal Conditions[3], %	52.3% ± 6.2%	43.5% ± 6.0%	RR = 1.2 (0.8, 1.6)	0.31
Resuscitation required immediately following delivery	12.3% ± 4.1%	2.9% ± 2.0%	RR = 4.2 (0.9, 14.2)	0.06
Assisted ventilation required immediately following delivery	23.1% ± 5.2%	23.2% ± 5.1%	RR = 1.0 (0.5, 1.7)	0.99
Assisted ventilation > six hours	10.8% ± 3.8%	11.6% ± 3.9%	RR = 0.9 (0.3, 2.3)	0.88
Surfactant replacement therapy	3.1% ± 2.1%	1.4% ± 1.4%	RR = 2.1 (0.2, 18.5)	0.53
NICU admission	43.1% ± 6.1%	31.9% ± 5.6%	RR = 1.4 (0.8, 1.9)	0.18
Antibiotics for suspected neonatal sepsis	9.2% ± 3.6%	10.1% ± 3.6%	RR = 0.9 (0.3, 2.4)	0.86
Feeding (NG) tube inserted	13.8% ± 4.3%	13.0% ± 4.1%	RR = 1.1 (0.4, 2.3)	0.89
Preterm, < 37 wks., %	12.1% ± 4.0%	21.7% ± 5.0%	RR = 0.6 (0.2, 1.2)	0.14
Fetal distress	4.5% ± 2.6%	5.8% ± 2.8%	RR = 0.8 (0.2, 3.2)	0.74
Premature placental abruption	0.0% ± 0.0%	4.3% ± 2.5%	NA[2]	

© 2026 Winhusen TJ et al. *JAMA Internal Medicine*.

	Study Treatment Group		Effect Size[1]	P
	Buprenorphine Extended-Release (N=66)	Buprenorphine Sublingual (N=69)		
Maternal trauma	0.0% ± 0.0%	1.4% ± 1.4%	NA[2]	
Other	6.1% ± 2.9%	14.5% ± 4.2%	RR = 0.4 (0.1, 1.2)	0.12
Infant discharged alive	100% ± 0%±	100% ± 0%	NA[2]	
Infant Sedation[6], %	32.3% ± 6.7%	25.7% ± 6.2%	RR = 1.2 (0.6, 2.0)	0.57
Did not wake up for feeding	13.7% ± 4.8%	3.8% ± 2.7%	RR = 3.6 (0.8, 11.7)	0.10
Any difficulty breathing	23.5% ± 5.9%	15.4% ± 5.0%	RR = 1.5 (0.7, 3.0)	0.30
Felt limp when held	13.7% ± 4.8%	17.3% ± 5.2%	RR = 0.8 (0.3, 1.9)	0.62

* Plus–minus values are estimated means ±SE. [1]Effect size denotes difference in estimated means; RR=Relative Risk; Confidence interval matches the alpha for statistical testing (e.g., 95% CI for α =.05; 98.33% for α =0.0167, etc.); [2]Data insufficient to support analysis; [3]Extended-release n=65; Sublingual n=69; [4]Extended-release n=63; Sublingual n=67; [5]Extended-release n=64; Sublingual n=69; [6]For infants receiving breastmilk (Extended-release n=51, Sublingual n=52)

© 2026 Winhusen TJ et al. *JAMA Internal Medicine*.
