# Supplementary material for: Extended-Release vs Sublingual Buprenorphine in Pregnancy Through 12 Months Post Partum: A Randomized Clinical Trial
Source: JAMA Intern Med. 2026 Mar 16;186(5):533–43. doi: 10.1001/jamainternmed.2026.0057 (PMC12993732; doi:10.1001/jamainternmed.2026.0057)
Supplement: Supplement 3. — Data Sharing Statement [file jamainternmed-e260057-s003.pdf]

Data Sharing Statement

Winhusen. Extended-Release vs Sublingual Buprenorphine in Pregnancy Through 12 Months Post Partum. *JAMA Intern Med*. Published March 16, 2026.
doi:10.1001/jamainternmed.2026.0057

Data
Additional Information: Trial Registration: ClinicalTrials.gov Identifier: NCT03918850.
Data available: Yes
Data types: Deidentified participant data
How to access data: Data from this HEAL-funded National Drug Abuse Treatment Clinical Trials Network (CTN) trial will be posted to https://datashare.nida.nih.gov/ in March of 2026. All of the data will be de-identified. Data documentation, consisting of all annotated case report forms (CRFs), the data dictionary, and de-identification notes, will be provided to users to assist in data interpretation. Protocol documentation, including a brief study description, the study protocol, and a link to the primary manuscript, will also be provided, and users will be encouraged to consult these documents for insight regarding proper interpretation of the data
When available: With publication

Supporting Documents
Document types: Other (please specify)
Additional Information: All of the data will be de-identified. Data documentation, consisting of all annotated case report forms (CRFs), the data dictionary, and de-identification notes, will be provided to users to assist in data interpretation. Protocol documentation, including a brief study description, the study protocol, and a link to the primary manuscript, will also be provided, and users will be encouraged to consult these documents for insight regarding proper interpretation of the data
How to access documents: Data from this HEAL-funded National Drug Abuse Treatment Clinical Trials Network (CTN) trial will be posted to https://datashare.nida.nih.gov/ in March of 2026.
When available: With publication

Additional Information
Who can access the data: The de-identified data will be available to everyone
Types of analyses: Any analyses that can be completed with de-identified data.
Mechanisms of data availability: Data from this HEAL-funded National Drug Abuse Treatment Clinical Trials Network (CTN) trial will be posted to https://datashare.nida.nih.gov/ in March of 2026
